# Synthesis, Characterization and Reactivity of Alkyl Iron(II) PCP Pincer Complexes

**DOI:** 10.1021/acs.organomet.6c00256

**Published:** 2026-08-28

**Authors:** Matthias Käfer, Simon Gstöttner, Berthold Stöger, Antoine Dupé, Luis F. Veiros, Karl Kirchner

**Affiliations:** † Institute of Applied Synthetic Chemistry, TU Wien, Getreidemarkt 9/163-AC, 1060 Wien, Austria; ‡ X-Ray Center, TU Wien, Getreidemarkt 9/163, 1060 Wien, Austria; § Inorganic Chemistry, Institute of Chemistry, University of Graz, 8010 Graz, Austria; ∥ Centro de Química Estrutural, Institute of Molecular Sciences, Departamento de Engenharia Química, Instituto Superior Técnico, 72971 Universidade de Lisboa, Av. Rovisco Pais, 1049-001 Lisboa, Portugal

## Abstract

The syntheses and reactivity of a series of alkyl iron PCP pincer complexes is described. The alkyl complexes of the type [Fe­(PCP^CH2^-*i*Pr)­(R)­(CO)_2_] (R = CH_3_, CH_2_CH_3_, CH_2_CH_2_CH_3_, CH_2_CH_2_CH_2_CH_3_) are accessible in good yields (69–78%) by reduction of [Fe­(PCP^CH2^-*i*Pr)­(Br)­(CO)_2_] with sodium followed by oxidative addition of alkyl halides RX (X = Br or I). As alkyl complexes are typically prone to undergo migratory insertion reactions in the presence of potential strong ligands, [Fe­(PCP^CH2^-*i*Pr)­(CH_2_CH_2_CH_3_)­(CO)_2_] was treated with CO and CN*t*Bu affording the acyl complexes [Fe­(PCP^CH2^-*i*Pr)­((C=O)–CH_2_CH_2_CH_3_)­(CO)_2_] and [Fe­(PCP^CH2^-*i*Pr)­(C=O)–CH_2_CH_2_CH_3_)­(CO)­(CN*t*Bu)] in 98 and 91% yields. Upon treatment of [Fe­(PCP^CH2^-*i*Pr)­((C=O)–CH_2_CH_2_CH_3_)­(CO)_2_] with pinacolborane (HBpin) the hydride complex [Fe­(PCP^CH2^-*i*Pr)­(H)­(CO)_2_] is obtained. When [Fe­(PCP^CH2^-*i*Pr)­(CH_2_CH_2_CH_3_)­(CO)_2_] is reacted with an excess (4 equiv) of HBpin and SiH_2_Ph_2_, complexes [Fe­(PCP^CH2^-*i*Pr)­(κ^2^ *H,H*-H_2_BPin)­(CO)] and [Fe­(PCP^CH2^-*i*Pr)­(SiHPh_2_)­(CO)], respectively, is formed. The first is a very labile complex readily liberating HBpin thereby undergoing decomposition to intractable materials. The latter is an unusually stable 16e^–^ complex. Finally, in a preliminary reactivity study [Fe­(PCP^CH2^-*i*Pr)­(CO)_2_(CH_2_CH_2_CH_3_)] is demonstrated to be an active precatalyst for the hydroboration of nitriles with HBPin. The hydroboration reaction requires no additives and proceeds with a catalyst loading of 2 mol % at 60 °C. The reduction of nitriles and subsequent hydrolysis provide ready access to aminomethylene units in organic molecules.

## Graphical abstract

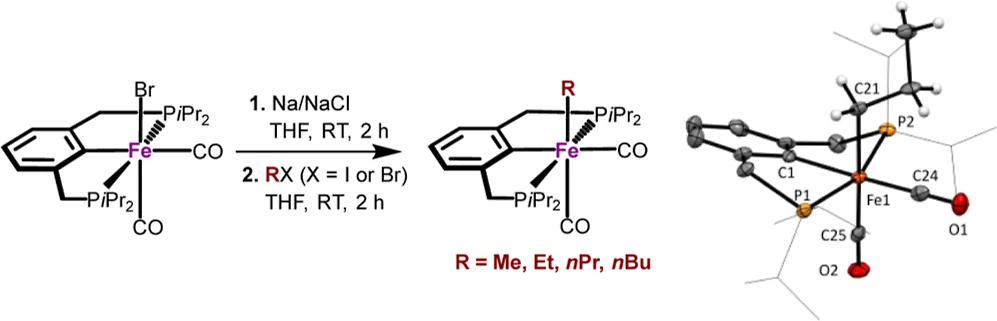

## Introduction

Migratory insertion of CO into a transition metal alkyl bond is a fundamental step in many transition-metal-catalyzed processes, playing a central role in the formation of carbon–carbon and carbon–heteroatom bonds. Our previous work focused on the synthesis and reactivity manganese alkyl carbonyl complexes. We took advantage of the fact that Mn­(I)-alkyl carbonyl complexes undergo migratory insertion of the nucleophilic alkyl ligand into the polarized CO moiety yielding a coordinatively unsaturated acyl complex, which is capable of activating nonpolar and weakly polar E–H bonds (E = –H, –C≡C–R, -BR_2_, -SiR_3_) revealing great potential in catalysis such as the hydrogenation of nitriles, ketones, CO_2_, alkenes and alkynes, dehydrogenative silylation, hydrosilylation and hydroboration of alkynes.^1,2^ The rate of alkyl migration typically follows the order *n*Pr > Et > Me as already shown by Moss and co-workers some years ago.^3^

In the present paper, we apply this concept to iron alkyl carbonyl complexes supported by a rigid anionic PCP pincer ligand framework where two di­(isopropyl)­phosphine moieties are tethered via CH_2_–linkers to the benzene backbone in the two *ortho* positions. Specifically, we aimed to determine whether well-defined alkyl carbonyl complexes of the type [Fe­(PCP^CH2^-*i*Pr)­(alkyl)­(CO_2_)] are synthetically accessible and whether they are capable of undergoing intramolecular migratory insertion. It has to be noted that anionic PCP ligands, where phosphine donors are tethered via CH_2_–, NH-, NR- or O-linkers to a benzene backbone are still one of most common pincer motifs. Over the years several Fe­(II) PCP pincer complexes were described and applications in catalysis were reported (Scheme 1)^4^ ^,^ ^5^ For instance, Guan and co-workers synthesized a variety of PCP based Fe­(II) hydride complexes and successfully employed them as catalysts for the hydrosilylation of ketones,^5a^ and also studied the catalytic ammonia-borane dehydrogenation mechanistically.^5b^ In 2017, Sortais and co-workers developed a set of Fe­(II) PCP hydrides, which were able to catalyze the hydroboration of styrene under UV radiation at room temperature.^6^ In the same year, Milstein and co-workers described the synthesis and reactivity of a Fe­(II) PCP pincer system where the benzene backbone features a hydroxy group in the *para* position.^7^ This system allowed cooperative activation of small molecules like molecular hydrogen, oxygen, and bromine and was active for the dehydrogenation of alcohols via ferraquinone-ferrahydroquinone organometallic redox couple. Our group reported the synthesis of a series of iron PCP pincer complexes. Interestingly, PCP complexes where the phosphine donors were tethered via NEt-linkers to the benzene backbone were able to stabilize the iron center in three different formal oxidation states (0,+I, and + II).^8^

**Scheme 1:**
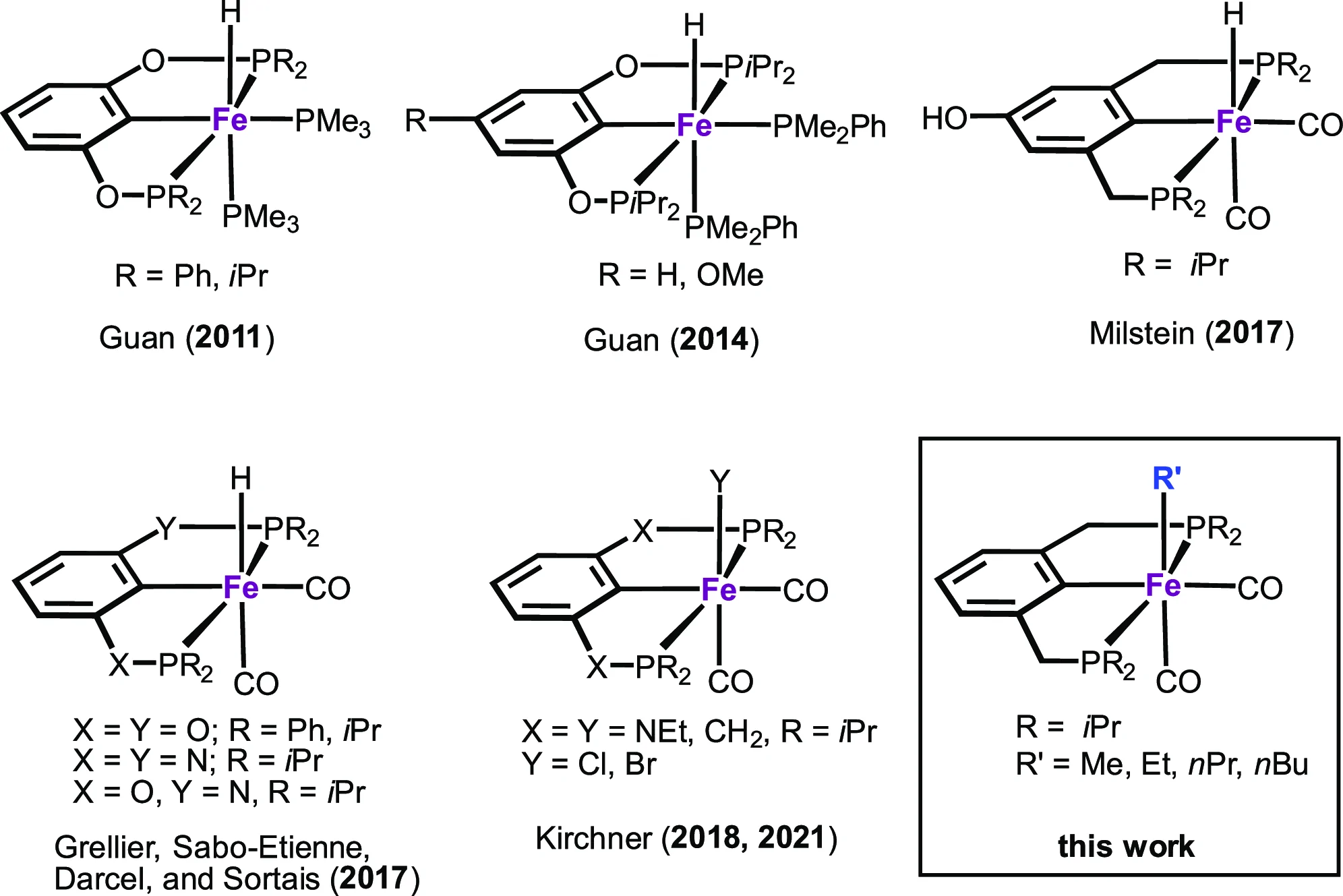
Fe­(II) PCP Pincer Complexes Bearing a Benzene Backbone

Finally, it has to be noted that the synthesis of an Fe­(II) PCP alkyl carbonyl complex was recently reported by our group.^9^ This complex was catalytically active for the *semi*-hydrogenation of alkynes^9a^ and the hydrosilylation of alkynes with SiH_3_Ph to yield vinylsilanes.^9b^ The PCP ligand in this case was a neutral pyrazole-derived pincer ligand containing a 1-methylpyrazole backbone tethered to two di­(isopropyl)­phosphine moieties via phenylene spacers and is thus not comparable to the PCP ligand utilized in this work.^10^

## Results and Discussion

The alkyl carbonyl Fe­(II) PCP pincer complexes [Fe­(PCP^CH2^-*i*Pr)­(R)­(CO)_2_] (R = CH_3_ (**3a**), CH_2_CH_3_ (**3b**), CH_2_CH_2_CH_3_ (**3c**), CH_2_CH_2_CH_2_CH_3_ (**3d**)) were obtained by treatment of [Fe­(PCP^CH2^-*i*Pr)­(Br)­(CO)_2_] (**1**) with Na/NaCl (mass ratio 1:1) and subsequent addition of CH_3_I, CH_3_CH_2_Br, and CH_3_CH_2_CH_2_Br, and CH_3_CH_2_CH_2_CH_2_Br, respectively, in 65 to 78% isolated yields (Scheme 2). The reaction presumably proceeds *via* the air-sensitive anionic complex [Fe­(PCP^CH2^-*i*Pr)­(CO)_2_]^−^ (**2**) which was not isolated (Scheme 2). All alkyl complexes are bench-stable in the solid state for several weeks in the presence of air. These complexes were fully characterized by a combination of ^1^H, ^13^C­{^1^H}, and ^31^P­{^1^H} NMR spectroscopy, IR spectroscopy, and HR-MS analysis. The ^13^C­{^1^H} NMR spectra of complexes **3a**–**d** are very similar exhibiting two characteristic low field triplet resonances at about 214 ppm (*J*_*PC*_ = 15 Hz) and 219 ppm (*J*_*PC*_ = 18 Hz) that can be assigned to the carbonyl ligands. The *ipso* carbon was detected in the range of 180 to 178 ppm (*J*_*PC*_ = 15 Hz). The α-carbon atoms of the alkyl chain give rise to triplet resonances centered at −6.0 (**3a**), 3.2 (**3b**), 14.3 (**3c**) and 10.9 ppm (**3d**) with *J*_*PC*_ in the range of 19–20 Hz The ^31^P­{^1^H} NMR spectra show singlet resonances in the narrow range of 97 to 94 ppm. In the IR spectrum, all complexes exhibit two strong absorption bands at about 1968 and 1907 cm^–1^ indicating a *cis* arrangement of the CO ligands. In addition, the molecular structure of **3c** was determined by X-ray crystallography. A structural view is depicted in Figure 1 with selected bond distances and angles given in the caption.

**Scheme 2:**
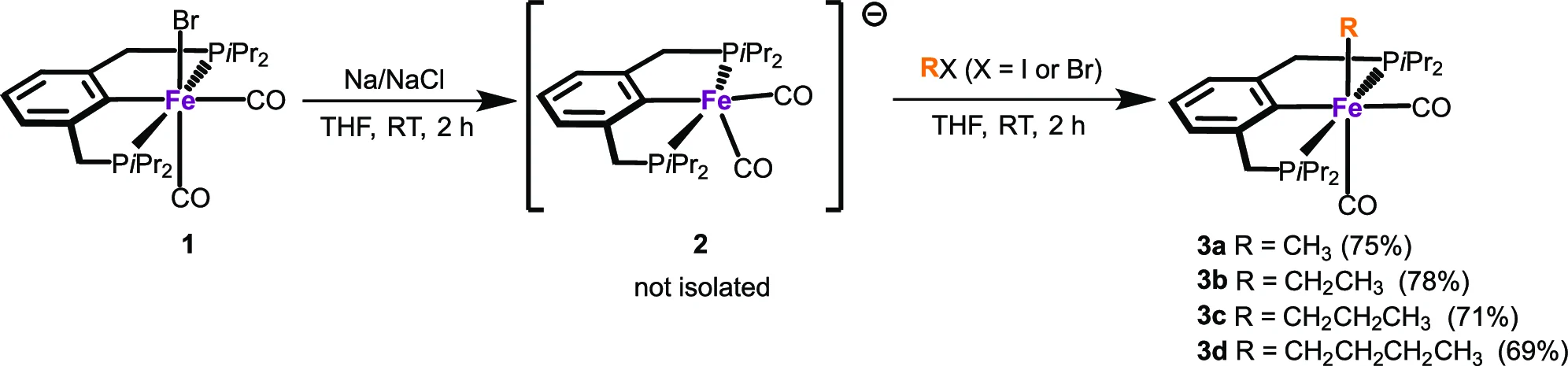
Synthesis of Alkyl Fe­(II) PCP Complexes

**Figure 1:**
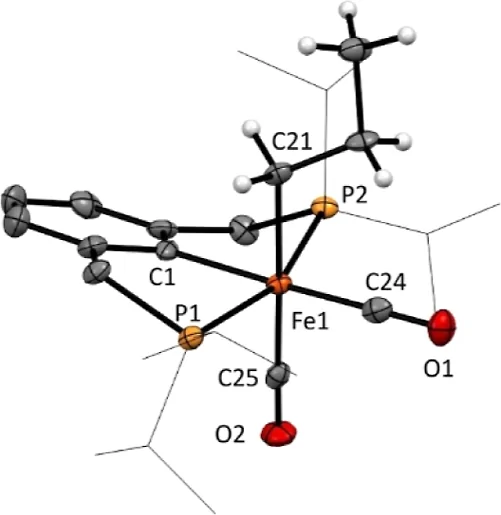
Structural view of [Fe­(PCP^CH2^-*i*Pr)­(CH_2_CH_2_CH_3_)­(CO)_2_] (**3c**) showing 50% thermal ellipsoids (H-Atoms omitted for clarity). Selected bond-lengths (Å) and angles (°): Fe1–C1 2.055(7), Fe1–C21 2.114(7), Fe1–C24 1.780(8), Fe1–C25 1.782(7), Fe1–P2 2.244(2), Fe1–P1 2.2416(18), P1–Fe1–P2 159.61(8), C24–Fe1–C1 178.3(3), C25–Fe1–C21 178.2(3), C24–Fe1–C25 93.7(3).

This complex adopts a slightly distorted octahedral geometry. In particular the P–Fe–P angles deviate significantly from 180° being 159.61(8)°. The C_ipso_-Fe and Fe-alkyl (Fe–C21) bond lengths are 2.055(7) and 2.114(7) Å, respectively. As expected, the two Fe–C_CO_ bond distances Fe1–C24 and Fe1–C25 are considerably shorter being 1.780(8) and 1.782(7) Å, respectively, which illustrates a pronounced π-backbonding that directly correlates with the electronic enrichment from the PCP pincer scaffold.

In order to determine whether migratory insertion of one CO ligand into the iron-alkyl can be initiated in the presence of strong ligands, complex **3c** was treated with CO gas at ambient pressure for 10 min resulting in the formation of the Fe­(II) acyl complex [Fe­(PCP^CH2^-*i*Pr)­(C­(=O)­CH_2_CH_2_CH_3_)­(CO)_2_] (**4**) in 98% isolated yield (Scheme 3). Likewise, treatment of **3c** with CN*t*Bu led to the formation of [Fe­(PCP^CH2^-*i*Pr)­(C­(=O)­CH_2_CH_2_CH_3_)­(CO)­(CN*t*Bu)] (**5**) in 91% isolated yield (Scheme 3). Both complexes were characterized by a combination of ^1^H, ^13^C­{^1^H} and ^31^P­{^1^H} NMR spectroscopy, IR spectroscopy as well as high-resolution mass spectrometry. The IR spectrum of **4** contains two strong stretching vibrations of the CO ligands at 1972 and 1911 cm^–1^ and a strong resonance at 1615 cm^–1^ assignable to the acyl C=O moiety. Accordingly, the two CO ligands adopt a *cis* arrangement. In the case of **5**, strong absorptions were found at 2098 (ν_CN*t*Bu_), 1901 (ν_CO_), and 1591 (ν_C=O_) cm^–1^ which clearly indicates the coordination of one isocyanide, one CO and an acyl ligand, respectively.

**Scheme 3:**
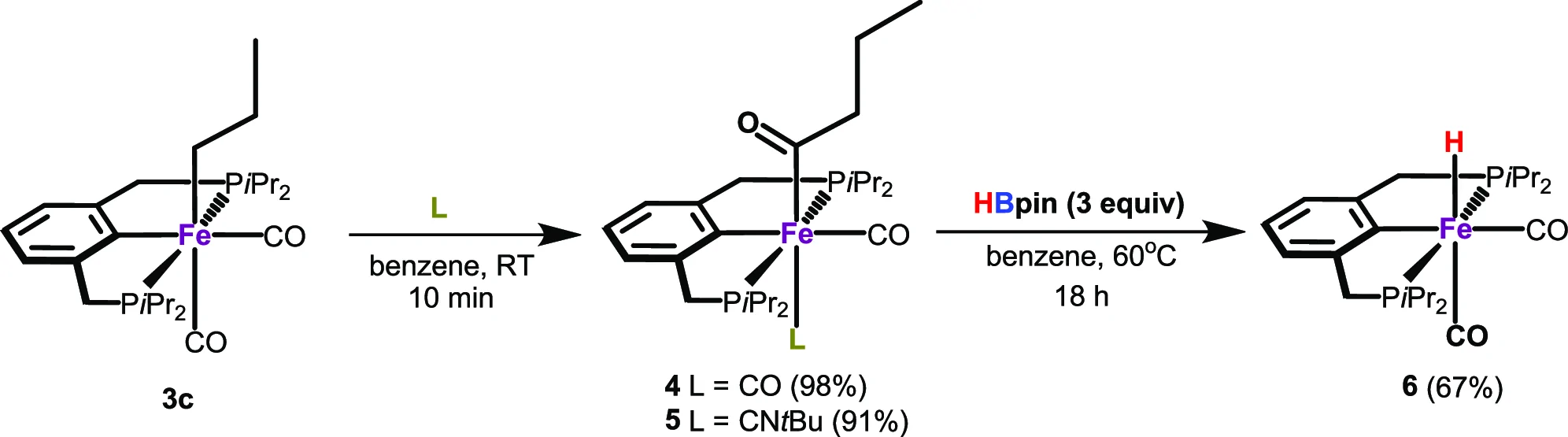
Trapping of Fe­(II) PCP Acyl Complexes and Synthesis of an Fe­(II) Hydride Complex

Additionally, the molecular structures of **4** and **5** were established by X-ray crystallography. Structural views are depicted in Figures 2 and 3 with selected bond distances and angles given in the captions. The coordination geometry around the iron centers corresponds to a distorted octahedron. In the case of **5**, the acyl moiety is located *trans* to the isocyanide ligand. The carbonyl-iron-acyl and isocyanide-iron-acyl angles *trans* to one another deviate only slightly from 180° being 174.12(6) and 175.3(2), respectively.

**Figure 2:**
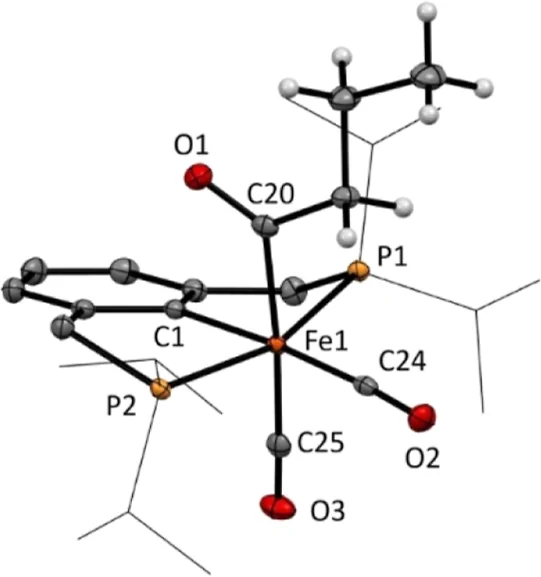
Structural view of [Fe­(PCP^CH2^-*i*Pr)­(C­(=O)–CH_2_CH_2_CH_3_)­(CO)_2_] (**4**). showing 50% thermal ellipsoids (H-Atoms omitted for clarity). Selected bond-lengths (Å) and angles (°): Fe1–C1 2.047(2), Fe1–P1 2.2371(4), Fe1–P2 2.2229(4), Fe1–C20 2.032(2), Fe1–C24 1.784(2), Fe1 C25 1.791(2), P2–Fe1–P1 161.14(2), C24–Fe1–C1 174.79(6), C25–Fe1–C20 174.12(6), C24–Fe1–C25 93.07(7).

**Figure 3:**
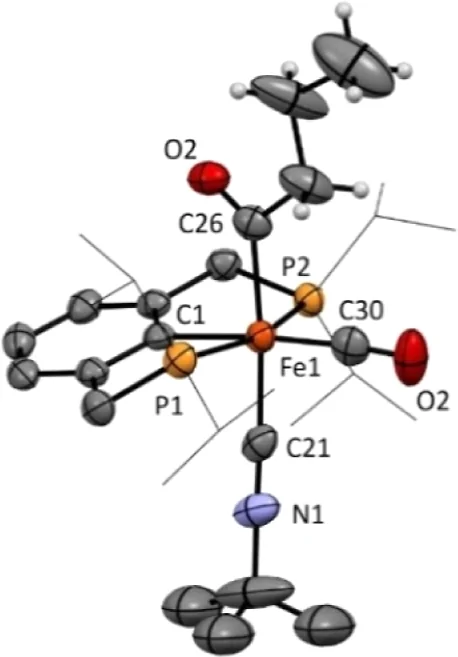
Structural view of [Fe­(PCP^CH2^-*i*Pr)­(C­(=O)–CH_2_CH_2_CH_3_)­(CO)­(CN*t*Bu)] (**5**). showing 50% thermal ellipsoids (H-Atoms omitted for clarity). Selected bond-lengths (Å) and angles (°): Fe1–P1 2.235(1), Fe1–P2 2.229(1), Fe1–C1 2.033(4), Fe1–C21 1.866(5), Fe1–C26 2.000(5), Fe1–C30 1.756(5), P2–Fe1–P1 162.72(5), C21–Fe1–C26 175.3(2), C30–Fe1–C1 174.7(2).

When **4** was heated to 60 °C in the presence of HBpin for 18 h, the dicarbonyl hydride complex [Fe­(PCP^CH2^-*i*Pr)­(H)­(CO)_2_] (**6**) was obtained in 67% isolated yield (Scheme 3). It has to be mentioned that upon reacting [Fe­(PCP^CH2^-*i*Pr)­(Br)­(CO)_2_] (**1**) directly with a hydride source such as Li­[HBEt_3_] no hydride complex was obtained. Instead, a formal one electron reduction of the complex took place and the green air-sensitive Fe­(I) complex [Fe­(PCP^CH2^-*i*Pr)­(CO)_2_] was isolated as reported previously.^8b^ The complex was fully characterized by a combination of ^1^H, ^13^C­{^1^H}, and ^31^P­{^1^H} NMR spectroscopy, IR spectroscopy, and HR-MS analysis. In the ^1^H NMR spectrum the hydride ligand appeared at −8.71 ppm as a well-resolved triplet with a coupling constant of *J* = 50.4 Hz. The ^13^C­{^1^H} NMR spectrum shows two characteristic low field triplet resonances at 217.3 ppm (*J*_*PC*_ = 16.2 Hz) and 216.7 ppm (*J*_*PC*_ = 12.3 Hz) that can be assigned to the carbonyl ligands. The *ipso* carbon was detected at 172.7 ppm (*J*_*PC*_ = 12.3 Hz). The ^31^P­{^1^H} NMR spectrum shows a singlet resonance at 109.7 ppm. In the IR spectrum, the complex exhibits two strong absorption bands at 1967 and 1935 cm^–1^ indicating a cis-arrangement of the CO ligands. The Fe–H vibration was observed at 1885 cm^–1^. The spectroscopic data are in good agreement with related PCP pincer complexes such as [Fe­(PCP^O^-*i*Pr)­(CO)_2_H] published by Guan et al.^5a^ The structure of **6** was elucidated by means of single-crystal X-ray crystallography. A molecular view is depicted in Figure 4 with selected bond lengths and angles reported in the caption.

**Figure 4:**
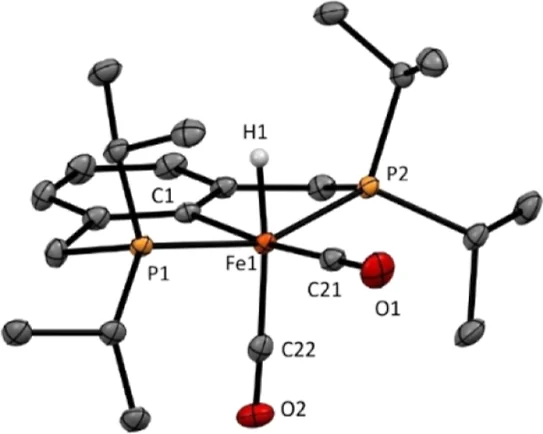
Structural view of [Fe­(PCP^CH2^-*i*Pr)­(H)­(CO)_2_] (**6**) showing 50% thermal ellipsoids (H-Atoms omitted for clarity). Selected bond-lengths (Å) and angles (°): Fe1–C1 2.023(2), Fe1–C21 1.772(2), Fe1–C22 1.823(2), Fe1–P1 2.1908(6), Fe1–P2 2.1675(6), Fe1–H1 1.41 (2), P2–Fe1–P1 156.36(2), C21–Fe1–C22 103.56(9), C21–Fe1 C1 174.57(8), C22–Fe1–H1 171 (1).

Treatment of complex **3c** with HBpin (4 equiv) for 2.5 h at room temperature afforded [Fe­(PCP^CH2^-*i*Pr)­(κ^2^ *H,H*-H_2_Bpin)­(CO)] (**7**) in 63% isolated yield (Scheme 4). This complex features a κ^2^ *H,H*-bound H_2_Bpin ligand. While **7** can be stored in the solid state under an inert atmosphere of argon at −30 °C for several weeks, in solution this compound starts to decompose within a few minutes thereby releasing free HBpin and presumably an elusive highly active 16e hydride complex [Fe­(PCP^CH2^-*i*Pr)­(H)­(CO)] which could not be detected by NMR or IR spectroscopy. This process is slowed down if an excess of HBpin (4 equiv) was added apparently preventing the dissociation of HBpin. Complex **7** was again fully characterized by ^1^H, ^11^B­{^1^H}, ^13^C­{^1^H}, and ^31^P­{^1^H} NMR spectroscopy and IR spectroscopy as well as high-resolution mass spectrometry. The IR spectrum of **7** shows a strong intensity absorption in the bridging hydride region at 2288 cm^–1^, while the CO stretching frequency is observed at 1928 cm^–1^. In the ^1^H NMR spectrum the κ^2^-bound H_2_Bpin ligand gives rise to two broad low-field resonances at −9.71 and −13.40 ppm integrated to one hydride each. In the ^13^C­{^1^H} NMR spectrum a characteristic low field triplet resonance appeared at 222.4 ppm (*J*_*PC*_ = 22.8 Hz) assignable to the carbonyl ligand. The *ipso* carbon gives rise to a triplet resonance centered at 171.0 ppm (*J*_*PC*_ = 13.2 Hz).

**Scheme 4:**
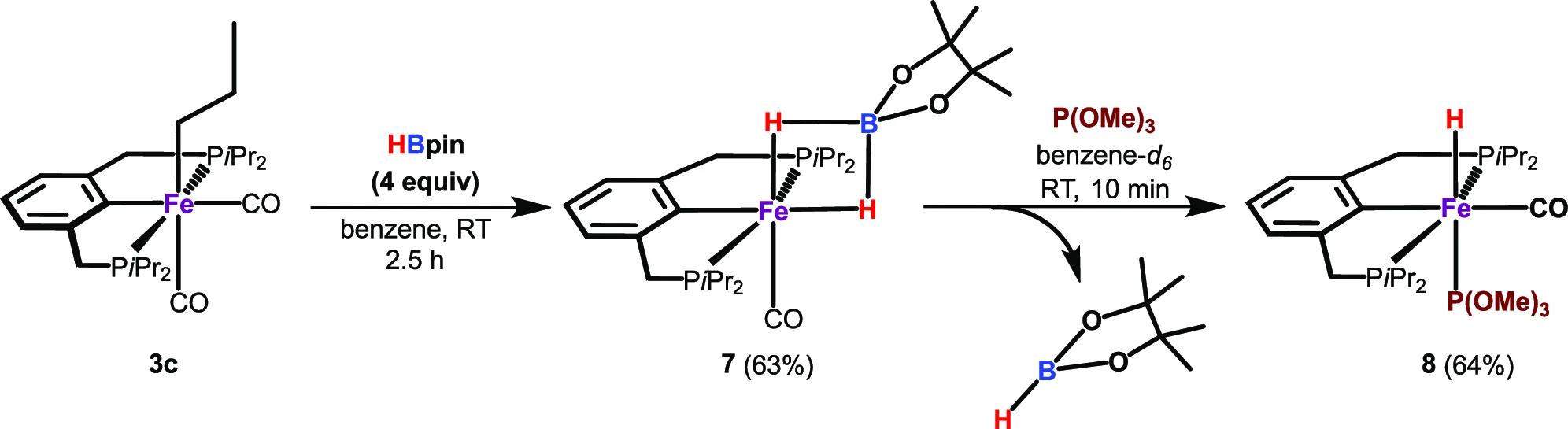
Synthesis of [Fe­(PCP^CH2^-*i*Pr)­(κ^2^ *H,H*-H_2_BPin)­(CO)] (7) and [Fe­(PCP^CH2^-*i*Pr)­(P­(OMe)_3_(CO)] (8)

In addition, the molecular of **7** structure was determined by X-ray crystallography. A structural view is presented in Figure 5 with selected bond distances and angles given in the caption. The structure proves the presence of a HBpin acting as κ^2^ *H,H*-chelate with a bite angle of 80.5(1)°. The separation between boron and the iron atom is 2.093(1). The Fe–H bond distances of are 1.54(2) and 1.50(2) Å. The metric data compare well with those found in other crystallographically characterized iron complexes containing a κ^2^ *H,H*-H_2_Bpin moiety.^11^

**Figure 5:**
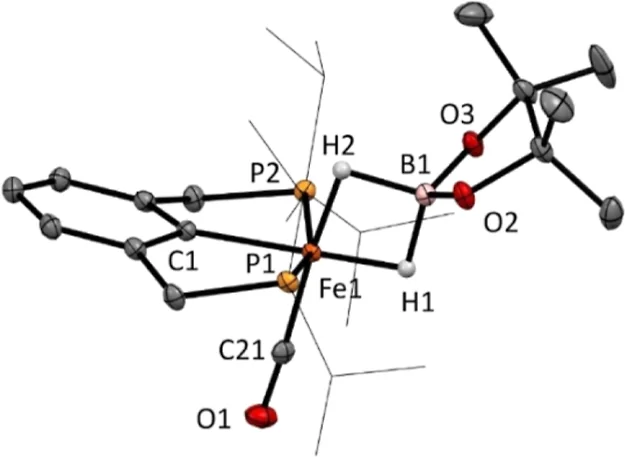
Structural view of [Fe­(PCP^CH2^-*i*Pr)­(κ^2^ *H,H*-H_2_BPin)­(CO)] (**7**) showing 50% thermal ellipsoids (H-Atoms omitted for clarity). Selected bond-lengths (Å) and angles (°): Fe1–C21 1.757(1), Fe1–C1 2.027(1) Fe1–B1 2.093(1), Fe1–P2 2.2336(3), Fe1–P1 2.2365(3), Fe1–H1 1.54(2), Fe1–H2 1.50(2), P2–Fe1–P1 161.38(1), C21–Fe1–H2 175.9(6), C1–Fe1–H1 178.8(6).

DFT calculations^12^ corroborate the κ^2^ *H,H*-H_2_Bpin coordination mode in complex **7** with equivalent Wiberg indices (WI)^13^ for both the two Fe–H bonds (0.22 and 0.24) and the two B–H bonds (0.55 and 0.57).

The dissociation of HBpin from complex **7** was studied by means of DFT calculations (Figure 6). It proceeds via an intramolecular rearrangement to intermediate **B** where the B–H bond breaking is compensated by *ipso* carbon atom of the arene ring. This process is almost thermoneutral with a barrier of 15.3 kcal/mol. Upon further rearrangement *via* intermediate **C**, the liberation of HBpin affords a coordinatively unsaturated hydride complex [Fe­(PCP^CH2^-*i*Pr)­(CO)­(H)] (**D**). Surprisingly, **D** is only less stable than the stating material [Fe­(PCP^CH2^-*i*Pr)­(κ^2^ *H,H*-H_2_BPin)­(CO)] by 5.0 kcal/mol which explains the reactivity of complex **A** (**7**) in solution.

**Figure 6:**
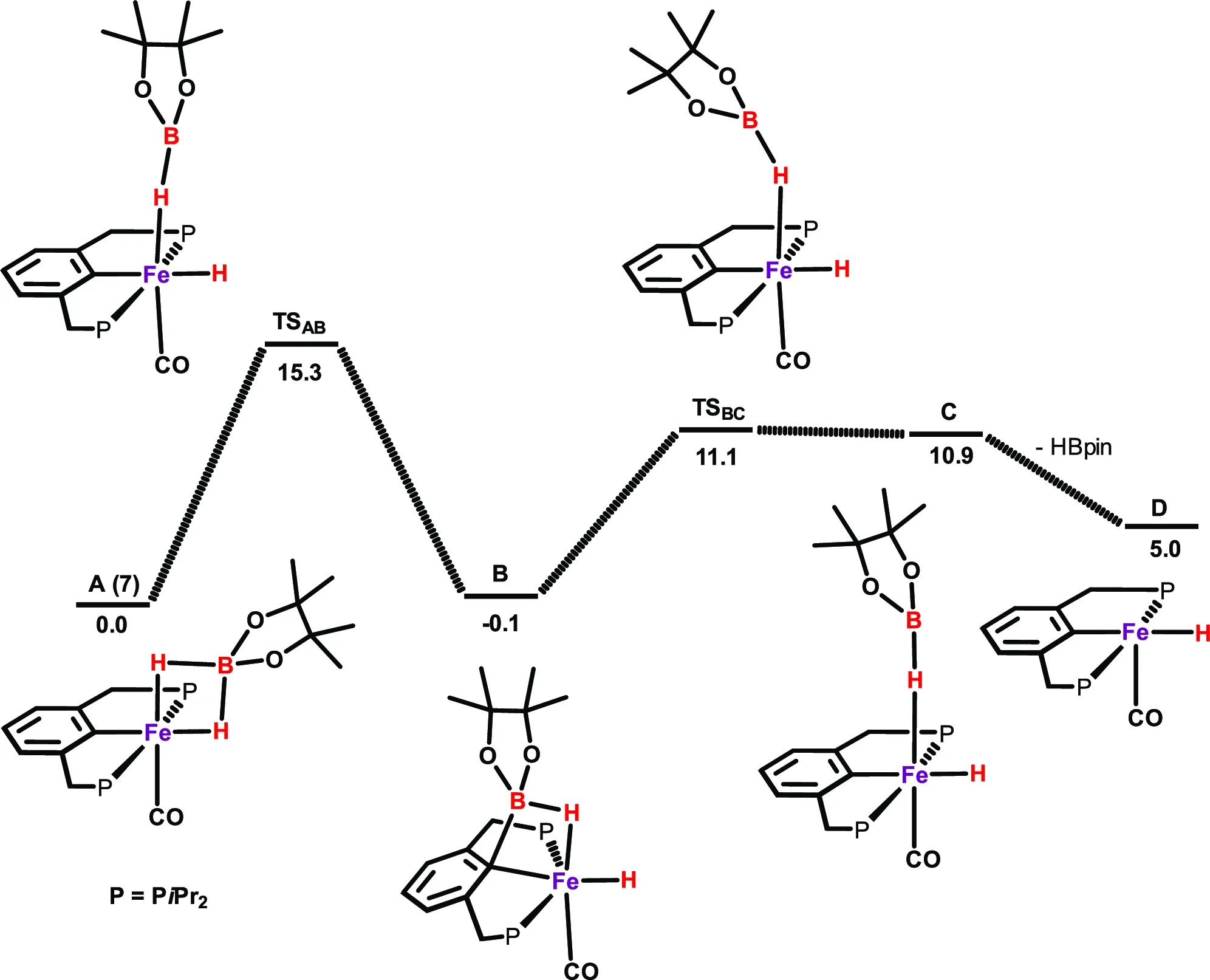
Free Energy Profile for the Dissociation of HBpin from [Fe­(PCP^CH2^-*i*Pr)­(κ^2^ *H,H*-H_2_BPin)­(CO)] (**7**). Free Energies (kcal/mol) are referred to **7** (**A** in the calculations).

The lability of HBpin in **7** is also evident from the reaction of this complex with P­(OMe)_3_ affording the hydride complex [Fe­(PCP^CH2^-*i*Pr)­(H)­(P­(OMe)_3_)­(CO)] (**8**) thereby liberating HBpin (Scheme 4). The ^1^H NMR spectrum displays a characteristic doublet of triplet hydride resonance at −10.09 ppm with a coupling constant ^*2*^ *J*_*HP*_ of 78.8 Hz. This high value is indicative that the hydride ligand is coordinated *trans* to the phosphite ligand. A structural view of **8** depicted in Figure 7 indeed confirms the *trans*-ligand arrangement.

**Figure 7:**
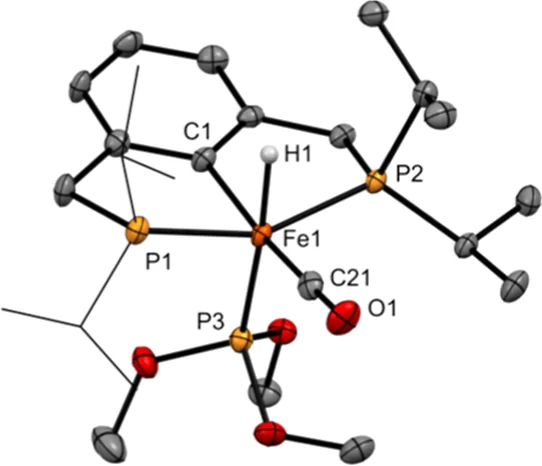
Structural view of [Fe­(PCP^CH2^-*i*Pr)­(H)­(P­(OMe)_3_)­(CO)] (**8**) showing 50% thermal ellipsoids (H-Atoms omitted for clarity). Selected bond-lengths (Å) and angles (°): Fe1–C1 2.060(4), Fe1–C21 1.750(4), Fe1–P1 2.190(1), Fe1 P2 2.202(1), Fe1–P3 2.141(1), Fe1–H1 1.44(3), P1–Fe1–P2 155.25(5) C21–Fe1–C1 171.5(2), P3–Fe1–P2 101.22(4).

Upon treatment of **3c** with 4 equiv of SiH_2_Ph_2_ the diamagnetic dark blue silyl complex [Fe­(PCP^CH2^-*i*Pr)­(SiHPh_2_)­(CO)] (**9**) was formed which, upon workup, was isolated in 51% yield (Scheme 5). With the primary and tertiary silanes SiH_3_Ph and SiHPh_3_ either a mixture of unidentified compounds was formed or no reaction took place at all. This compound was fully characterized by the means of ^1^H, ^13^C­{^1^H}, ^29^Si­{^1^H} and ^31^P­{^1^H} NMR spectroscopy and IR spectroscopy. Complex **9** displays a singlet resonance in the ^31^P­{^1^H} NMR spectrum at 83.8 ppm with observable silicon-satellites (^*2*^ *J*_*SiP*_ = 42.6 Hz). In the ^1^H NMR spectrum, the silicon bound hydrogen atom gives rise to a triplet resonance centered at 4.34 ppm (^*3*^ *J*_*HP*_ = 5.0 Hz). The ^13^C­{^1^H} NMR spectrum exhibits two characteristic triplet resonances centered at 216.2 (^*2*^ *J*_*CP*_ = 18.2 Hz) and 193.8 ppm (^*2*^ *J*_*CP*_ = 12.0 Hz) assignable to the CO ligand and the C_ipso_ carbon atom. The latter is significantly downfield shifted when compared to all other compounds described here (*cf* 178.1, 174.3, 172.7, and 171.0 ppm, respectively, for complexes **3c**, **4**, **6**, and **7**). The CO stretching frequency was detected as a strong absorption band at 1880 cm^–1^.

**Scheme 5:**
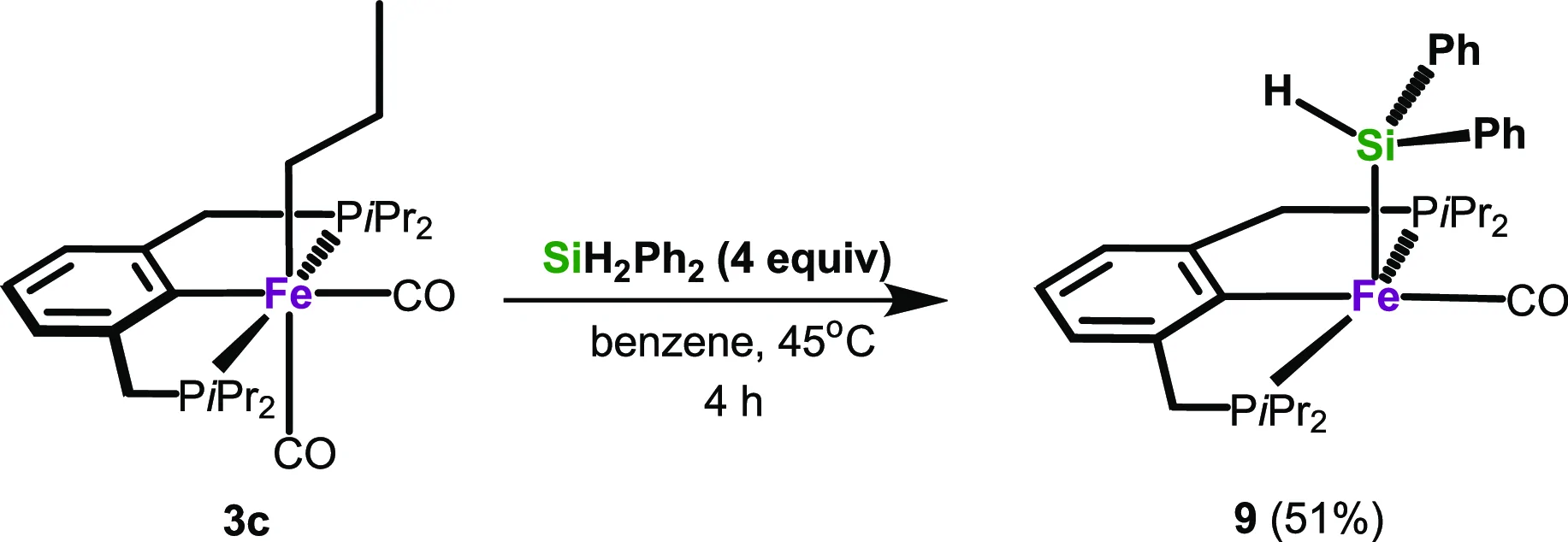
Synthesis of [Fe­(PCP^CH2^-*i*Pr)­(SiHPh_2_)­(CO)] (9)

The solid-state structure of **9** was determined by X-ray diffraction. A view of the molecular structure is depicted in Figure 8 with selected bond distances and angles reported in the caption. The complex adopts distorted square pyramidal geometry with the metal center surrounded by three donor atoms of the PCP ligand and the silyl group occupying the apical position (τ_5_ = 0.318).^14^ The C2–Fe1, Fe1–Si1, and Fe1–C1 bond distances is 1.999(2), 2.2667(5), and 1.756(2) Å, respectively. The P1–Fe1–P2, Si1–Fe1–C2 and C1–Fe1–C2 angles are 161.73(2), 87.30(5), and 178.02(8)^o^, respectively. The iron–silicon bond length is in the range of several other iron­(II)-silyl complexes.^15^ For comparison, Tonzetich et al. reported for his square planar Fe­(II) PNP pincer complexes bond lengths in the range of 2.268 to 2.384 Å depending on the coligand.^16^ An Fe­(II) CCC pincer silyl complex described by Peñas-Defrutos, García-Melchor, Fout and co-workers exhibits an Fe–Si bond distance of 2.3272(9) Å.^17^

**Figure 8:**
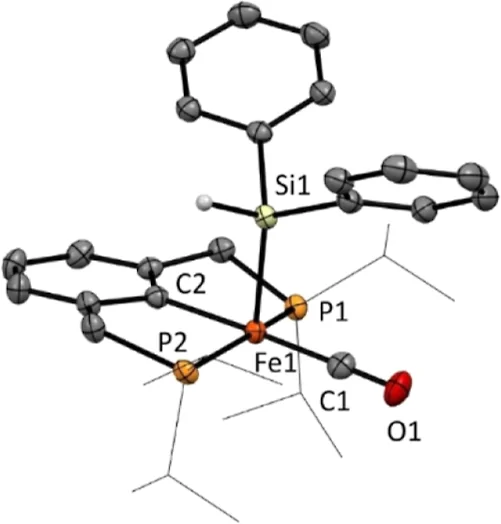
Structural view of [Fe­(PCP^CH2^-*i*Pr)­(SiHPh_2_)­(CO)] (**9**) showing 50% thermal ellipsoids (H-Atoms omitted for clarity). Selected bond-lengths (Å) and angles (°): Fe1–C1 1.756(2), Fe1–C2 1.999(2), Fe1–Si1 2.2667(5), Fe1–P1 2.2220(5), Fe1–P2 2.1943(5), C1–Fe1–C2 178.02(8), C1–Fe1–Si1 94.65(6), P1–Fe1–Si1 101.51(2), P2–Fe1–P1 161.73(2), C2–Fe1–Si1–87.30(5).

NMR monitoring of the reaction of **3c** with SiH_2_Ph_2_ revealed the formation of two diamagnetic species in an approximately 3:1 ratio (Scheme 6). In the hydride region of the ^1^H NMR spectrum (see inset of Scheme 6) a broad singlet and a sharp triplet resonance at −8.38 and −8.87 ppm (*J*_*HP*_ = 54.6 Hz), respectively, with an integral ratio of 1:1, was observed. The noncoordinated Si–H hydrogen atom exhibits a broad singlet resonance at 5.72 ppm. The ^31^P­{^1^H} NMR spectrum gives rise to signals at 105.0 and 83.8 ppm and in the ^29^Si­{^1^H} NMR spectrum signals at −1.6 and 34.9 (t, ^2^ *J*_*SiP*_ = 42.6 Hz) were detected. Accordingly, the minor species is complex **9**, while the major species appears to be a hydride complex with the silane coordinated in κ^2^ *H,Si*-fashion. Such a coordination mode was described by Chirik and co-workers in the Fe(0) PNP pincer (bis)­silane complex [Fe­(PDI^iPr^)­(κ^2^ *H,Si*-SiH_3_Ph)_2_].^18^ ^,^ ^19^ We thus tentatively assigned this complex as [Fe­(PCP^CH2^-*i*Pr)­(H)­(κ^2–^ *H,Si*-HSiHPh_2_)­(CO)] (**10**) (Scheme 6). All attempts to isolate this complex were unsuccessful due the formation of **9** upon workup which is accompanied by liberation of H_2_.

**Scheme 6:**
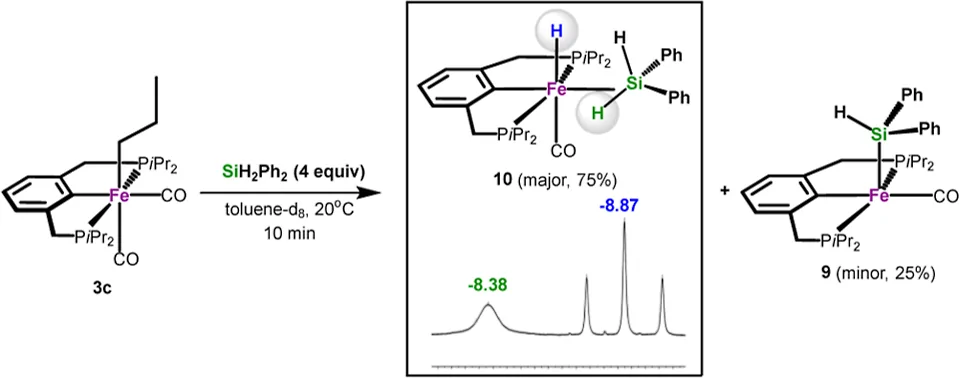
NMR Monitoring of the Reaction of 3c with SiH_2_Ph_2_ (Inset: Hydride Region of the ^1^H NMR Spectrum)

The geometry of **10** was optimized by DFT calculations (see SI), showing an apical hydride ligand *trans* to the CO and a κ^2–^ *H,Si*-HSiHPh_2_ ligand opposite to the C_ring_ atom. The relevant Wiberg indices confirm that coordination assignment with a clear stronger Fe–H bond for the hydride (WI = 0.43), compared to the Fe–(H–Si) bridging H atom (WI = 0.24). Conversely, the Si–H bond that coordinates the metal is significantly weaker than the dangling one (WI of 0.56 and 0.77, respectively) reflecting the SiH–Fe electron donation.

Further evidence for the nature of **10** was obtained from the reaction of **9** with P­(OMe)_3_ in the presence of dihydrogen (1 bar) affording also the hydride complex **8** together with free silane (Scheme 7).

**Scheme 7:**
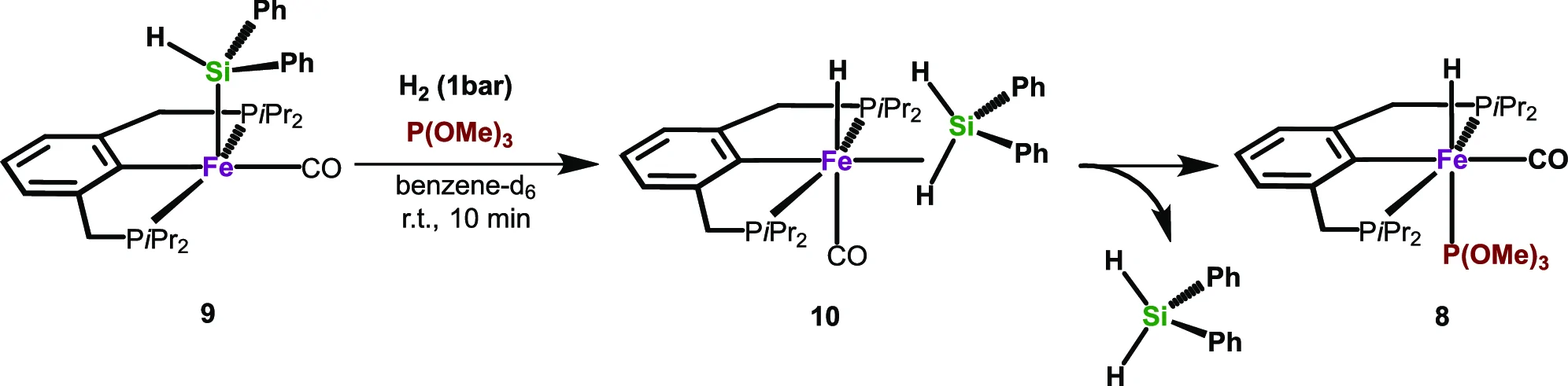
NMR Monitoring of the Reaction of 9 with P­(OMe)_3_ in the Presence of H_2_

Finally, in a preliminary study we investigated the potential of [Fe­(PCP^CH2^-*i*Pr)­(CH_2_CH_2_CH_3_)­(CO)_2_] (**3c**) as precatalyst for the hydroboration of nitriles. The reaction was performed at 60 °C in THF as solvent for 16 h and a catalyst loading of 2 mol % (Table 1). This transformation showed good functional group tolerance and a variety of aromatic substrates with functional groups such as halogenides (**CN1**, **CN2**, **CN3**, **CN6**) have been tolerated well and the corresponding products could be isolated in yields up to 91%. Amino and thioether functionalities (**CN4** and **CN5**) had no pronounced influence on the conversions and the products were isolated in 92 and 90% yields respectively. Also, aliphatic nitriles led to good conversions (**CN7** and **CN9**). Accordingly, complex **3c** presents a useful addition to other active iron catalysts capable of performing the hydroboration of nitriles.^17^ It has to be emphasized that **3c** is bench-stable even in the presence of air and does not need any additives or activation with UV-irradiation.

**Table 1:**
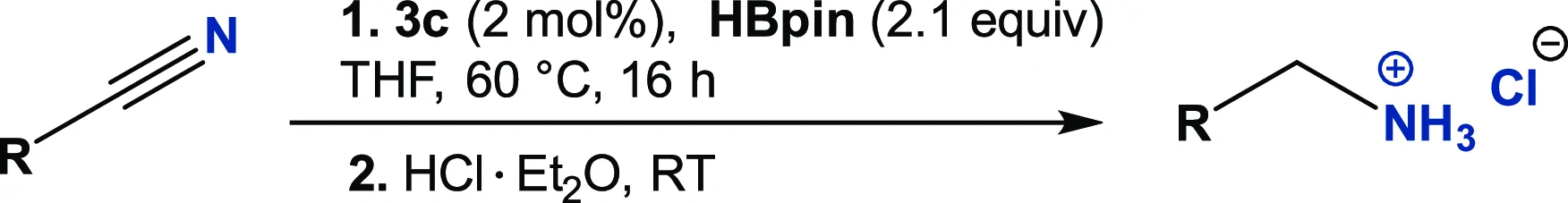

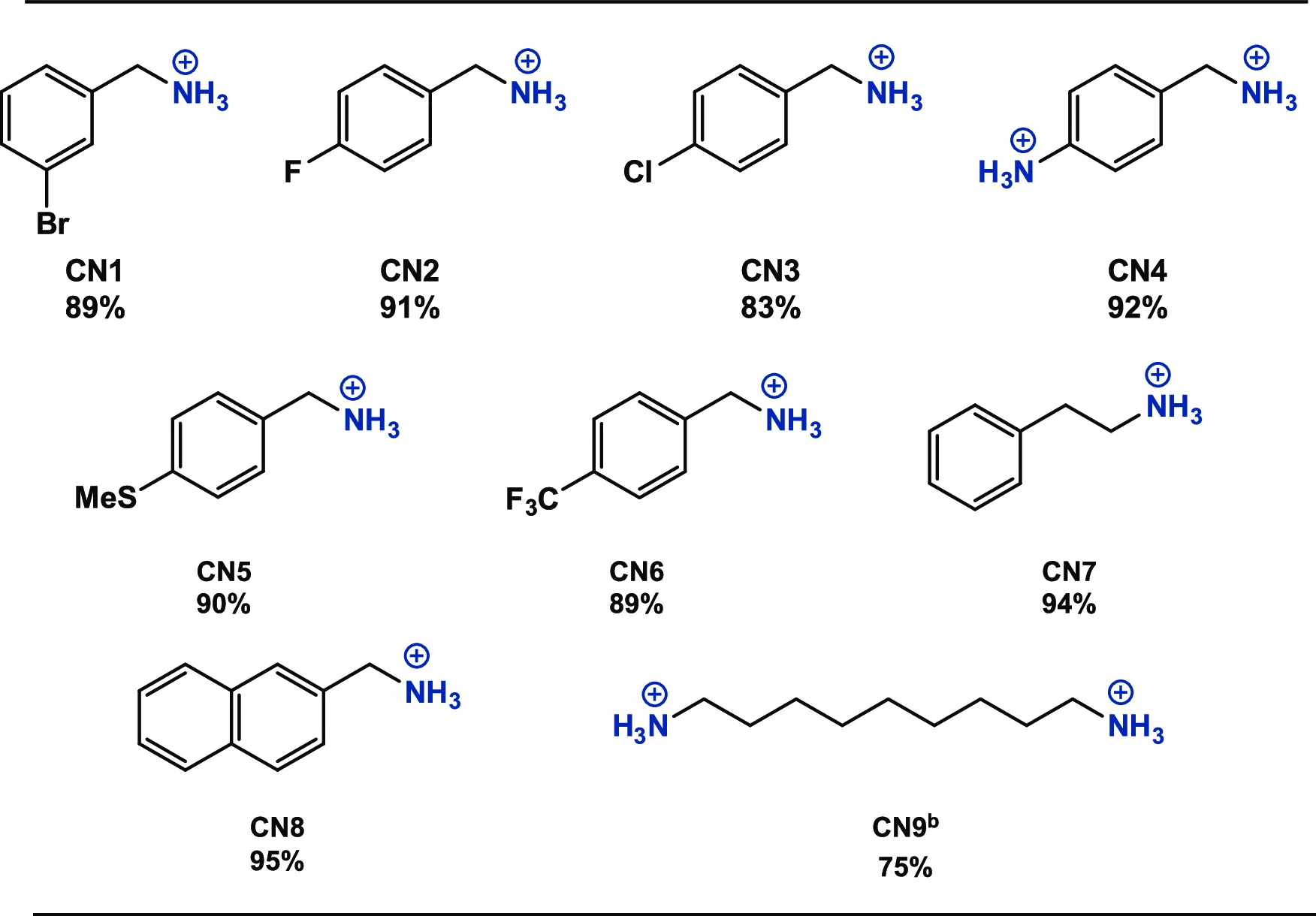
Hydroboration of Nitriles^a^

a Reaction conditions: 0.5 mmol substrate (1 equiv), **3c** (2 mol %), HBPin (2.1 equiv), THF (1 mL), 60 °C, after 16 h addition of HCl•Et_2_O (2 mL). Isolated yields as hydrochloride salts.

b HBpin (4.2 equiv).

## Conclusion

In sum, we have shown that bench stable Fe­(II) PCP pincer alkyl carbonyl complexes of the type [Fe­(PCP^CH2^-*i*Pr)­(R)­(CO)_2_)] (R = CH_3_, CH_2_CH_3_, CH_2_CH_2_CH_3_, CH_2_CH_2_CH_2_CH_3_) are readily available by the reduction of [Fe­(PCP^CH2^-*i*Pr)­(Br)­(CO)_2_] with Na/NaCl followed by oxidative addition of alkyl halides RX (X = Br or I) in high yields. Since CO ligands of alkyl carbonyl complexes are known to undergo migratory insertions to form acyl complexes if strong field ligands such as CO are present, we treated the propyl complex [Fe­(PCP^CH2^-*i*Pr)­(CO)_2_(CH_2_CH_2_CH_3_)] with CO and CN*t*Bu which afforded the acyl complexes [Fe­(PCP^CH2^-*i*Pr)­(CO)_2_(C=O)–CH_2_CH_2_CH_3_)] and [Fe­(PCP^CH2^-*i*Pr)­(CO)­(C=O)–CH_2_CH_2_CH_3_)­(CN*t*Bu)]. We could further show that if [Fe­(PCP^CH2^-*i*Pr)­(CO)_2_(CH_2_CH_2_CH_3_)] is reacted with an excess (4 equiv) of HBpin and SiH_2_Ph_2_, complexes [Fe­(PCP^CH2^-*i*Pr)­(κ^2^ *H,H*-H_2_BPin)­(CO)] and [Fe­(PCP^CH2^-*i*Pr)­(SiHPh_2_)­(CO)], respectively, are formed. The first is a very labile complex readily liberating HBpin in solution which is accompanied by decomposition to intractable materials if no potential ligands are present. This was demonstrated with P­(OMe)_3_ which reacts with [Fe­(PCP^CH2^-*i*Pr)­(κ^2^-H_2_BPin)­(CO)] to yield the hydride complex [Fe­(PCP^CH2^-*i*Pr)­(H)­(P­(OMe)_3_(CO)] and free HBpin. [Fe­(PCP^CH2^-*i*Pr)­(SiHPh_2_)­(CO)] is an unusually stable 16e complex. However, treatment with P­(OMe)_3_ in the presence of H_2_ affords also the hydride complex [Fe­(PCP^CH2^-*i*Pr)­(H)­(P­(OMe)_3_(CO)] together with free silane. Key intermediate for this reaction seems to be the hydride complex [Fe­(PCP^CH2^-*i*Pr)­(H)­(κ^2^ *H,Si*-HSiHPh_2_)­(CO)] featuring a κ^2^ *H,Si*-bound silane. This is supported by NMR spectroscopy and DFT calculations. Finally, in a preliminary catalytic reactivity study [Fe­(PCP^CH2^-*i*Pr)­(CO)_2_(CH_2_CH_2_CH_3_)] is demonstrated to be an active precatalyst for the hydroboration of nitriles with HBpin. The hydroboration reaction requires no additives and proceeds with a catalyst loading of 2 mol % at 60 °C. The reduction of nitriles and subsequent hydrolysis provide ready access to aminomethylene units in organic molecules.

## Supplementary Material





## Notes

ACS Publications Web site at DOI: Synthetic procedures, ^1^H, ^11^B­{^1^H}, ^13^C­{^1^H}, ^31^P­{H} and ^29^Si­{H} NMR spectra of all compounds and complete computational details (PDF) and corresponding references. Cartesian coordinates for DFT-optimized structures (XYZ)

## References

[1] Weber S., Kirchner K. (2022). Manganese Alkyl Carbonyl Complexes: From Iconic Stoichiometric
Textbook Reactions to Catalytic Applications. Acc. Chem. Res..

[2] c Zobernig, D. P. ; Stöger, B. ; Veiros, L. F. ; Kirchner, K. Hydrogenation of Alkenes Catalyzed by Mn(I) Alkyl Complexes Bearing NHC Phosphine Ligands ChemCatChem., 2024; Vol. 16.e202401172

[3] Andersen J.-A. M., Moss J. R. (1994). Synthesis of an Extensive Series of Manganese Pentacarbonyl
Alkyl and Acyl Compounds: Carbonylation and Decarbonylation Studies
on [Mn­(R)­(CO)_5_] and [Mn­(COR)­(CO)_5_]. Organometallics.

[4] Murugesan S., Kirchner K. (2016). Non-precious metal
complexes with an anionic PCP pincer
architecture. Dalton Trans..

[5] Bhattacharaya P., Krause J. A., Guan H. (2011). Iron Hydride
Complexes Bearing Phosphinite-Based Pincer Ligands: Synthesis, Reactivity,
and Catalytic Application in Hydrosilylation Reactions. Organometallics.

[6] Jiang S., Quintero-Duque S., Roisnel T., Dorcet V., Grellier M., Sabo-Etienne S., Darcel C., Sortais J.-B. (2016). Direct synthesis
of dicarbonyl PCP-iron hydride complexes and catalytic dehydrogenative
borylation of styrene. Dalton Trans..

[7] Dauth A., Gellrich U., Diskin-Posner Y., Ben-David Y., Milstein D. (2017). The Ferraquinone–Ferrahydroquinone Couple: Combining
Quinonic and Metal-Based Reactivity. J. Am.
Chem. Soc..

[8] Himmelbauer D., Mastalir M., Stöger B., Pignitter M., Somoza V., Veiros L. F., Kirchner K. (2018). Iron PCP Pincer
Complexes in Three Oxidation States: Reversible Ligand Protonation
to Afford an Fe(0) Complex with an Agostic C-H Arene Bond. Inorg. Chem..

[9] b Schratzberger, H. ; Kirchner, K. Hydrosilylation of Alkynes Catalyzed by an Iron­(II) PCP Pincer Alkyl Complex ChemCatChem 2025, 17, e202401398, 10.1002/cctc.202401398.

[10] Schratzberger H., Liebminger L. A., Stöger B., Veiros L. F., Kirchner K. (2023). Base Metal
Complexes bearing Pyrazole-derived PCP Ligands. Dalton Trans..

[11] Liang Q., Garcia Mayerstein H. A., Song D. (2023). Reactivity of a Piano-Stool Iron Complex toward Boranes. Organometallics.

[12] Parr, R. G. ; Yang, W. Density Functional Theory of Atoms and Molecules; Oxford University Press: New York, 1989.

[13] Wiberg K. B. (1968). Application
of the pople-santry-segal
CNDO method to the cyclopropylcarbinyl and cyclobutyl cation and to
bicyclobutane. Tetrahedron.

[14] Addison A. W., Rao T. N., Reedijk J., van Rijn J., Verschoor G. C. (1984). Synthesis,
structure, and spectroscopic properties of copper­(II) compounds containing
nitrogen-sulphur donor ligands; the crystal and molecular structure
of aqua­[1,7-bis­(N-methylbenzimidazol-2’-yl)-2,6-dithiaheptane]
copper­(II) perchlorate. J. Chem. Soc., Dalton
Trans..

[15] d Liang, Q. ; Song, D. Syntheses and Reactivity of Piano-Stool Iron Complexes of Picolyl-Functionalized N-Heterocyclic Carbene Ligands Organometallics 2021, 40, 3943–3951, 10.1021/acs.organomet.1c00515.

[16] Thompson C. V., Arman H. D., Tonzetich Z. J. (2019). Square-Planar Iron­(II) Silyl Complexes:
Synthesis, Characterization, and Insertion Reactivity. Organometallics.

[17] Najera D. C., Peñas-Defrutos M. N., García-Melchor M., Fout A. R. (2024). Identity of the Silyl Ligand in an Iron Silyl Complex
Influences Olefin Hydrogenation: An Experimental and Computational
Study Inorg. Chem.

[18] Bart S. C., Lobkovsky E., Chirik P. J. (2004). Preparation and
Molecular and Electronic
Structures of Iron(0) Dinitrogen and Silane Complexes and Their Application
to Catalytic Hydrogenation and Hydrosilation. J. Am. Chem. Soc..

[19] Itazaki M., Ito M., Nakashima S., Nakazawa H. (2016). Synthesis and characterization of [Fe­(NCCH_3_)_6_]­[*cis*-Fe­(InX_3_)_2_(CO)_4_] (X = Cl, Br, I) containing two terminal indium
fragments. Dalton Trans..

