# Supplementary material for: Synthesis, Characterization and Reactivity of Alkyl Iron(II) PCP Pincer Complexes
Source: Organometallics. 2026 Aug 28;45(17):2177–84. doi: 10.1021/acs.organomet.6c00256 (PMC13580184; doi:10.1021/acs.organomet.6c00256)

Supporting Information

Synthesis, Characterization and Reactivity of Alkyl Iron(II) PCP Pincer Complexes

Matthias Käfer,[†] Simon Gstöttner,[†] Berthold Stöger,[‡] Antoine Dupé,[$] Luis F. Veiros,[&] and Karl Kirchner*,[†]

[†] Institute of Applied Synthetic Chemistry, TU Wien, Getreidemarkt 9/163-AC, A-1060 Wien, Austria.

[‡] X-Ray Center, TU Wien, Getreidemarkt 9/163, A-1060 Wien, Austria.

[$] Institute of Chemistry, Inorganic Chemistry, University of Graz, 8010 Graz, Austria

[&] Centro de Química Estrutural, Institute of Molecular Sciences, Departamento de Engenharia Química, Instituto Superior Técnico, Universidade de Lisboa, Av. Rovisco Pais, 1049 001 Lisboa, Portugal

E-Mail Karl Kirchner: karl.kirchner@tuwien.ac.at

Table of Contents

1. Experimental Section **S2**

General Information **S2**

Synthesis **S2**

General Procedure for the Catalytic Hydroboration of Nitriles **S10**

2. X-Ray Structure Determination **S10**

3. Computational Details **S11**

4. NMR Spectra of Organic Compounds **S12**

5. References **S14**

6. NMR Spectra of Iron PCP Pincer Complexes **S18**

1. Experimental Section

General Information. All manipulations were performed under an inert atmosphere of argon by using Schlenk techniques or in an MBraun inert-gas glovebox. The solvents were purified according to standard procedures.[1] The deuterated solvents were purchased from Eurisotop SAS and dried over 4 Å molecular sieves. Complex [Fe(PCP^{CH2}-*i*Pr)(CO)$_2$Br] (**1**) was prepared according to the literature.[2]

All starting materials are known compounds and were used as obtained from commercial resources. ^{1}H, ^{11}B{^{1}H}, ^{13}C{^{1}H}, ^{31}P{^{1}H} and ^{29}Si{^{1}H} NMR spectra were recorded on Bruker AVANCE-250, AVANCE-400 and AVANCE-600 spectrometers. ^{1}H and ^{13}C{^{1}H} NMR spectra were referenced internally to residual protio-solvent and solvent resonances, respectively, and are reported relative to tetramethylsilane (δ = 0 ppm). ^{29}Si{^{1}H} NMR spectra were referenced externally to tetramethylsilane (δ = 0 ppm). ^{31}P{^{1}H} and ^{11}B NMR spectra were referenced externally to H_3PO_4 (85%) (δ = 0 ppm) and $BF_3 \cdot Et_2O$ (15% in $CDCl_3$) (δ = 0 ppm), respectively. Infrared spectra were recorded in attenuated total reflection (ATR) mode on a PerkinElmer Spectrum Two FT-IR spectrometer or on a Bruker Vertex 80 FT-IR spectrometer.

High-resolution accurate mass spectra were recorded on a hybrid Maxis Qq-aoTOF mass spectrometer (Bruker Daltonics, Bremen, Germany) fitted with an ESI source or an Agilent 6545 QTOF mass spectrometer equipped with an Agilent Dual AJS ESI ion source (Agilent Technologies, Santa Clara, CA, USA). Measured accurate mass data of the $[M]^+$ ions for confirming calculated elemental compositions were typically within ±5 ppm accuracy. The mass calibration was done with a commercial mixture of perfluorinated trialkyl-triazines (ES Tuning Mix, Agilent Technologies, Santa Clara, CA, USA). Headspace analysis were performed on a Shimadzu Nexis GC-2030 BID gas chromatograph equipped with a Balzers Prisma and a tungsten filament.

Syntheses. [Fe(PCP^{CH2}-*i*Pr)(CH$_3$)(CO)$_2$] (3a). To a solution of **1** (20 mg, 1 equiv.) in THF (2 mL) Na/NaCl (40 mg) was added. After 10 min a color-change towards green was observed which eventually darkens towards a yellow brown suspension. The mixture was stirred for 2.5 h and the resulting suspension was evaporated to dryness and thoroughly washed with *n*-pentane (5 mL total), then redissolved in THF (2 mL) and subsequently cooled to -10 °C. After that, CH_3I (11.7 µl, 0.18 mmol) was added in one portion and the resulting black mixture was allowed to reach room temperature. Then, all volatiles were removed *in vacuo* and the remaining solid was extracted with *n*-pentane (5 mL) and filtered through a syringe filter (PTFE, 0.2 µm). After evaporation of the solvent, **3a** was obtained as a light orange solid. Yield:

16.5 mg (75 %) ^{1}H NMR (600 MHz, C_6D_6, 20 °C, ppm): 7.07–7.04 (m, 3H, C*H*), 3.20 (dt, *J* = 15.9, 4.1 Hz, 2H, C*H₂*P), 3.14 (dt, *J* = 15.7, 3.8 Hz, 2H, C*H₂*P), 2.29 (ddt, *J* = 10.8, 7.2, 3.6 Hz, 2H, PC*H*(CH₃)₂), 2.13–2.05 (m, 2H, PC*H*(CH₃)₂), 1.12 (ddd, *J* = 14.6, 13.6, 7.1 Hz, 18H, PCH(C*H₃*)₂), 0.93–0.87 (m, 6H, PCH(C*H₃*)₂), -0.01 (t, *J* = 7.7 Hz, 3H, Fe-C*H₃*). ^{13}C{^{1}H} NMR (151 MHz, C_6D_6, 20 °C, ppm): 220.4 (t, *J* = 14.0 Hz, Fe-*C*O), 215.1 (t, *J* = 19.0 Hz, Fe-*C*O), 180.6 (s, *C*-Fe), 146.8 (t, *J* = 9.6 Hz, *C*-CH₂), 123.6 (s, *C*-H $^{\text{para to Fe}}$), 122.2 (t, *J* = 7.2 Hz, *C*-H), 40.1 – 39.9 (m, *C*H₂-P), 27.6 (t, *J* = 9.4 Hz, P*C*H(CH₃)₂), 24.7 (t, *J* = 8.7 Hz, P*C*H(CH₃)₂, 19.8 (s, PCH(*C*H₃)₂), 19.6 (s, PCH(*C*H₃)₂), 19.6 (s, PCH(*C*H₃)₂), 19.2 (s, PCH(*C*H₃)₂), -6.00 (t, *J* = 20.4 Hz, Fe-*C*H₃). ^{31}P{^{1}H} NMR (243 MHz, C_6D_6, 20 °C, ppm): 96.7. IR (ATR, cm^{-1}): 1969 (ν_{CO}), 1907 (ν_{CO}). HR-MS (ESI$^+$, THF) m/z calcd. for $C_{23}H_{38}FeO_2P_2$ $[M]^+$ 464.1685, found 464.1690.

[Fe(PCP^{CH2}-*i*Pr)(CH₂CH₃)(CO)₂] (3b). This complex was prepared analogously to **3a** with **1** (100 mg, 0.189 mmol) and bromoethane (84.6 µl, 1.133 mmol). **3b** was obtained as an off-white/light brown solid. Yield: 70.5 mg (78%). ^{1}H NMR (400 MHz, C_6D_6, 20 °C, ppm): 7.07–7.01 (m, 3H, C*H*), 3.26 (dt, *J* = 16.3, 4.1 Hz, 2H, C*H₂*P), 3.17 (dt, *J* = 16.3, 3.9 Hz, 2H, C*H₂*P), 2.40 (dddd, *J* = 14.4, 10.2, 7.2, 3.0 Hz, 2H, PC*H*(CH₃)₂), 2.11 (dddd, *J* = 11.3, 7.1, 5.7, 3.1 Hz, 2H, PC*H*(CH₃)₂), 1.66 (tt, *J* = 7.7, 1.2 Hz, 3H, FeCH₂C*H₃*), 1.23–1.12 (m, 12H, PCH(C*H₃*)₂), 0.91 (td, J = 7.1, 5.5 Hz, 12H, PCH(C*H₃*)₂), 0.75–0.67 (m, 2H, FeC*H₂*CH₃). ^{13}C{^{1}H} NMR (101 MHz, C_6D_6, 20 °C, ppm): 219.2 (t, *J* = 15.5 Hz, Fe*C*O), 214.1 (t, *J*= 17.7 Hz, Fe*C*O), 178.5 (t, *J* = 15.2 Hz, C_q-Fe), 147.0 (t, *J* = 10.0 Hz, C_qCH₂), 123.5 (s, *C*H), 122.1 (t, *J* = 7.3 Hz, *C*H), 40.2 (t, *J* = 14.2 Hz, *C*H₂P), 27.4 (t, *J* = 9.3 Hz, P*C*H(CH₃)₂), 24.6 (t, *J* = 8.6 Hz, P*C*H(CH₃)₂), 22.4 (s, FeCH₂*C*H₃), 20.0 (s, PCH(*C*H₃)₂), 19.8 (s, PCH(*C*H₃)₂), 19.4 PCH(*C*H₃)₂), 19.4 (s, PCH(*C*H₃)₂), 3.24 (t, *J* = 19.7 Hz, *C*H₂CH₃). ^{31}P{^{1}H} NMR (162 MHz, C_6D_6, 20 °C, ppm): 95.4. IR (ATR, cm^{-1}): 1967 (ν_{CO}), 1905 (ν_{CO}). HR-MS (ESI$^+$, THF) m/z calcd for $C_{22}H_{35}FeO_2P_2$ $[M-CH_2CH_3]^+$ 449.1450, found 449.1459.

[Fe(PCP^{CH2}-*i*Pr)(CH₂CH₂CH₃)(CO)₂] (3c). This complex was prepared analogously to **3a** with **1** (200 mg, 0.37 mmol) and 1-bromopropane (137.7 µl, 1.51 mmol). After evaporation of the solvent, **3c** was obtained as an off-white solid. Crystals suitable for X-ray diffraction analysis we obtained by cooling a saturated solution of **3c** in *n*-pentane to -30 °C. Yield: 132 mg (71 %). ^{1}H NMR (600 MHz, C_6D_6, 20 °C, ppm): 7.06–7.04 (m, 3H, C-*H*), 3.28 (dt, *J* = 16.2, 4.0 Hz, 2H, C*H₂*-P), 3.16 (dt, *J* = 16.2, 3.8 Hz, 2H, C*H₂*-P), 2.35 (pt, *J* = 7.2, 2.8 Hz, 2H, PC*H*(CH₃)₂), 2.13–2.06 (m, 2H, PC*H*(CH₃)₂), 1.94–1.87 (m, 2H, Fe-CH₂C*H₂*CH₃), 1.23–1.12 (m, 21H, PCH(C*H₃*)₂), FeCH₂CH₂CH₂C*H₃*), 0.91 (td, *J* = 7.1, 5.5 Hz, 6H, PCH(C*H₃*)₂), 0.62 (dddd, *J* = 17.7, 9.8, 5.0, 3.3 Hz, 2H, Fe-C*H₂*CH₂CH₃). ^{13}C{^{1}H} NMR (151

MHz, C_6D_6, 20 °C, ppm): 219.0 (t, *J* = 15.4 Hz, Fe-*C*O), 214.1 (t, *J* = 17.4 Hz, Fe-*C*O), 178.1 (t, *J* = 15.3 Hz, *C*-Fe), 147.0 (t, *J* = 10.0 Hz, *C*-CH_2), 123.5 (s, *C*-H $^{\text{para to Fe}}$), 122.1 (t, *J* = 7.3 Hz, *C*-H), 40.0 (t, *J* = 14.2 Hz, *C*H_2-P), 32.0 (s, Fe-CH_2*C*H_2CH_3), 27.3 (t, *J* = 9.1 Hz, P*C*H(CH_3)$_2$), 24.5 (t, *J* = 8.6 Hz, P*C*H(CH_3)$_2$), 19.9 (s, PCH(*C*H_3)$_2$), 19.7 (s, PCH(*C*H_3)$_2$), 19.5 (s, PCH(*C*H_3)$_2$), 19.4 (s, PCH(*C*H_3)$_2$), 14.3 (t, *J* = 19.5 Hz, Fe-*C*$H_2CH_2CH_3$). ^{31}P{^{1}H} NMR (162 MHz, C_6D_6, 20 °C, ppm): 95.6. IR (ATR, cm^{-1}): 1968 (ν_{CO}), 1905 (ν_{CO}). HR-MS (ESI$^+$, THF) m/z calcd for $C_{25}H_{43}FeO_2P_2$ [M+H]$^+$ 493.2088, found 493.2098.

[Fe(PCP^{CH2}-*i*Pr)($CH_2CH_2CH_2CH_3$)(CO)$_2$] (3d). This complex was prepared analogously to **3a** with **1** (40 mg, 0.075 mmol) and 1-bromobutane (20.2 µl, 0.18 mmol). After evaporation of the solvent, **3d** was obtained as an off-white solid. Yield: 26.4 mg (69 %) ^{1}H NMR (600 MHz, C_6D_6, 20 °C, ppm): 7.07–7.03 (m, 3H, C-*H*), 3.29 (dt, *J* = 16.2, 4.2 Hz, 2H, C*H$_2$*-P), 3.17 (dt, *J* = 16.2, 3.8 Hz, 2H, C*H$_2$*-P), 2.40 (pt, *J* = 7.2, 2.8 Hz, 2H, PC*H*(CH_3)$_2$), 2.09 (dtd, *J* = 11.2, 7.7, 5.6 Hz, 2H, PC*H*(CH_3)$_2$), 1.89 (dq, *J* = 12.1, 7.9 Hz, 2H, FeCH$_2$C*H$_2$*CH$_2$CH$_3$), 1.51 (hept, *J* = 7.3 Hz, 2H, FeCH$_2$CH$_2$C*H$_2$*CH$_3$), 1.17 (m, 16H, PCH(C*H$_3$*)$_2$), 1.06 (t, *J* = 7.3 Hz, 3H, FeCH$_2$CH$_2$CH$_2$C*H$_3$*), 0.92 (tt, *J* = 9.20, 3.83 Hz, 8H, PCH(C*H$_3$*)$_2$), 0.68 – 0.59 (m, 2H, FeC*H$_2$*CH$_2$CH$_2$CH$_3$). ^{13}C NMR (151 MHz, C_6D_6, 20 °C, ppm): 219.1 (t, *J* = 15.0 Hz, Fe-*C*O), 214.2 (t, *J* = 17.6 Hz, Fe-*C*O), 178.2 (t, *J* = 15.4 Hz, *C*-Fe), 147.2 (t, *J* = 10.0 Hz, *C*-CH_2), 123.7 (s, *C*-H $^{\text{para to Fe}}$), 122.3 (t, *J* = 7.3 Hz, *C*-H), 41.4 (s, FeCH$_2$*C*H$_2$CH$_2$CH$_3$), 40.3–40.0 (m, *C*H$_2$-P), 30.6 (s, FeCH$_2$CH$_2$*C*H$_2$CH$_3$), 30.2 (s, FeCH$_2$CH$_2$CH$_2$*C*H$_3$), 27.5 (t, *J* = 9.3 Hz, P*C*H(CH_3)$_2$), 24.6 (t, *J* = 9.3 Hz, P*C*H(CH_3)$_2$), 20.1 (s, PCH(*C*H$_3$)$_2$, 19.8 (s, PCH(*C*H$_3$)$_2$), 19.7 (s, PCH(*C*H$_3$)$_2$), 19.6 (s, PCH(*C*H$_3$)$_2$), 10.9 (t, *J* = 19.6 Hz, Fe*C*H$_2$CH$_2$CH$_2$CH$_3$). ^{31}P{^{1}H} NMR (162 MHz, C_6D_6, 20 °C, ppm): 95.5. IR (ATR, cm^{-1}): 1969 (ν_{CO}), 1908 (ν_{CO}). HR-MS (ESI$^+$, THF) m/z calcd. for $C_{22}H_{35}FeO_2P_2$ [M-C_4H_9]$^+$ 449.1450, found 449.1453.

[Fe(PCP^{CH2}-*i*Pr)(C(=O)-$CH_2CH_2CH_3$)(CO)$_2$] (4). A solution of **3c** (10 mg, 0.02 mmol) in benzene (1 mL) was exposed to CO gas (1 bar) for 10 min whereupon the color of the solution slightly darkened towards orange. After filtration (0.2 µm PTFE, syringe filter) and evaporation of all volatiles, **4** was obtained as light orange solid. Yield: 10.4 mg (98 %). Crystals suitable for X-ray diffraction were obtained by cooling a saturated solution of **4** in *n*-pentane to -30 °C. ^{1}H NMR (600 MHz, C_6D_6, 20 °C, ppm): 7.11 (m, 3H, C-*H*), 3.50 (dt, *J* = 15.7, 4.9 Hz, 2H, C*H$_2$*-P), 3.29 (t, *J* = 7.1 Hz, 2H, Fe-(C=O)C*H$_2$*CH$_2$CH$_3$), 3.11 (dt, *J* = 15.7, 3.3 Hz, 2H, C*H$_2$*-P), 2.07–1.95 (m, 4H, PC*H*(CH_3)$_2$, 1.71 (hept, *J* = 7.4 Hz, 2H, Fe-(C=O)CH$_2$C*H$_2$*CH$_3$), 1.24–1.16 (m, 6H, PCH(C*H$_3$*)$_2$), 1.11 (q, *J* = 7.1 Hz, 6H, PCH(C*H$_3$*)$_2$), 1.02 (q, *J* = 7.1 Hz, 6H, PCH(C*H$_3$*)$_2$), 0.97 (t, *J* = 7.4 Hz, 3H, Fe-(C=O)CH$_2$CH$_2$C*H$_3$*), 0.76–

0.69 (m, 6H, PCH(C*H*$_3$)$_2$). ^{13}C{^{1}H} NMR (151 MHz, C_6D_6, 20 °C, ppm): 266.4 (t, *J* = 25.0 Hz, Fe-(C=O)$CH_2CH_2CH_3$), 217.6 (t, *J* = 13.3 Hz, Fe-CO), 214.4 (t, *J* = 12.5 Hz, Fe-CO), 174.3 (t, J = 14.3 Hz, C-Fe), 148.0 (t, *J* = 8.7 Hz, C-CH_2), 124.3 (s, C-H $^{\text{para to Fe}}$), 122.0 (t, *J* = 7.7 Hz, C-H), 69.7 (s, Fe-(C=O)$CH_2CH_2CH_3$), 37.7 (t, *J* = 14.8 Hz, CH_2-P), 27.2 (t, *J* = 10.5 Hz, PCH(CH_3)$_2$, 26.4 (t, *J* = 9.3 Hz, PCH(CH_3)$_2$), 19.3 (s, PCH(CH_3)$_2$), 19.2(s, PCH(CH_3)$_2$), 19.1 (s, PCH(CH_3)$_2$), 18.9 (s, PCH(CH_3)$_2$), 14.4 (s, Fe-(C=O)$CH_2CH_2CH_3$). ^{31}P{^{1}H} NMR (162 MHz, C_6D_6, 20 °C, ppm): 93.1. IR (ATR, cm^{-1}): 1972 (ν_{CO}), 1911(ν_{CO}), 1615 ($\nu_{C=O,acyl}$). HR-MS (ESI$^+$, THF) m/z calcd. for $C_{26}H_{42}FeO_3P_2$ [M+Na]$^+$ 543.1850, found 543.1862.

[Fe(PCP^{CH2}-*i*Pr)(C(=O)-$CH_2CH_2CH_3$)(CO)(CN*t*Bu)] (5). Complex **3c** (10 mg, 0.02 mmol) was dissolved in benzene (1 mL) and *tert*-butyl isocyanide (8.4 µL, 0.08 mmol) was added. The solution was stirred for 10 min whereupon solution slightly darkened towards orange. After filtration (syringe filter, 0.2 µm PTFE), evaporation of all volatiles, **5** was obtained as light orange solid. Yield: 10.6 mg (91 %). Crystals suitable X-ray diffraction were obtained by cooling a saturated solution of **5** in *n*-pentane to -30 °C. ^{1}H NMR (600 MHz, C_6D_6, 20 °C, ppm): 7.14–7.06 (m, 3H, C-*H*), 3.72 (dt, *J* = 15.4, 4.8 Hz, 2H, C*H*$_2$-P), 3.46 (t, *J* = 7.2 Hz, 2H, Fe-(C=O)C*H*$_2$$CH_2CH_3$), 3.12 (dt, *J* = 15.3, 3.4 Hz, 2H, C*H*$_2$-P), 2.16 (dq, *J* = 15.5, 8.0 Hz, 4H, PC*H*(CH_3)$_2$), 1.80 (hept, *J* = 7.4 Hz, 2H, Fe-(C=O)CH_2C*H*$_2$$CH_3$), 1.31 (q, *J* = 6.8 Hz, 6H, PCH(C*H*$_3$)$_2$, 1.21 (dq, *J* = 20.7, 6.8 Hz, 12H, PCH(C*H*$_3$)$_2$), 1.06 (t, *J* = 7.5 Hz, 3H, Fe-(C=O)CH_2CH_2C*H*$_3$), 0.91 (q, *J* = 6.4 Hz, 6H, PCH(C*H*$_3$)$_2$, 0.75 (s, 9H, Fe-CN-C(C*H*$_3$)$_3$). ^{13}C{^{1}H} NMR (151 MHz, C_6D_6, 20 °C, ppm): 274.1 (t, *J* = 27.7 Hz, Fe -(C=O)$CH_2CH_2CH_3$), 220.8 (t, *J* = 13.3 Hz, Fe-CO), 181.7 (t, *J* = 16.1 Hz, C-Fe), 168.9 (t, *J* = 13.5 Hz, Fe-CN-C(CH_3)$_3$), 148.0 (t, *J* = 10.0 Hz, C-CH_2), 123.1 (s, C-H $^{\text{para to Fe}}$), 121.1 (t, *J* = 7.5 Hz, C-H), 69.9 (s, Fe-(C=O)$CH_2CH_2CH_3$), 55.6 (s, Fe-CN-C(CH_3)$_3$), 38.1 (t, *J* = 13.9 Hz, CH_2-P), 30.0 (s, Fe-(C=O)$CH_2CH_2CH_3$), 27.2–27.0 (m), PCH(CH_3)$_2$), 20.0 (s, PCH(CH_3)$_2$), 19.8 (s, PCH(CH_3)$_2$), 19.7 (s, PCH(CH_3)$_2$), 19.3 ((s, Fe-CN-C(CH_3)$_3$), 14.7 (s, Fe-(C=O)$CH_2CH_2CH_3$). ^{31}P{^{1}H} NMR (162 MHz, C_6D_6, 20 °C, ppm): 94.8. IR (ATR, cm^{-1}): 2098 (ν_{CN}), 1901 (ν_{CO}), 1591 ($\nu_{C=O}$). HR-MS (ESI$^+$, THF) m/z calcd for $C_{30}H_{50}FeNO_2P_2$ [M-H]$^+$ 574.2666, found 574.2664.

[Fe(PCP^{CH2}-*i*Pr)(H)(CO)$_2$] (6). A solution of **4** (20 mg, 0.038 mmol, 1 equiv.) in benzene (1.5 mL) was treated with HBpin (16.7 µL, 0.115 mmol) and then subsequently heated to 60 °C for 18 h. After evaporation of the solvent, extraction (*n*-pentane, 3 mL) and filtration (PTFE ,0.2µm), the remaining volatiles were evaporated to obtain **6** as a beige solid. Yield: (11.6 mg (67 %). Crystals suitable for X-ray diffraction were obtained by cooling a saturated *n*-pentane solution of **7** to -30 °C. ^{1}H NMR (400 MHz, C_6D_6, 20 °C, ppm): 7.05 – 6.96 (m, 3H,

C-$\underline{H}$), 3.18–3.10 (m, 2H, C$\underline{H}_2$-P), 2.96–2.88 (m, 2H, C$\underline{H}_2$-P), 2.08–1.97 (m, 2H, PC$\underline{H}$CH$_3$)$_2$), 1.93–1.82 (m, 2H, PC$\underline{H}$(CH$_3$)$_2$), 1.27–1.15 (m, 6H, PCH(C$\underline{H}_3$)$_2$), 1.12–1.04 (m, 12H, PCH(C$\underline{H}_3$)$_2$), 0.83 (dd, J = 13.6, 6.8 Hz, 6H, PCH(C$\underline{H}_3$)$_2$), -8.71 (t, J = 50.4 Hz, 1H, Fe-$\underline{H}$). ^{13}C{^{1}H} NMR (101 MHz, C_6D_6, 20 °C, ppm): 217.3 (t, J = 16.2 Hz, Fe-$\underline{C}$O), 216.7 (t, J = 12.3 Hz, Fe-$\underline{C}$O), 172.7 (t, J = 12.3 Hz, $\underline{C}$-Fe), 147.1 (t, J = 9.97 Hz, $\underline{C}$-CH$_2$), 123.1 (s, $\underline{C}$-H$^{\text{para to Fe}}$), 121.6 (t, J = 7.6 Hz, $\underline{C}$-H), 39.9–39.0 (m, $\underline{C}$H$_2$-P), 28.4 (t, J = 9.3 Hz, P$\underline{C}$H(CH$_3$)$_2$, 26.9 (t, J = 14.1 Hz, P$\underline{C}$H(CH$_3$)$_2$), 19.0 (s, PCH($\underline{C}$H$_3$)$_2$), 18.7 (s, PCH($\underline{C}$H$_3$)$_2$), 18.6 (s, PCH($\underline{C}$H$_3$)$_2$), 18.2 (s, PCH($\underline{C}$H$_3$)$_2$). ^{31}P{^{1}H} NMR (162 MHz, C_6D_6, 20 °C, ppm): 109.7. ATR-IR (cm^{-1}): 1967 (ν_{CO}), 1935 (ν_{CO}), 1885 ($\nu_{Fe\text{-}H}$). HR-MS (ESI$^+$, THF) m/z calcd for $C_{22}H_{35}FeO_2P_2$ [M-H]$^+$ 449.1456, found 449.1463.

[Fe(PCP^{CH2}-*i*Pr)(κ^2*H,H*-H$_2$Bpin)(CO)] (7). To a solution of **3c** (20 mg, 0.04 mmol) in benzene (1.5 mL) HBpin (23.5 µL, 0.16 mmol) was added at room temperature. The yellow solution was stirred at room temperature for 2.5 h and the solvent was removed under vacuum. The residue was extracted with Et_2O (4 mL) and filtered through a syringe filter (PTFE, 0.2 µm). After evaporation of the solvent, the product was obtained as a yellow solid, which is storable at -30 °C for weeks. Yield: 14 mg, (63%). Single crystals were obtained by cooling a saturated Et_2O solution of **7** down to -30 °C. ^{1}H NMR (600 MHz, C_6D_6, 20 °C, ppm): 7.04 (m, 3H, C$\underline{H}$), 3.19 (dt, J = 15.9, 3.3 Hz, 2H, C$\underline{H}_2$P), 3.14–3.01 (m, 2H, C$\underline{H}_2$P), 2.86–2.74 (m, 2H, PC$\underline{H}$(CH$_3$)$_2$), 2.05 (dp, J = 19.9, 7.0 Hz, 2H, PC$\underline{H}$(CH$_3$)$_2$), 1.23 (dq, J = 13.3, 7.0 Hz, 12H, PCH(C$\underline{H}_3$)$_2$), 1.16 (q, J = 6.8 Hz, 6H, PCH(C$\underline{H}_3$)$_2$), 1.08 (s, 12H, B(OC$\underline{H}_3$)$_2$), 1.06 (d, J = 7.1 Hz, 7H, PCH(C$\underline{H}_3$)$_2$, -9.71 (s, 1H, Fe-κ^2-$\underline{H}_2$Bpin), -13.40 (d, J = 95.5 Hz, 1H, Fe-κ^2-$\underline{H}_2$Bpin). ^{13}C{^{1}H} NMR (151 MHz, C_6D_6, 20 °C, ppm): 222.4 (t, J = 22.8 Hz, Fe-$\underline{C}$O), 171.0 (t, J = 13.2 Hz, $\underline{C_q}$-Fe), 149.2 (t, J = 8.5 Hz, $\underline{C}$CH$_2$), 123.4 (s, $\underline{C}$H), 121.3 (t, J = 6.4 Hz, $\underline{C}$H), 82.9 (s, $\underline{C_q}$-Bpin), 38.4–38.1 (m, $\underline{C}$H$_2$P), 27.0 (t, J = 9.9 Hz, P$\underline{C}$H(CH$_3$)$_2$), 26.1 (t, J = 8.6 Hz, P$\underline{C}$H(CH$_3$)$_2$), 24.9 (s, $\underline{C}$H$_3$-Bpin), 19.9 (s, PCH($\underline{C}$H$_3$)$_2$), 19.8 (s, PCH($\underline{C}$H$_3$)$_2$), 19.5 (s, PCH($\underline{C}$H$_3$)$_2$), 18.6 (PCH($\underline{C}$H$_3$)$_2$). ^{31}P{^{1}H} NMR (243 MHz, C_6D_6, 20 °C, ppm): 95.1. ^{11}B{^{1}H} NMR (193 MHz, C_6D_6, 20 °C, ppm): 30.9. IR (ATR, cm^{-1}): 2288 (ν_{BH}), 1928 (ν_{CO}). HR-MS (THF, 1% MeCN) m/z calcd. for $C_{21}H_{35}FeOP_2$ [M-H$_2$Bpin]$^+$ 421.1513, found 421.1511.

[Fe(PCP^{CH2}-*i*Pr)(H)(P(OMe)$_3$)(CO)] (8). To a solution of **7** (25 mg, 45.3 µmol) in C_6D_6 (0.5 mL) P(OMe)$_3$ (10.7 µL, 90.6 mmol) was added. The mixture was stirred for 5 min and then filtered into an NMR tube, sealed and monitored until **8** was fully consumed. After 4 h, all volatiles were evaporated. The residue was redissolved in *n*-pentane (0.3 mL), filtered and stored at -30 °C affording light yellow crystals. Yield: 64% (15.8 mg). ^{1}H NMR (600 MHz, C_6D_6, 20 °C, ppm): 7.01 (d, J = 7.2 Hz, 2H, C$\underline{H}$), 6.93 (t, J = 7.3 Hz, 1H, C$\underline{H}$), 3.41 (dt, J = 15.9, 3.5

Hz, 2H, $C\underline{H}_2P$), 3.21–3.15 (m, 2H, $C\underline{H}_2P$), 3.12 (d, J = 9.9 Hz, 9H, $P(OC\underline{H_3})_3$), 2.43 (dtd, J = 12.6, 10.0, 7.0 Hz, 2H, $PC\underline{H}(CH_3)_2$), 2.16 (ttd, J = 8.5, 6.7, 3.4 Hz, 2H, $PC\underline{H}(CH_3)_2$), 1.62 – 1.53 (m, 6H, $PCH(C\underline{H_3})_2$), 1.35–1.28 (m, 6H, $PCH(C\underline{H_3})_2$), 1.20 (td, J = 7.1, 4.6 Hz, 6H, $PCH(C\underline{H_3})_2$), 0.99–0.95 (m, 6H, $PCH(C\underline{H_3})_2$), -10.09 (dt, J = 78.8, 54.6 Hz, 1H, Fe-$\underline{H}$). $^{13}C\{^1H\}$ NMR (151 MHz, C_6D_6, 20 °C, ppm): 221.1 (q, J = 17.7 Hz, Fe-$\underline{C}$O), 177.7 (dt, J = 33.0, 11.8 Hz, $\underline{C_q}$-Fe), 149.3 (td, J = 11.1, 2.6 Hz, $\underline{C_q}CH_2$), 122.3 (d, J = 2.8 Hz, $\underline{C}$H), 120.2 (td, J = 8.1, 4.0 Hz, $\underline{C}$H), 51.0 (d, J = 6.4 Hz, $P(O\underline{C}H_3)_3$), 44.4 (t, J = 12.4 Hz, $\underline{C}H_2P$), 32.9 (t, J = 8.1 Hz, $P\underline{C}H(CH_3)_2$), 26.6 (td, J = 14.1, 9.7 Hz, $P\underline{C}H(CH_3)_2$), 20.9 (s, $PCH(\underline{C}H_3)_2$), 19.9 (s, $PCH(\underline{C}H_3)_2$), 18.9 (d, J = 3.2 Hz, $PCH(\underline{C}H_3)_2$), 18.7 (s, $PCH(\underline{C}H_3)_2$). $^{31}P\{^1H\}$ NMR (243 MHz, C_6D_6, 20 °C, ppm): 176.2 (t, J = 34.9 Hz, 1P, ($\underline{P}(OCH_3)_3$), 104.3 (d, J = 34.3 Hz, 2P, $\underline{P}$(iPr)$_2$). IR (ATR, cm^{-1}): 1894 (ν_{CO}). Anal. Calcd for $C_{24}H_{45}O_4P_3Fe$: C, 52.76; H, 8.30. Found: C, 52.69; H, 8.35.

[Fe(PCP^{CH2}-*i*Pr)(SiHPh$_2$)(CO)] (9). To a solution of **3c** (50 mg, 0.101 mmol) in benzene (1.5 mL) SiH_2Ph_2 (75.4 µL, 0.406 mmol) was added. The solution was stirred for 4 h at 45 °C upon which the color changed to an intense blue. The solvent was removed *in vacuo* and the residue wasre dissolved in Et_2O (4 mL), evaporated under vacuum and washed with *n*-heptane (3 x 1 mL) and the resulting dark blue powder was thoroughly dried under vacuum. Yield: 31.3 mg (51%). Crystals suitable for X-ray crystallography were obtained by cooling a saturated *n*-heptane solution of **8** to -30 °C ^1H NMR (600 MHz, C_6D_6, 20 °C, ppm): 7.48 (tt, J = 7.2, 3.8 Hz, 5H, $Si\underline{Ph_2}$), 7.36 (dd, J = 8.1, 6.4 Hz, 1H, $C\underline{H}$), 7.31 (d, J = 7.3 Hz, 2H, $C\underline{H}$), 7.07 (qd, J = 4.4,1.4 Hz, 5H, $Si\underline{Ph_2}$), 4.34 (t, J = 5.0 Hz, 1H, $Si\underline{H}$), 3.20–3.08 (m, 4H, $C\underline{H_2}P$),1.92 (dtt, J = 14.1, 10.3, 6.8 Hz, 2H, $PC\underline{H}(CH_3)_2$), 1.80 (tdd, J = 14.0, 6.9, 2.2 Hz, 2H, $PC\underline{H}(CH_3)_2$), 1.49 (q, J = 7.4 Hz, 6H, $PCH(C\underline{H_3})_2$, 0.92 (td, J = 12.4, 7.0 Hz, 12H, $PCH(C\underline{H_3})_2$), 0.25 (q, J = 7.0 Hz, 6H, $PCH(C\underline{H_3})_2$). $^{13}C\{^1H\}$ NMR (151 MHz, C_6D_6, 20 °C, ppm): 216.2 (t, J = 18.2 Hz, Fe-$\underline{C}$O), 193.8 (t, J = 12.0 Hz, $\underline{C_q}$-Fe), 155.2 (t, J = 12.0 Hz, $\underline{C_q}CH_2$), 144.1 (s, $\underline{C_q}$-HSi(Ph)$_2$), 135.4 (s, $\underline{C}$H-HSi(Ph)$_2$), 127.6 (s, $\underline{C}$H), 127.2 (s, $\underline{C}$H-HSi(Ph)$_2$), 121.9 (t, J = 8.7 Hz, $\underline{C}$H), 42.28 –41.58 (t, J = 12.25 Hz, $\underline{C}H_2P$), 27.9 (t, J = 10.9 Hz, $P\underline{C}H(CH_3)_2$), 24.0 (t, J = 6.5 Hz, $P\underline{C}H(CH_3)_2$), 22.6 (s, $PCH(\underline{C}H_3)_2$), 21.1 (s, $PCH(\underline{C}H_3)_2$), 20.1 (s, $PCH(\underline{C}H_3)_2$), 18.2 (s, $PCH(\underline{C}H_3)_2$). $^{31}P\{^1H\}$ NMR (243 MHz, C_6D_6, 20 °C, ppm): 83.8 (satellites, J_{Si-P} = 20.58 Hz). $^{29}Si\{^1H\}$-INEPT-NMR (119 MHz, C_6D_6, 20 °C, ppm): 34.9 (t, $^2J_{Si-P}$ = 42.6 Hz, magnitude of $^1J_{Si-H}$ = 167.0 Hz, $FeH\underline{Si}(Ph)_2$). IR (ATR, cm^{-1}): 1880 (ν_{CO}). Anal. Calcd for $C_{33}H_{46}P_2OSiFe$: C, 65.56; H, 7.67. Found: C, 65.43; H, 7.75.

Selected Spectroscopic Parameters for [Fe(PCP^{CH2}-*i*Pr)(H)(κ^2*H,Si*-HSiHPh$_2$)(CO)] (10). Complex **10** concomitantly forms during the synthesis of **9**. Due to sensitivity towards

vacuum which prohibited successful isolation, characteristic analytic parameters were obtained *in-situ*. ^{1}H NMR (400 MHz, toluene-d_8, 20 °C, ppm): 5.72 (br, FeH (κ^2*H,Si*-HSi-<u>*H*</u>Ph_2)), -8.38 (br s, FeH(κ^2*H,Si*-<u>*HSi*</u>HPh_2), -8.87 (t, J_{HP} = 54.65 Hz, Fe<u>*H*</u>(κ^2*H,Si*-HSiHPh_2)). $^{1}H\{^{31}P\}$ NMR (400 MHz, toluene- d_8, 20 °C, ppm): 5.72 (br, FeH(κ^{2-}*H,Si*-HSi<u>*H*</u>Ph_2)), -8.38 (br s, FeH(κ^{2-}*H,Si*-<u>*H*</u>SiHPh_2), -8.88 (br s, Fe<u>*H*</u>(κ^{2-}*H,Si*-HSiHPh_2)). $^{31}P\{^{1}H\}$ NMR (162 MHz toluene-d_8, 20 °C, ppm): 104.9.

^{1}H NMR (400 MHz, toluene-d_8, -10 °C, ppm): 5.72 (s, FeH(κ^2*H,Si*-HSi<u>*H*</u>Ph_2)), -8.36 (tdd, *J* = 8.08, 4.82, 2.45 Hz, FeH(κ^{2-}*H,Si*-<u>*H*</u>SiHPh_2), -8.74 (td, *J* = 54.60, 5.18 Hz, FeH(κ^{2-}*H,Si*-<u>*H*</u>SiHPh_2)). $^{1}H\{^{31}P\}$ NMR (400 MHz, toluene-d_8, -10 °C, ppm): 5.70 (br, magnitude of $^{1}J_{SiH}$ = 211.55 Hz (obtained from ^{29}Si-^{1}H HMBC), FeH(HSi<u>*H*</u>Ph_2)), -8.36 (s, $^{1}J_{Si-H}$ = 45.2 Hz, FeH(κ^{2-}*H,Si*-<u>*H*</u>SiHPh_2), -8.74 (br s, FeH(κ^{2-}*H,Si*-<u>*H*</u>SiHPh_2). $^{31}P\{^{1}H\}$ NMR (162 MHz, toluene-d_8, -10 °C, ppm): 105.3. ^{29}Si-^{1}H NMR (79 MHz, toluene-d_8, -10 °C, ppm): -1.6 (FeH(κ^2*H,Si*-H<u>*Si*</u>HPh_2)).

NMR Data for the Reaction of 3c with SiH_2Ph_2. To a solution of **3c** (25 mg, 0.05 mmol) in toluene-d_8 (0.5 mL) SiH_2Ph_2 (37.7 μL, 0.203 mmol) was added and the mixture was filtered into NMR tube and sealed within an argon filled glovebox. The progress of the reaction was subsequently monitored *via* NMR spectroscopy techniques.

Figure S01: ^{1}H-NMR Spectra of the progress of the reaction of **3c** with SiH_2Ph_2

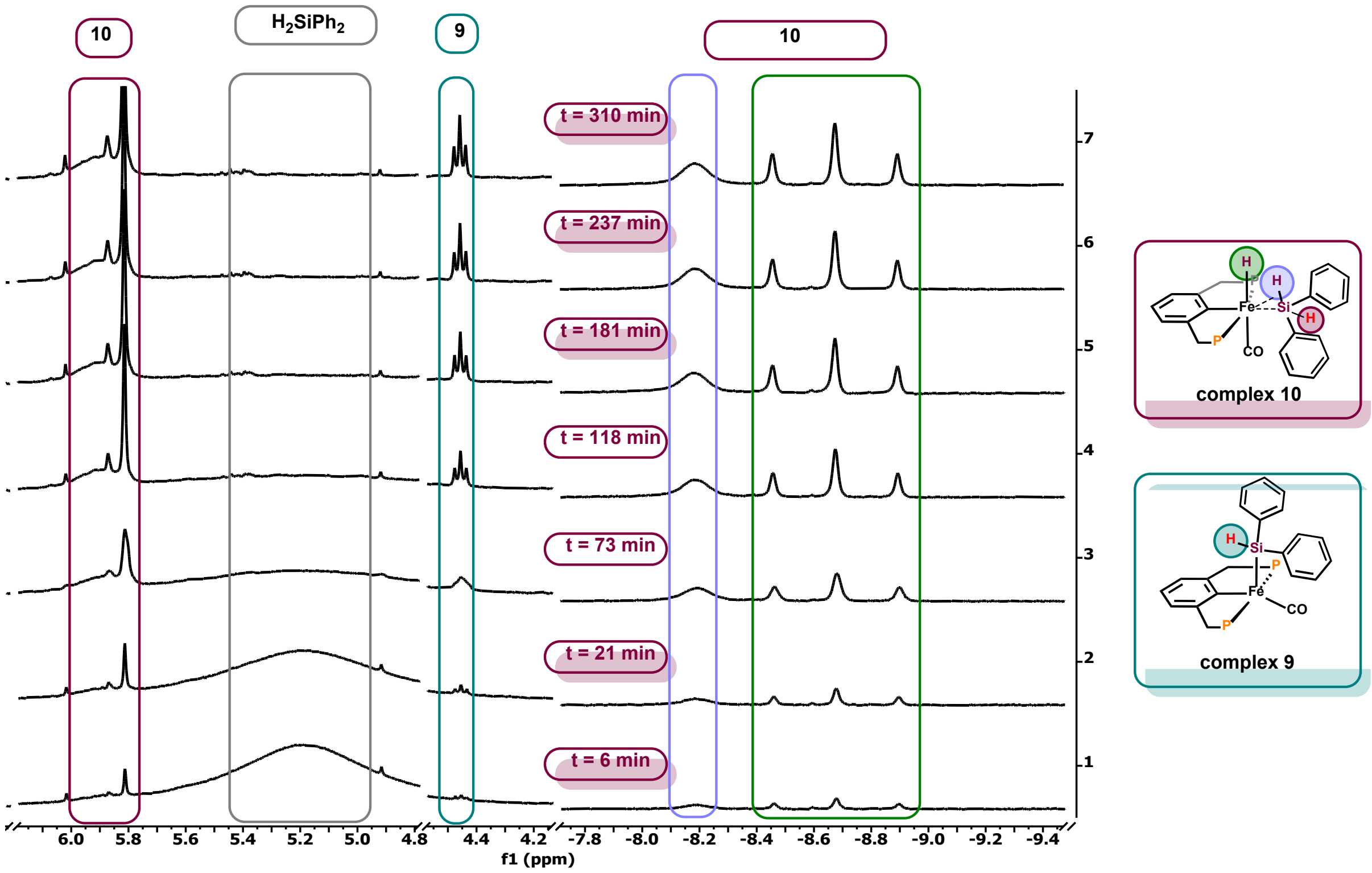

Figure S02: ^{31}P{^{1}H}-NMR Spectra of the progress of the reaction of **3c** with SiH_2Ph_2

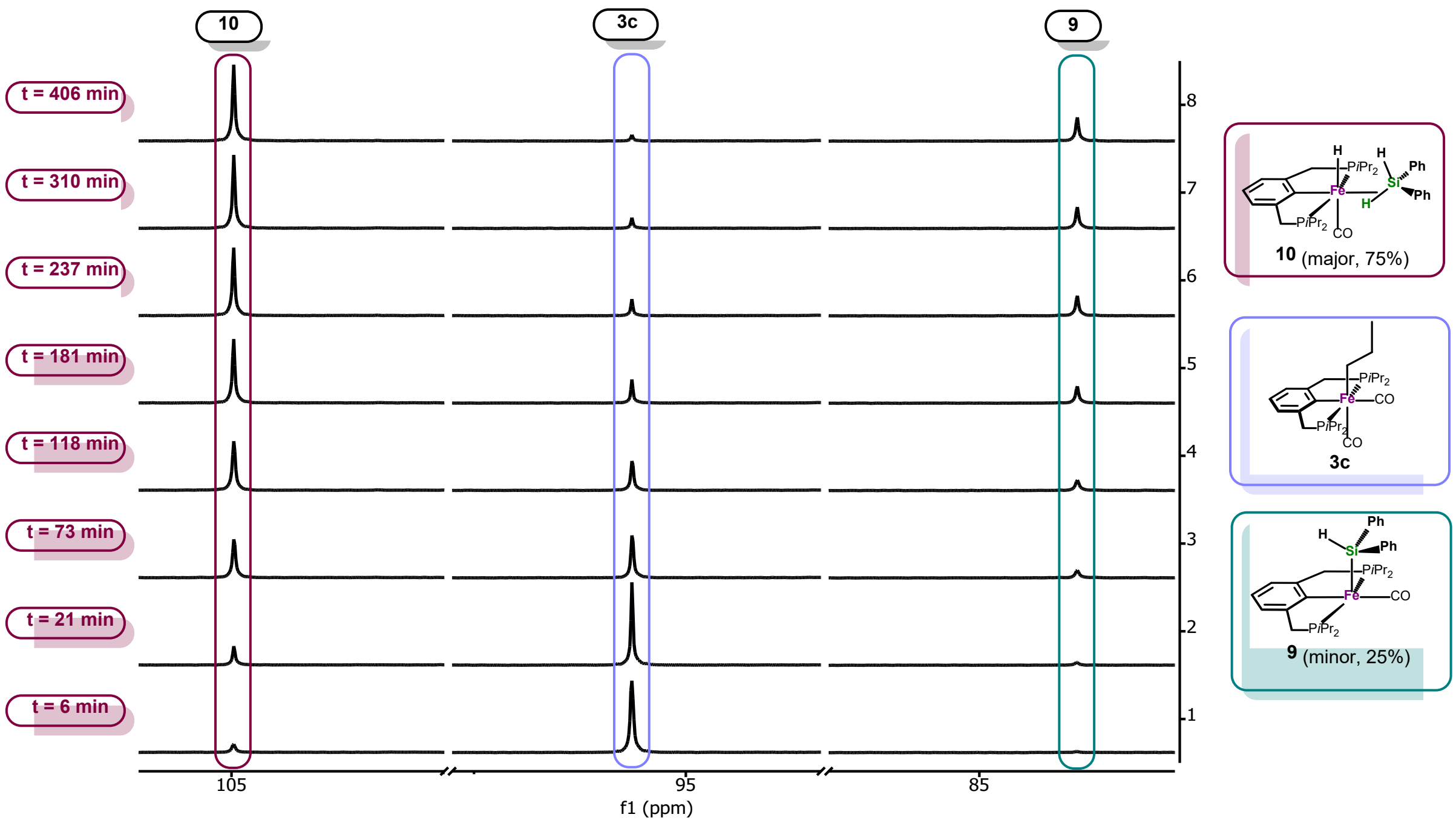

General Procedure for the Catalytic Hydroboration of Nitriles. The Nitrile substrate (0.5 mmol, 1 equiv.) was weighted into an 8 mL screw cap vial. Inside an argon filled glovebox, HBpin (1.06 mmol, 2.1 equiv.) and the catalyst **3c** (2 mol%, 5 mg) were added and the mixture was dissolved in THF (1 mL). The vial was sealed and transferred outside the glovebox and heated to 60 °C for 16 h. After cooling to room temperature, the crude mixtures were filtered through a syringe filter (PTFE 0.2 µm), and $HCl{\cdot}Et_2O$ (2 mL) was added, which resulted in the immediate precipitation of the corresponding hydrochloride salts. The suspension was stirred at room temperature for 10 min before it was put into a centrifuge for 2 min at 2000 rpm, after which the remaining solvent was decanted. The remaining solid was washed twice with Et_2O (2 mL) before it was thoroughly dried under vacuum.

2. X-Ray Structure Determination

X-ray diffraction data of **3c** (CSD 2492942) and **6** (CSD 2492944) were collected at T = 100 K in a dry stream of nitrogen on a Bruker Kappa APEX II diffractometer system using Mo-$K\alpha$ radiation (λ = 0.71073 Å). Data were reduced to intensity values with SAINT and an absorption correction was applied with the multi-scan approach implemented in SADABS.[3] **3c** was integrated as a two-component twin and refined against a HKLF5-style reflection file. X-ray diffraction data of **4** (CSD 2549421), **5** (CSD 2492943), **7** (CSD 2549422) and **8** (CSD 2573492) were collected at T = 100 K in a dry stream of nitrogen on a STOE STADIVARI diffractometer system equipped with a Dectris Eiger CdTe hybrid photon counting detector using Cu-$K\alpha$ radiation (λ = 1.54186 Å). Data were reduced with X-Area and an absorption correction was applied with the multi-scan approach implemented in LANA.[4] X-ray diffraction data of **9** (CSD 2548104) were collected at T = 100 K on an Rigaku Oxford Diffraction XtaLAB Synergy diffractometer system equipped with a HyPix-Arc 100 hybrid photon counting detector using Cu-$K\alpha$ radiation (λ = 1.54186 Å). Data were processed with CrysAlis^{Pro}.[5] All crystal structures were solved by the dual-space approach implemented in SHELXT[6] and refined against F^2 with SHELXL.[7] Non-H atoms were refined with anisotropic atomic displacement parameters. H atoms connected to C were placed in calculated positions and refined as riding on the parent atom. The hydride H in **6** was refined freely. Molecular graphics were generated with the program MERCURY.[8]

3. Computational Details

Calculations were performed using the GAUSSIAN 09 software package[9] and the PBE0 functional, without symmetry constraints. That functional uses a hybrid generalized gradient approximation (GGA), including 25 % mixture of Hartree-Fock[10] exchange with DFT[11] exchange-correlation, given by Perdew, Burke and Ernzerhof functional (PBE).[12] The optimized geometries were obtained with the Stuttgart Effective Core Potentials and associated basis set (SDD)[13] for Fe, and a standard 6-31G(d,p)[14] for the remaining elements (basis b1). Transition state optimizations were performed with the Synchronous Transit-Guided Quasi-Newton Method (STQN) developed by Schlegel *et al*,[15] following extensive searches of the Potential Energy Surface. Frequency calculations were performed to confirm the nature of the stationary points, yielding one imaginary frequency for the transition states and none for the minima. Each transition state was further confirmed by following its vibrational mode downhill on both sides and obtaining the minima presented on the energy profile. The electronic energies (E_{b1}) obtained at the PBE0/b1 level of theory were converted to free energy at 298.15 K and 1 atm (G_{b1}) by using zero-point energy and thermal energy corrections based on structural and vibration frequency data calculated at the same level.

Natural population analysis (NPA)[16] and the resulting Wiberg indices[17] were used to study the electronic structure and bonding of the optimized species. The NPA analysis was performed using the NBO 5.0 program.[18]

Single point energy calculations were performed on the geometries obtained at the PBE0/b1 level using the same functional and a 6-311++G(d,p) basis set[19] for the remaining elements (basis b2). Solvent effects (toluene) were accounted for in all calculations (including geometry optimizations) by means of the Polarisable Continuum Model (PCM) initially devised by Tomasi and coworkers[20] with radii and non-electrostatic terms of the SMD solvation model, developed by Truhler *et al.*[21] The free energy values presented (G_{b2}-D3) were corrected for dispersion by means of Grimme DFT-D3 method[22] with Becke and Johnson short distance damping,[23] being derived from the electronic energy values obtained at the PBE0-D3/b2//PBE0/b1 level (E_{b2}-D3) according to the following expression:

$$(G_{b2}\text{-D3}) = (E_{b2}\text{-D3}) + G_{b1} - E_{b1}$$

4. NMR Spectra of Organic Compounds

(3-Bromophenyl)methanamine hydrochloride (CN1)

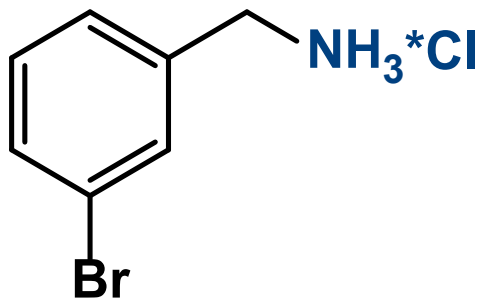

Yield: 100.5 mg (89% isolated yield), pale yellow powder. ^{1}H NMR (400 MHz, DMSO, 20°C, ppm): δ 8.50 (s, 3H, N$\underline{H}_3$Cl), 7.76 (t, J = 1.8 Hz, 1H, C$\underline{H}$), 7.58 (d, J = 7.9 Hz, 1H, C$\underline{H}$), 7.51 (d, J = 7.6 Hz, 1H, C$\underline{H}$), 7.38 (t, J = 7.9 Hz, 1H, C$\underline{H}$), 4.02 (q, J = 5.8 Hz, 2H, C$\underline{H}_2$N). ^{13}C{^{1}H} NMR (101 MHz, DMSO, 20°C, ppm): δ 136.7(s, $\underline{C}$-CH_2), 131.7 (s, $\underline{C}$-H), 131.2(s, $\underline{C}$-H), 130.7 (s, $\underline{C}$-H), 128.1 (s, $\underline{C}$-H), 121.6 (s, $\underline{C}$-Br), 41.4 (s, (s, $\underline{C}H_2$). Spectroscopic data corresponds to previously reported findings.[24]

(4-Fluorophenyl)methanamine hydrochloride (CN2)

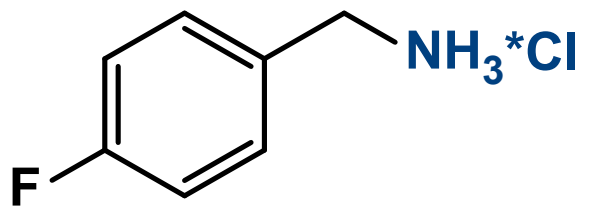

Yield: 74.6 mg (91% isolated yield), pale yellow powder. ^{1}H NMR (400 MHz, DMSO, 20°C, ppm): δ 8.36 (s, 3H, N$\underline{H}_3$Cl), 7.59 – 7.49 (m, 2H, C$\underline{H}$), 7.26 (dd, *J* = 10.1, 7.7 Hz, 2H, C$\underline{H}$), 4.01 (q, *J* = 5.8 Hz, 2H, C$\underline{H}_2$N). ^{19}F NMR (377 MHz, DMSO, 20°C, ppm): δ -113.6 (s, C$\underline{F}$). ^{13}C{^{1}H} NMR (101 MHz, DMSO, 20°C, ppm) δ 163.3 (d, *J* = 114.3 Hz, $\underline{C}$F), 131.3 (d, *J* = 7.9 Hz, $\underline{C}$H), 130.4 (s, $\underline{C}CH_2$), 115.4 (d, *J* = 22.5 Hz, $\underline{C}$H), 41.4 (s, $\underline{C}H_2NH_3Cl$). Spectroscopic data corresponds to previously reported findings.[25]

(4-Chlorophenyl)methanamine hydrochloride (CN3)

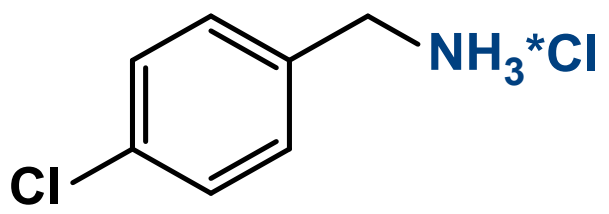

Yield: 75 mg (83% isolated yield), white powder. ^{1}H NMR (400 MHz, DMSO, 20°C, ppm): δ 8.53 (s, 3H, N$\underline{H}_3$Cl), 7.58 – 7.43 (m, 4H, C$\underline{H}$), 4.00 (q, *J* = 5.9 Hz, 2H, C$\underline{H}_2$N). ^{13}C{^{1}H} NMR (101 MHz, DMSO, 20°C, ppm): δ 133.1(s, $\underline{C}$-CH_2), 131.2 (s, $\underline{C}$Cl), 131.0 (s, $\underline{C}$H), 128.5 (s, $\underline{C}$H), 41.4 (s, $\underline{C}H_2NH_3Cl$). Spectroscopic data corresponds to previously reported findings.[26]

4-(Aminomethyl)aniline dihydrochloride (CN4)

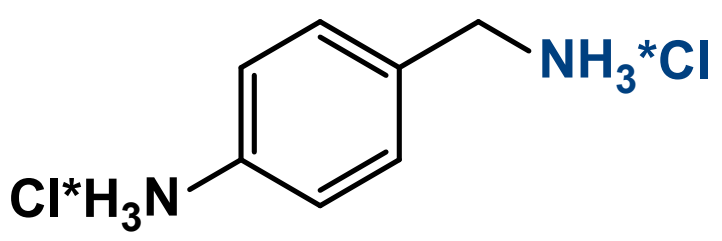

Yield: 91 mg (92% isolated yield), white powder. ^{1}H NMR (400 MHz, DMSO, 20°C, ppm): δ ^{z}8.51 (s, 3H, N$\underline{H}_3$Cl), 7.60 (d, *J* = 8.1 Hz, 2H, C$\underline{H}$), 7.40 (d, *J* = 8.4 Hz, 2H, C$\underline{H}$), 4.03 (q, *J* = 5.8 Hz, 2H, C$\underline{H}_2$N). ^{13}C{^{1}H} NMR (101 MHz, DMSO, 20°C, ppm): δ 152.8 (s, $\underline{C}NH_3Cl$), 130.8(s, $\underline{C}CH_2$), 123.7 (s, $\underline{C}$H), 121.1(s, $\underline{C}$H), 42.0 ($\underline{C}H_2N$). Spectroscopic data corresponds to previously reported findings.[27]

(4-(Methylthio)phenyl)methanamine hydrochloride (CN5)

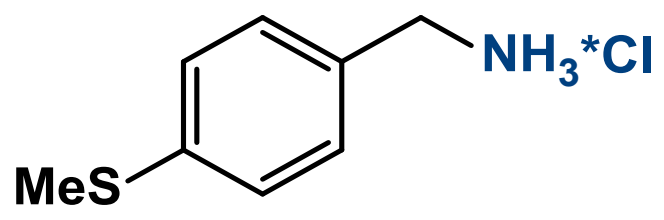

Yield: 86.7 mg (90% yield), white powder. ^{1}H NMR (400 MHz, DMSO, 20°C, ppm): δ 8.41 (s, 3H, N$\underline{H_3}$Cl), 7.43 (d, J = 8.2 Hz, 2H, C$\underline{H}$), 7.33 – 7.26 (m, 2H, C$\underline{H}$), 3.96 (q, J = 5.8 Hz, 2H, C$\underline{H_2}$N), 2.47 (s, SC$\underline{H_3}$). ^{13}C{^{1}H} NMR (101 MHz, DMSO, 20°C, ppm): δ 139.2 (s, $\underline{C}$-CH$_2$), 131.0 (s, $\underline{C}$-S), 130.1 (s, $\underline{C}$H), 126.3 (s, $\underline{C}$H) , 42.2 (s, $\underline{C}$H$_2$NH$_3$Cl), 15.2 (s, S$\underline{C}$H$_3$). Spectroscopic data corresponds to previously reported findings.[27]

(4-(Trifluoromethyl)phenyl)methanamine hydrochloride (CN6)

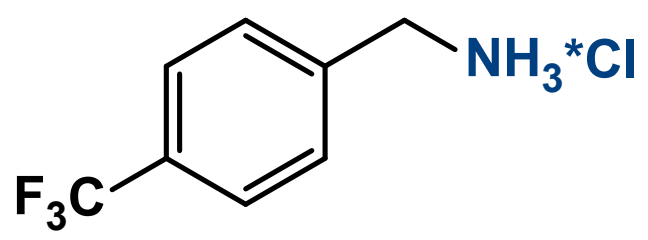

Yield: 95.6 mg (89% isolated yield), white powder. ^{1}H NMR (400 MHz, DMSO, 20°C, ppm): δ 8.75 – 8.54 (m, 3H, N$\underline{H_3}$Cl), 7.84 – 7.70 (m, 4H, C$\underline{H}$), 4.12 (q, J = 5.7 Hz, 2H, C$\underline{H_2}$N). ^{19}F NMR (377 MHz, DMSO, 20°C, ppm): δ -61.06 (s, C$\underline{F_3}$). ^{13}C{^{1}H} NMR (101 MHz, DMSO, 20°C, ppm) δ 138.8 (s, $\underline{C}$-CH$_2$), 129.6 (s, $\underline{C}$H), 128.7 (q, J = 32.2 Hz, $\underline{C}$CF$_3$), 125.4 (q, J = 3.8 Hz, $\underline{C}$H), 124.1 (q, J = 257.6 Hz, C$\underline{C}$F$_3$), 41.6 (s, $\underline{C}$H$_2$NH$_3$Cl). Spectroscopic data corresponds to previously reported findings.[25]

2-Phenylethanamine hydrochloride (CN7)

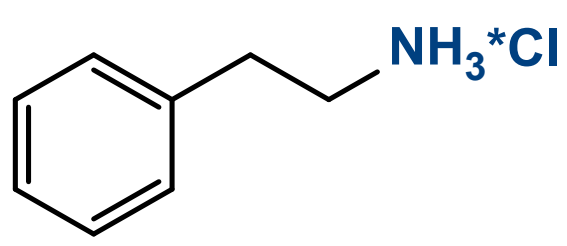

Yield: 75.2 mg (94% isolated yield), white powder. ^{1}H NMR (400 MHz, DMSO, 20°C, ppm): δ 8.10 (s, 3H, N$\underline{H_3}$Cl), 7.38 – 7.29 (m, 2H, C$\underline{H}$), 7.26 (d, J = 7.3 Hz, 3H, C$\underline{H}$), 3.01 (dp, J = 10.9, 5.8 Hz, 2H, CH$_2$C$\underline{H_2}$NH$_3$Cl), 2.89 (dd, J = 9.8, 6.1 Hz, 2H, C$\underline{H_2}$CH$_2$NH$_3$Cl). ^{13}C{^{1}H} NMR (101 MHz, DMSO, 20°C, ppm): δ 137.4 (s, $\underline{C}$-CH$_2$) , 128.6 (s, $\underline{C}$H), 128.6 (s, $\underline{C}$H), 126.7 (s, $\underline{C}$H), 39.9 (s, CH$_2$$\underline{C}H_2NH_3$Cl), 33.0 (s, $\underline{C}$H$_2$CH$_2$NH$_3$Cl). Spectroscopic data corresponds to previously reported findings.[24]

Naphthalen-2-ylmethanamine hydrochloride (CN8)

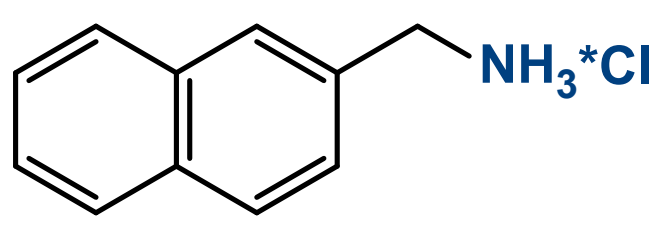

Yield: 93.4 mg (95% isolated yield), white powder. ^{1}H NMR (400 MHz, DMSO, 20°C, ppm): δ 8.51 (s, 3H, N$\underline{H_3}$Cl), 8.02 – 7.96 (m, 2H, C$\underline{H}$), 7.95 – 7.89 (m, 2H, C$\underline{H}$), 7.64 (dd, J = 8.4, 1.7 Hz, 1H, C$\underline{H}$), 7.59 – 7.54 (m, 2H, C$\underline{H}$), 4.19 (q, J = 5.8 Hz, 2H, C$\underline{H_2}$N). ^{13}C{^{1}H} NMR (101 MHz, DMSO, 20°C, ppm):δ 133.1(s, C_q), 133.1 (s, C_q), 132.1(s, , $\underline{C}$-CH$_2$) , 128.7 (s, $\underline{C}$H), 128.4 (s, $\underline{C}$H), 128.2 (s, $\underline{C}$H), 128.2 (s, $\underline{C}$H), 127.1 (s, $\underline{C}$H), 127.0 (s, $\underline{C}$H), 127.0 (s, $\underline{C}$H) 42.8 (s, $\underline{C}$H$_2$NH$_3$Cl). Spectroscopic data corresponds to previously reported findings.[25]

Nonane-1,9-diamine dihydrochloride (CN9)

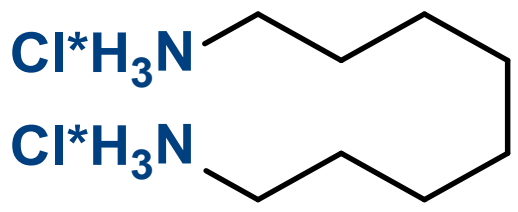

Yield: 88 mg (75% isolated yield), white powder. ^{1}H NMR (400 MHz, DMSO, 20°C, ppm): δ 7.76 (s, 6H, N$\underline{H}_3$Cl), 2.75 (q, J = 6.9 Hz, 4H, C$\underline{H}_2$N), 1.53 (h, J = 6.5 Hz, 10H, C$\underline{H}_2$), 1.34 (t, J = 10.8 Hz, 4H, C$\underline{H}_2$). ^{13}C{^{1}H} NMR (101 MHz, DMSO, 20°C, ppm): δ 38.8 (s, $\underline{C}H_2NH_3Cl$) 28.0 (s, $\underline{C}H_2$), 26.9 (s, $\underline{C}H_2$), 25.7 (s, $\underline{C}H_2$), 24.7 (s, $\underline{C}H_2$). Spectroscopic data corresponds to previously reported findings.[25]

5. References

(1) Perrin, D. D.; Armarego, W. L. F. *Purification of Laboratory Chemicals*, 3rd ed.; Pergamon: New York, **1988**.

(2) Eder, W.; Himmelbauer, D.; Stöger, B.; Veiros, L. F.; Pignitter, M.; Kirchner, K. Manganese and Iron PCP Pincer Complexes - The Influence of the Linkers on Structure and Reactivity *Dalton Trans.* **2021**, ***50***, 13915-13924.

(3) Bruker computer programs: APEX3, SAINT and SADABS (Bruker AXS Inc., Madison, WI, **2020**).

(4) X-Area 1.31.192.0, LANA 2.7.12 (STOE & Cie GmbH, Darmstadt, Germany, **2023**).

(5) CrysAlis^{Pro} 1.171.44.133a (Rigaku Oxford Diffraction, 2025).

(6) Sheldrick, G. M. Crystal structure refinement with SHELXL *Acta Crystallogr.* **2015**, *A71*, 3–8.

(7) Sheldrick, G. M. Crystal structure refinement with SHELXL *Acta Crystallogr.* **2015,** *C71*, 3–8.

(8) Macrae, C. F.; Edgington, P. R.; McCabe, P.; Pidcock, E.; Shields, G. P.; Taylor, R.; Towler, M.; van de Streek, J. Mercury: visualization and analysis of crystal structures *J. Appl. Cryst.* **2006**, *39*, 453–457.

(9) GAUSSIAN 09, Revision A.01, Frisch, M. J.; Trucks, G. W.; Schlegel, H. B.; Scuseria, G. E.; Robb, M. A.; Cheeseman, J. R.; Scalmani, G.; Barone, V.; Mennucci, B.; Petersson, G. A.; Nakatsuji, H.; Caricato, M.; Li, X.; Hratchian, H. P.; Izmaylov, A. F.; Bloino, J.; Zheng, G.; Sonnenberg, J. L.; Hada, M.; Ehara, M.; Toyota, K.; Fukuda, R.; Hasegawa, J.; Ishida, M.; Nakajima, T.; Honda, Y.; Kitao, O.; Nakai, H.; Vreven, T.; Montgomery, Jr., J. A.; Peralta, J. E.; Ogliaro, F.; Bearpark, M.; Heyd, J. J.; Brothers, E.; Kudin, K. N.; Staroverov, V. N.; Kobayashi, R.; Normand, J.; Raghavachari, K.; Rendell, A.; Burant, J. C.; Iyengar, S. S.; Tomasi, J.; Cossi, M.; Rega, N.; Millam, J. M.; Klene, M.; Knox, J. E.; Cross, J. B.; Bakken, V.; Adamo, C.; Jaramillo, J.; Gomperts, R.; Stratmann, R. E.; Yazyev, O.; Austin, A. J.; Cammi, R.; Pomelli, C.; Ochterski, J. W.; Martin, R. L.; Morokuma, K.; Zakrzewski, V. G.; Voth, G. A.; Salvador, P.; Dannenberg, J. J.; Dapprich, S.; Daniels, A. D.; Farkas, Ö.; Foresman, J. B.; Ortiz, J. V.; Cioslowski, J.; Fox, D. J. Gaussian, Inc., Wallingford CT, **2009**.

(10) Hehre, W. J.; Radom, L.; Schleyer, P. v.R.; Pople, J. A. *Ab Initio Molecular Orbital Theory*, John Wiley & Sons, NY, **1986**.

(11) Parr, R. G.; Yang, W. *Density Functional Theory of Atoms and Molecules*; Oxford University Press: New York, **1989**.

(12) (a) Perdew, J. P.; Burke, K.; Ernzerhof, M. Generalized Gradient Approximation Made Simple *Phys. Rev. Lett.* **1996**, *77*, 3865-3868; (b) Perdew, J. P.; Burke, K.; Ernzerhof, M. Generalized Gradient Approximation Made Simple *Phys. Rev. Lett.* **1997**, *78*, 1396-1396. (c) Perdew, J. P. Density-functional approximation for the correlation energy of the inhomogeneous electron gas *Phys. Rev. B* **1986**, *33*, 8822-8824.

(13) (a) Haeusermann, U.; Dolg, M.; Stoll, H.; Preuss, H.; Schwerdtfeger, P.; Pitzer, R. M. Accuracy of energy-adjusted quasirelativistic ab initio pseudopotentials *Mol. Phys.* **1993**, *78*, 1211-1224. (b) Kuechle, W.; Dolg, M.; Stoll, H.; Preuss, H. Energy-adjusted pseudopotentials for the actinides. Parameter sets and test calculations for thorium and thorium monoxide *J. Chem. Phys.* **1994**, *100*, 7535-7542. (c) Leininger, T.; Nicklass, A.; Stoll, H.; Dolg, M.; Schwerdtfeger, P. The accuracy of the pseudopotential approximation. II. A comparison of various core sizes for indium pseudopotentials in calculations for spectroscopic constants of InH, InF, and InCl *J. Chem. Phys.* **1996**, *105*, 1052-1059.

(14) (a) Ditchfield, R.; Hehre, W. J.; Pople, J. A. Self-Consistent Molecular-Orbital Methods. IX. An Extended Gaussian-Type Basis for Molecular-Orbital Studies of Organic Molecules *J. Chem. Phys.* **1971**, *54*, 724-728. (b) Hehre, W. J.; Ditchfield, R.; Pople, J. A. Self-Consistent Molecular Orbital Methods. 12. Further extensions of Gaussian-type basis sets for use in molecular-orbital studies of organic-molecules *J. Chem. Phys.* **1972**, *56*, 2257-2261. (c) Hariharan, P. C.; Pople, J. A. Accuracy of AH equilibrium geometries by single determinant molecular-orbital theory *Mol. Phys.* **1974**, *27*, 209-214. (d) Gordon, M. S. The isomers of silacyclopropane *Chem. Phys. Lett.* **1980**, *76*, 163-168. (e) Hariharan, P. C.; Pople, J. A. Influence of polarization functions on molecular-orbital hydrogenation energies *Theor. Chim. Acta* **1973**, *28*, 213-222.

(15) (a) Peng, C.; Ayala, P. Y.; Schlegel, H. B.; Frisch, M. J. Using redundant internal coordinates to optimize equilibrium geometries and transition states *J. Comp. Chem.* **1996**, *17*, 49-56. (b) Peng, C.; Schlegel, H. B. Combining Synchronous Transit and Quasi-Newton Methods for Finding Transition States *Israel J. Chem.* **1993**, *33*, 449-454.

(16) (a) Carpenter, J. E.; Weinhold, F. Analysis of the geometry of the hydroxymethyl radical by the "different hybrids for different spins" natural bond orbital procedure *J. Mol. Struct. (Theochem)*, **1988,** *169*, 41-62. (b) Carpenter, J. E. Extension of Lewis structure concepts to open-shell and excited-state molecular species *PhD. Thesis*. University of Wisconsin, Madison, WI, **1987**. (c) Foster, J. P.; Weinhold, F. Natural hybrid orbitals *J. Am. Chem. Soc.*, **1980,** *102*, 7211-7218. (d) Reed, A. E.; Weinhold, F. Natural bond orbital analysis of near-Hartree-Fock water dimer *J. Chem. Phys.*, **1983,** *78*, 4066-4073. (e) Reed, A. E.; Weinhold, F. Natural localized molecular orbitals *J. Chem. Phys.*, **1985,** *83*, 1736-1740. (f) Reed, A. E.; Weinstock, R. B.; Weinhold, F. Natural population analysis *J. Chem. Phys.*, **1985,** *83*, 735-746. (g) Reed, A. E.; Curtiss, L. A.; Weinhold, F. Intermolecular interactions from a natural bond orbital, donor-acceptor viewpoint *Chem. Rev.*, **1988,** *88*, 899-926. (h) Weinhold, F.; Carpenter, J. E. *The Structure of Small Molecules and Ions*. Plenum, New York, **1988**; p 227.

(17) (a) Wiberg, K. B. Application of the pople-santry-segal CNDO method to the cyclopropylcarbinyl and cyclobutyl cation and to bicyclobutane *Tetrahedron* **1968**, *24*, 1083-1096. (b) Wiberg indices are electronic parameters related with the electron density in between two atoms, which scale as bond strength indicators. They can be obtained from a Natural Population Analysis.

(18) *NBO 5.0*. Glendening, E. D.; Badenhoop, J. K.; Reed, A. E.; Carpenter, J. E.; Bohmann, J. A.; Morales, C. M.; Weinhold, F.; Theoretical Chemistry Institute, University of Wisconsin, Madison, 2001.

(19) (a) McClean, A. D.; Chandler, G. S. Contracted Gaussian basis sets for molecular calculations. I. Second row atoms, *Z*=11-18 *J. Chem. Phys.* **1980**, *72*, 5639-5648. (b) Krishnan, R.; Binkley, J. S.; Seeger, R.; Pople, J. A. Self-consistent molecular orbital methods. XX. A basis set for correlated wave functions *J. Chem. Phys.* **1980**, *72*, 650-654. (c) Wachters, A. J. H. Gaussian Basis Set for Molecular Wavefunctions Containing Third-Row Atoms *J. Chem. Phys.* **1970**, *52*, 1033-1036. (d) Hay, P. J. Gaussian basis sets for molecular calculations - representation of 3D orbitals in transition-metal atoms *J. Chem. Phys.* **1977**, *66*, 4377-4384. (e) Raghavachari, K.; Trucks, G. W. Highly correlated systems: Excitation energies of first row transition metals Sc-Cu *J. Chem. Phys.* **1989**, *91*, 1062-1065. (f) Binning Jr., R. C.; Curtiss, L. A. Compact contracted basis-sets for 3rd-row atoms - Ga-Kr *J. Comp. Chem.* **1990**, *11*, 1206-1216. (g) McGrath, M. P.; Radom, L. Extension of Gaussian-1 (G1) theory to bromine-containing molecules *J. Chem. Phys.* **1991**, *94*, 511-516. (h) Curtiss, L. A.; McGrath, M. P.; Blaudeau, J.-P.; Davis, N. E.; Binning Jr., R. C.; Radom, L. Extension of Gaussian-2 theory to molecules containing third-row atoms Ga-Kr *J. Chem. Phys.*, **1995**, *103*, 6104-6113. (i) Clark, T.; Chandrasekhar, J.; Spitznagel, G. W.; Schleyer, P. v. R. Efficient diffuse function-augmented basis-sets for anion calculations. 3. The 3-21+G basis set for 1st-row elements, Li-F *J. Comp. Chem.* **1983**, *4*, 294-301. (j) Frisch, M. J.; Pople, J. A.; Binkley, J. S. Self-Consistent Molecular Orbital Methods. 25. Supplementary Functions for Gaussian Basis Sets *J. Chem. Phys.* **1984**, *80*, 3265-3269.

(20) (a) Cancès, M. T.; Mennucci, B.; Tomasi, J. A new integral equation formalism for the polarizable continuum model: Theoretical background and applications to isotropic and anistropic dielectrics *J. Chem. Phys.* **1997**, *107*, 3032-3041. (b) Cossi, M.; Barone, V.; Mennucci, B.; Tomasi, J. Ab initio study of ionic solutions by a polarizable continuum dielectric model *Chem. Phys. Lett.* **1998**, *286*, 253-260. (c) Mennucci, B.; Tomasi, J. Continuum solvation models: A new approach to the problem of solute's charge distribution and cavity boundaries *J. Chem. Phys.* **1997**, *106*, 5151-5158. (d) Tomasi, J.; Mennucci, B.; Cammi, R. Quantum mechanical continuum solvation models *Chem. Rev.* **2005**, *105*, 2999-3094.

(21) Marenich, A. V.; Cramer, C. J.; Truhlar, D. G. Universal solvation model based on solute electron density and a continuum model of the solvent defined by the bulk dielectric constant and atomic surface tensions *J. Phys. Chem. B*, **2009**, *113*, 6378-6396.

(22) Grimme, S.; Antony, J.; Ehrlich, S.; Krieg, H. A consistent and accurate ab initio parameterization of density functional dispersion correction (DFT-D) for the 94 elements H-Pu *J. Chem. Phys.* **2010**, *132*, 154104.

(23) (a) Becke, A. D.; Johnson, E. R. A density-functional model of the dispersion interaction *J. Chem. Phys.* **2005**, *122*, 154101. (b) Johnson, E. R.; Becke, A. D. A post-Hartree-Fock model of intermolecular interactions *J. Chem. Phys.* **2005**, *123*, 24101. (c) Johnson, E. R.; Becke, A. D. A post-Hartree-Fock model of intermolecular interactions: Inclusion of higher-order corrections *J. Chem. Phys.* **2006**, *124*, 174104.

(24) Sanagawa, A.; Nagashima, H. Hydrosilane Reduction of Nitriles to Primary Amines by Cobalt–Isocyanide Catalysts *Org. Lett.* **2019**, *21*, 1, 287–291.

(25) Chen, F.; Topf, C.; Radnik, J.; Kreyenschulte, C.; Lu, H.; Schneider, M.; Surkus, A.-E.; He, L.; Junge, K.; Beller, M. Stable and Inert Cobalt Catalysts for Highly Selective and Practical Hydrogenation of C≡N and C=O Bonds *J. Am. Chem. Soc* **2016***, 138*, 8781-8788.

(26) Neumann, J.; Bornschein, C.; Jiao, H.; Junge, K.; Beller, M. Hydrogenation of Aliphatic and Aromatic Nitriles Using a Defined Ruthenium PNP Pincer Catalyst *Eur. J. Org. Chem.* **2015**, 5944-5948.

(27) Adam, R.; Bheeter, C. B.; Jackstell, R.; Beller, M. A Mild and Base-Free Protocol for the Ruthenium-Catalyzed Hydrogenation of Aliphatic and Aromatic Nitriles with Tridentate Phosphine Ligands *ChemCatChem* **2016**, *8*, 1329-1334.

5. NMR Spectra of Iron PCP Pincer Complexes

Figure S03. ^{1}H-NMR (C_6D_6) spectrum of [Fe(PCP^{CH2}-*i*Pr)(CH_3)(CO)$_2$] **(3a)**

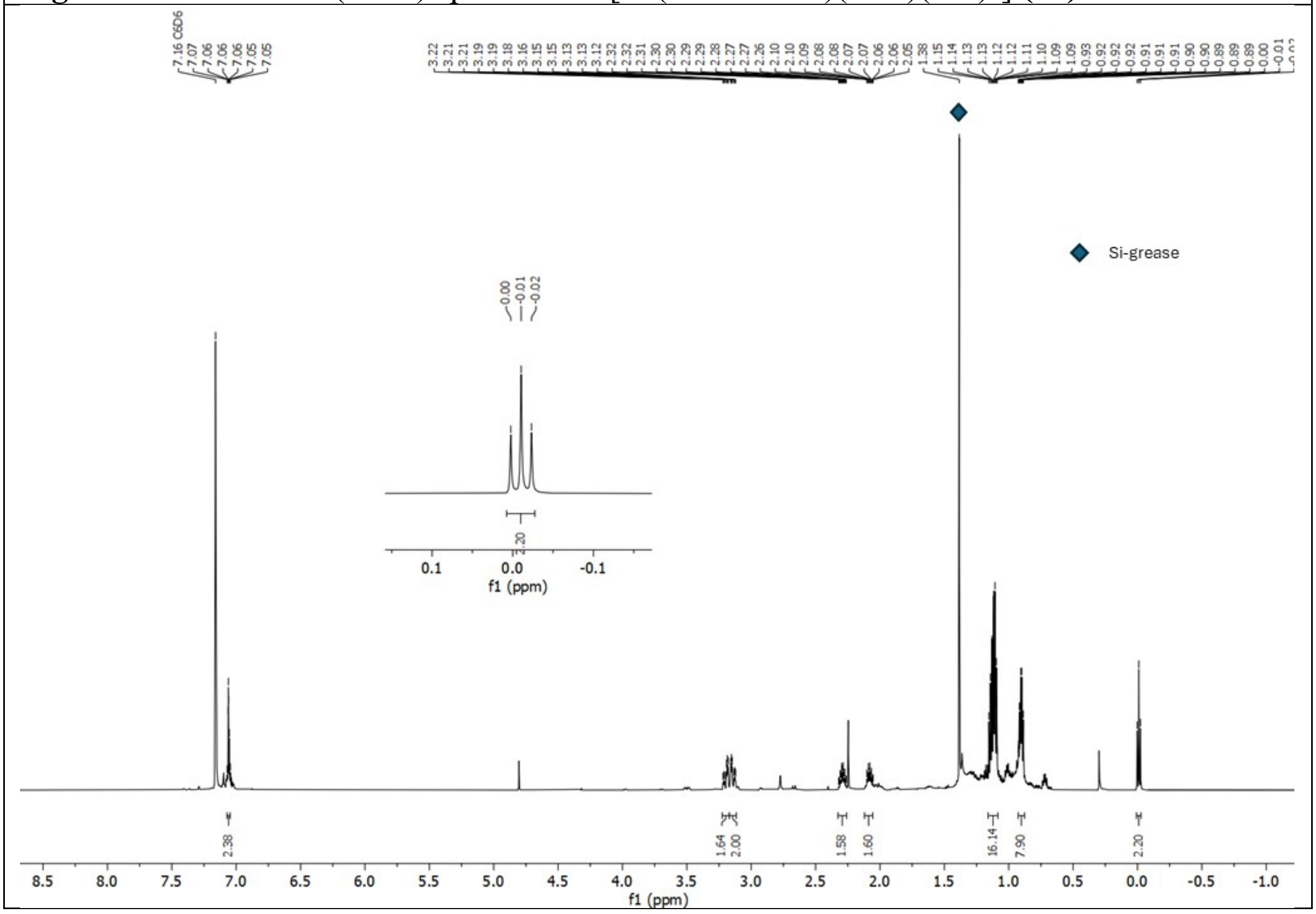

Figure S04. ^{13}C{^{1}H}-NMR (C_6D_6) spectrum of [Fe(PCP^{CH2}-*i*Pr)(CH_3)(CO)$_2$] **(3a)**

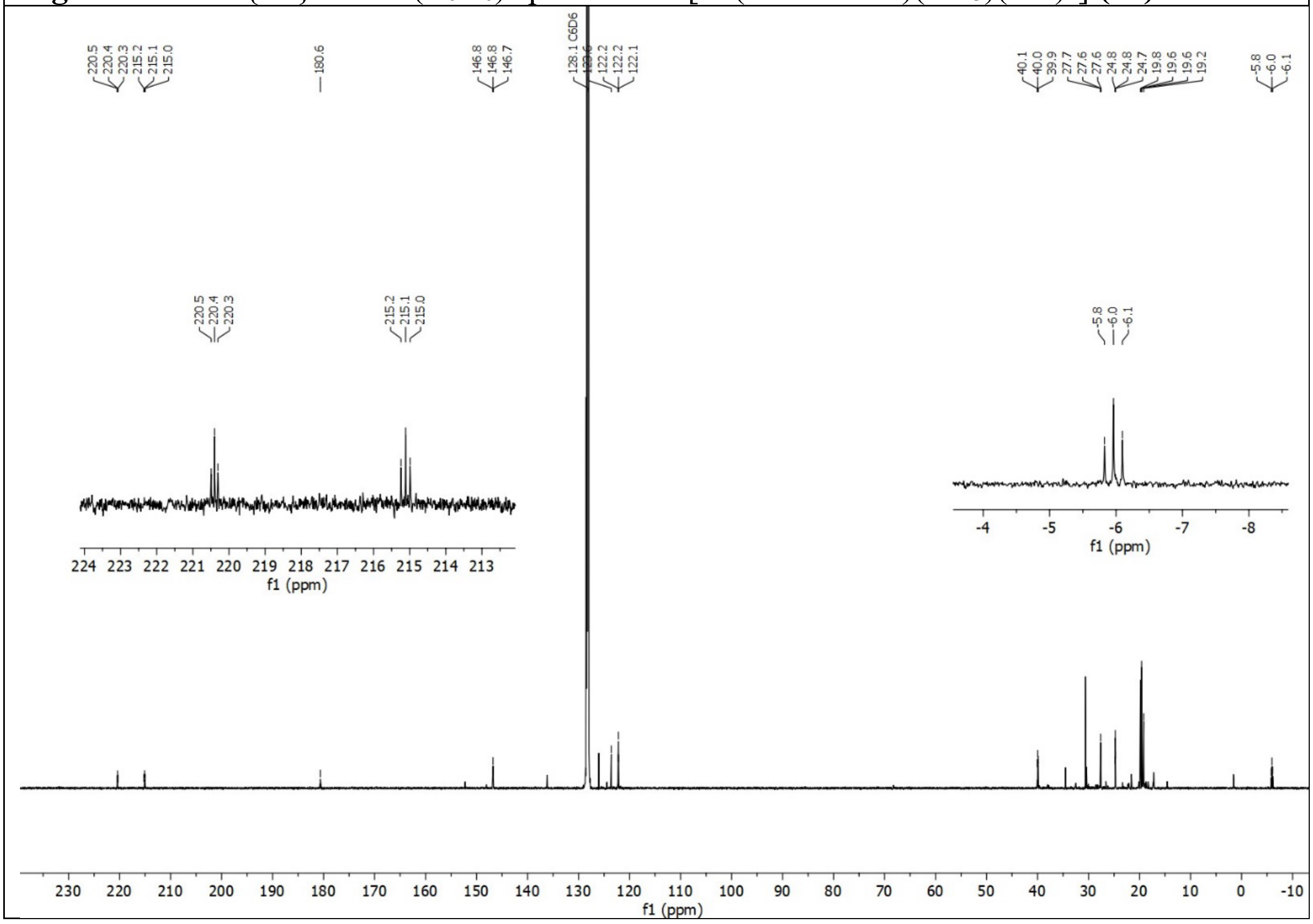

Figure S05. $^{31}P\{^{1}H\}$-NMR (C_6D_6) spectrum of [Fe(PCP^{CH2}-*i*Pr)(CH_3)$(CO)_2$] **(3a)**

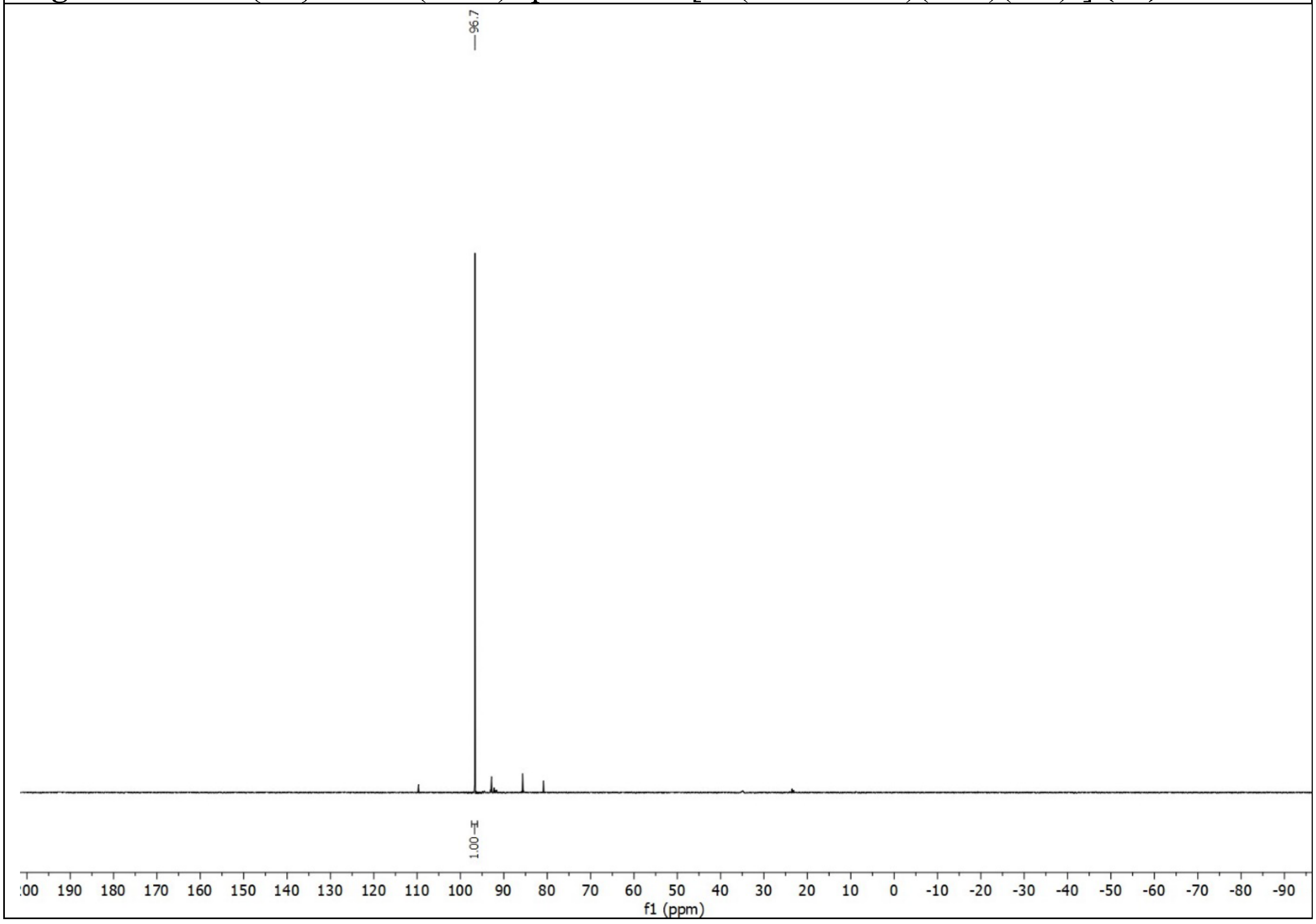

Figure S06. ^{1}H-NMR (C_6D_6) spectrum of [Fe(PCP^{CH2}-*i*Pr)(CH_2CH_3)$(CO)_2$] **(3b)**

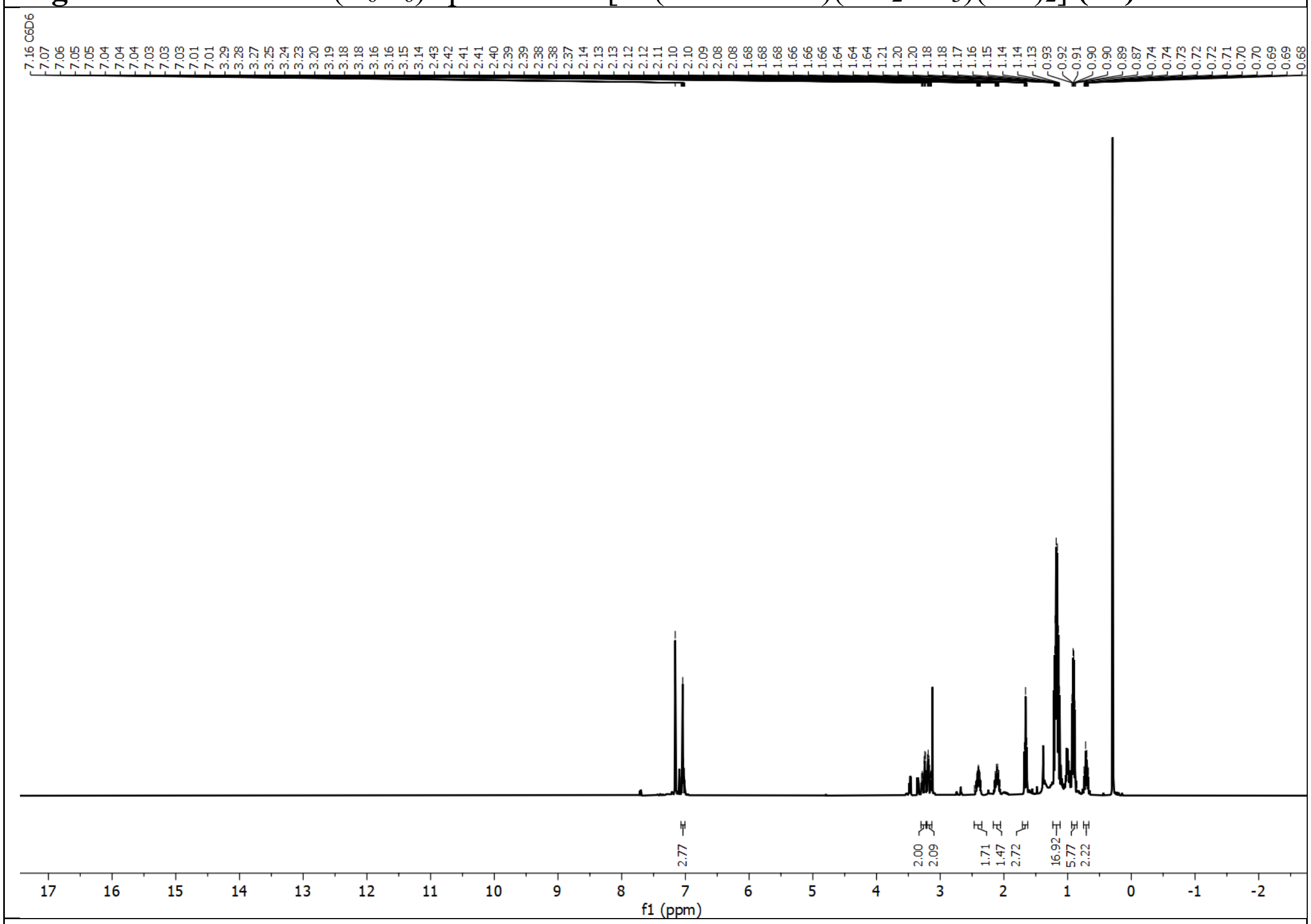

Figure S07. $^{13}C\{^{1}H\}$-NMR (C_6D_6) spectrum of [Fe(PCP^{CH2}-*i*Pr)(CH_2CH_3)$(CO)_2$] **(3b)**

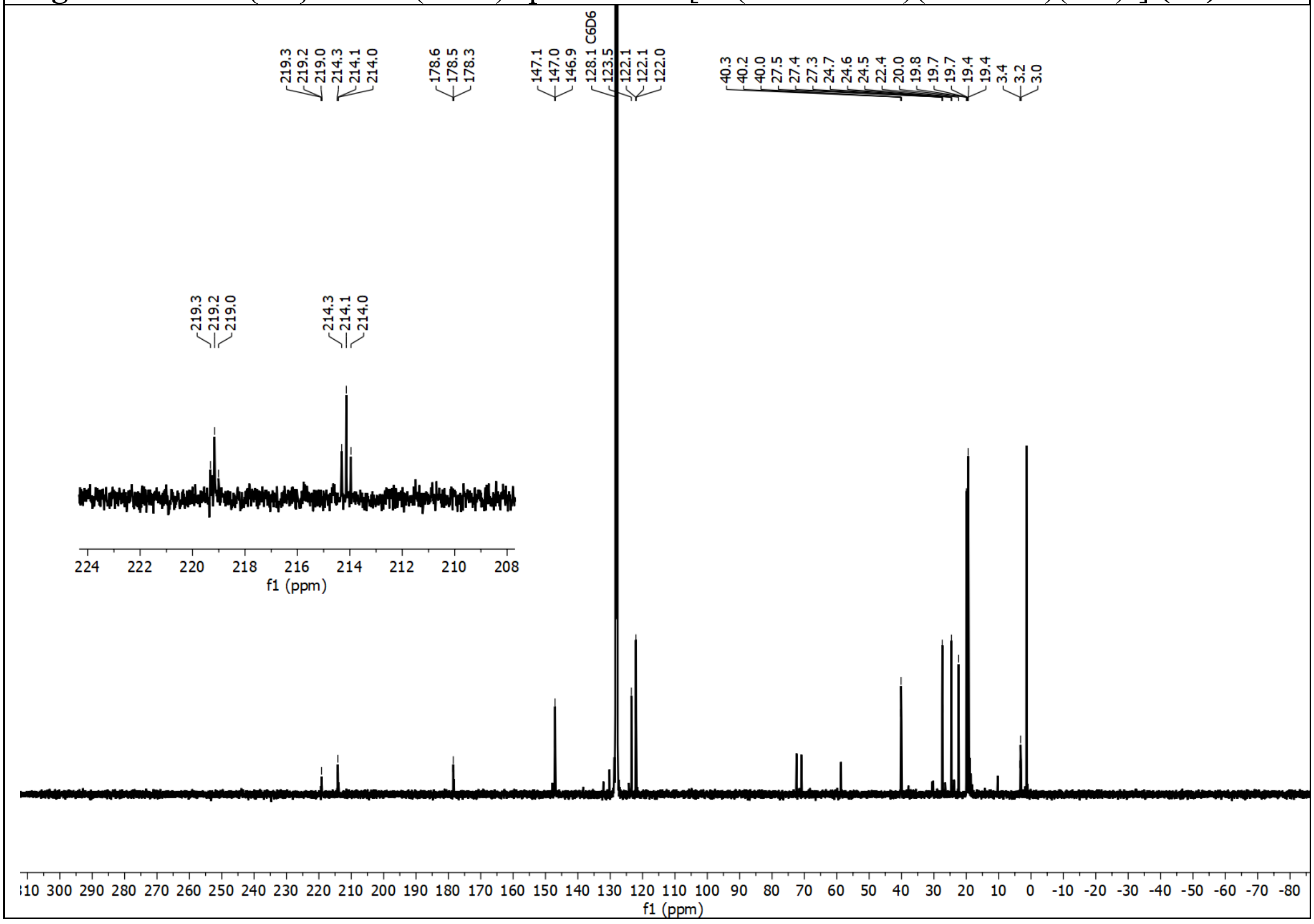

Figure S08. $^{31}P\ \{^{1}H\}$-NMR (C_6D_6) spectrum of [Fe(PCP^{CH2}-*i*Pr)(CH_2CH_3)$(CO)_2$] **(3b)**

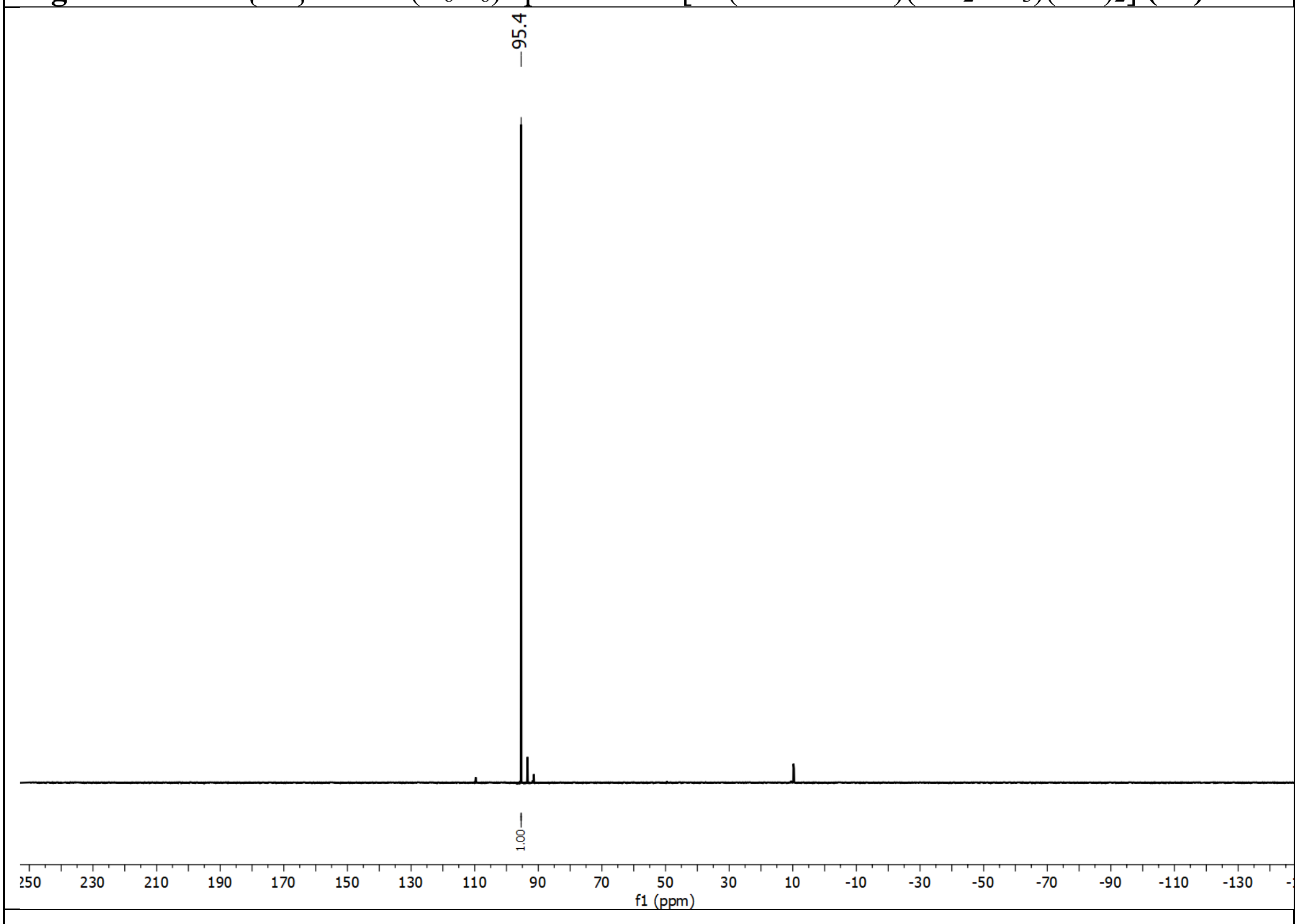

Figure S09. ^{1}H-NMR (C_6D_6) spectrum of [Fe(PCP^{CH2}-*i*Pr)($CH_2CH_2CH_3$)$(CO)_2$] **(3c)**

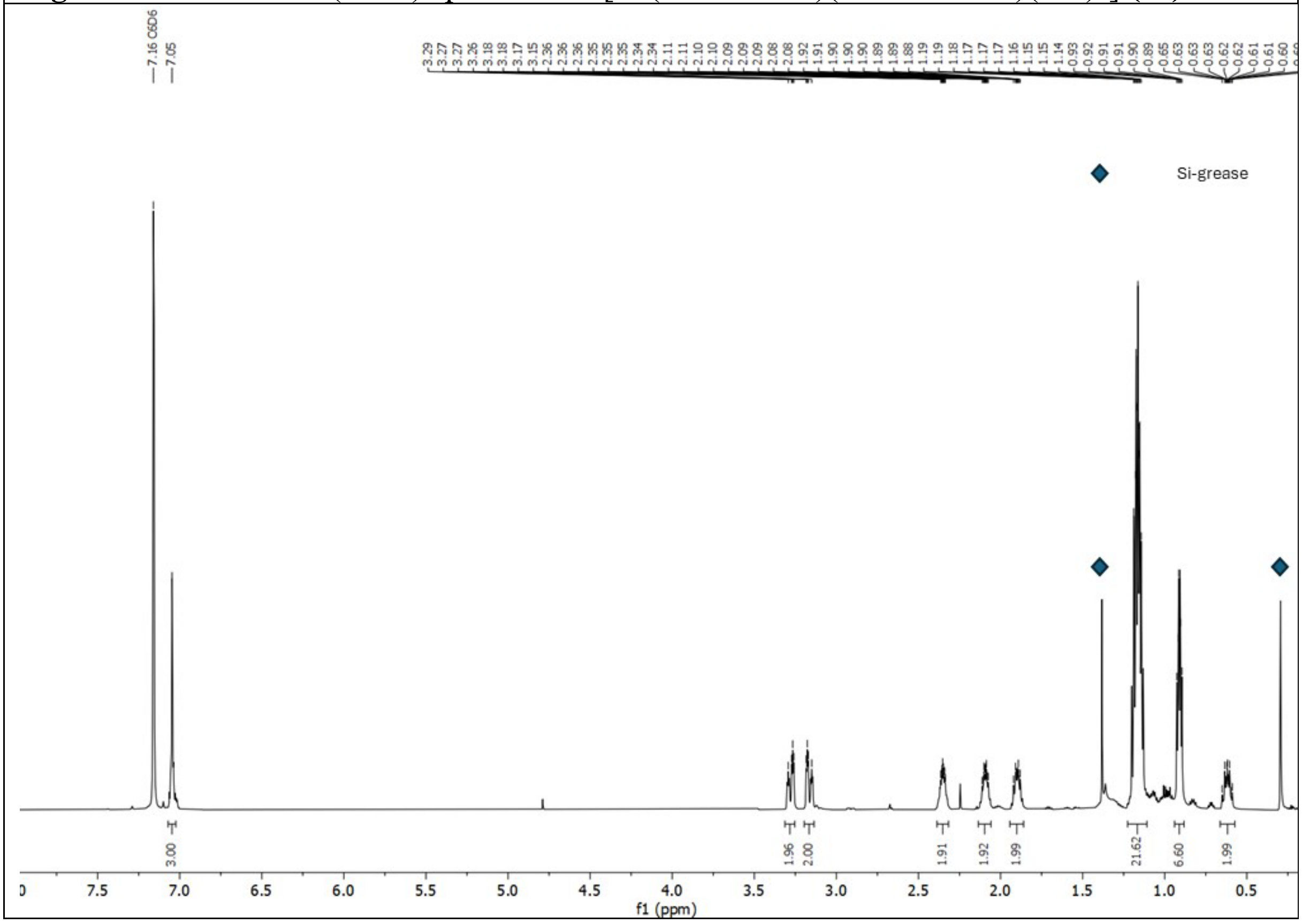

Figure S10. ^{13}C{^{1}H}-NMR (C_6D_6) spectrum of [Fe(PCP^{CH2}-*i*Pr)($CH_2CH_2CH_3$)$(CO)_2$] **(3c)**

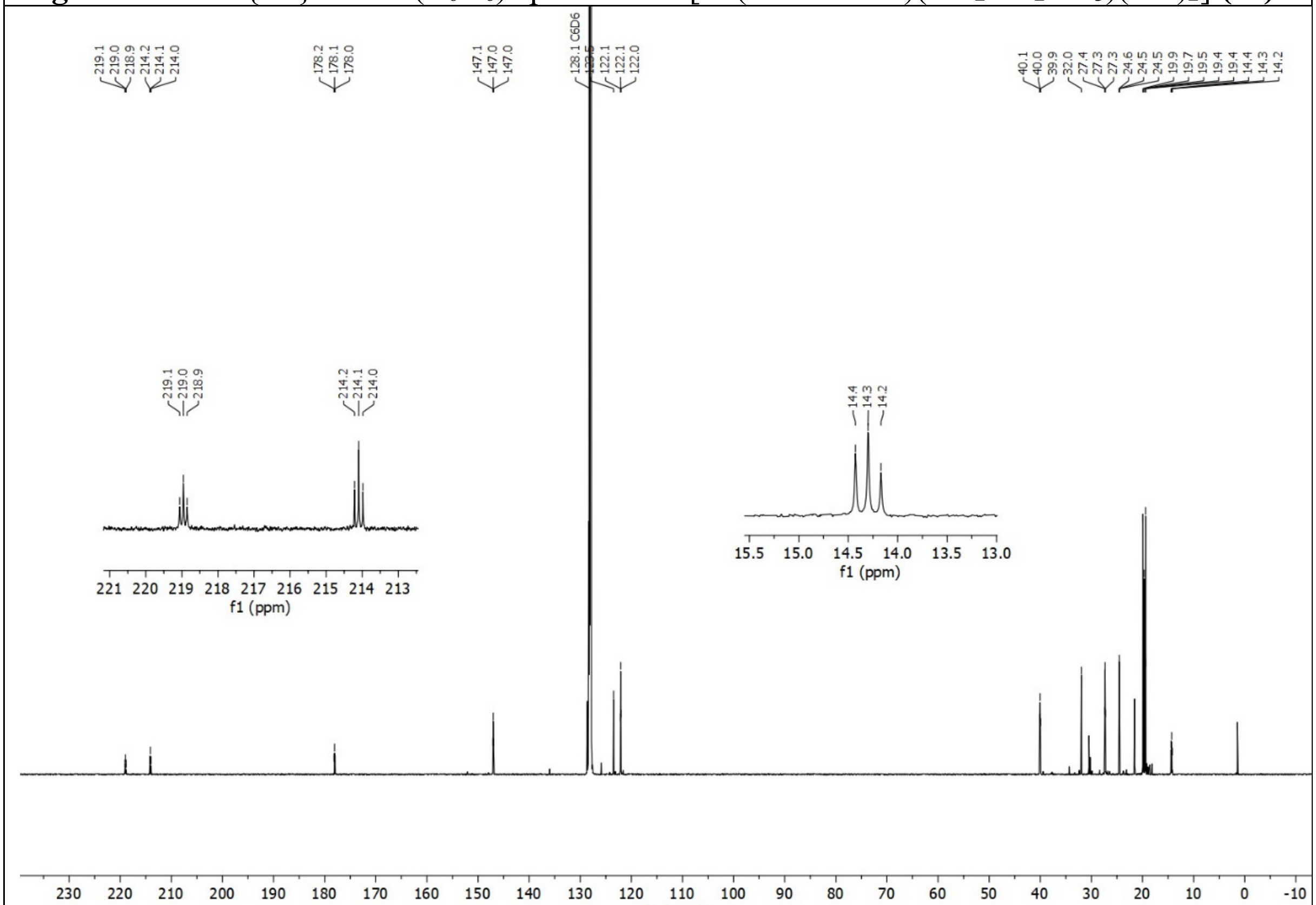

Figure S11. $^{31}P\{^1H\}$-NMR (C_6D_6) spectrum of [Fe(PCP^{CH2}-*i*Pr)($CH_2CH_2CH_3$)$(CO)_2$] **(3c)**

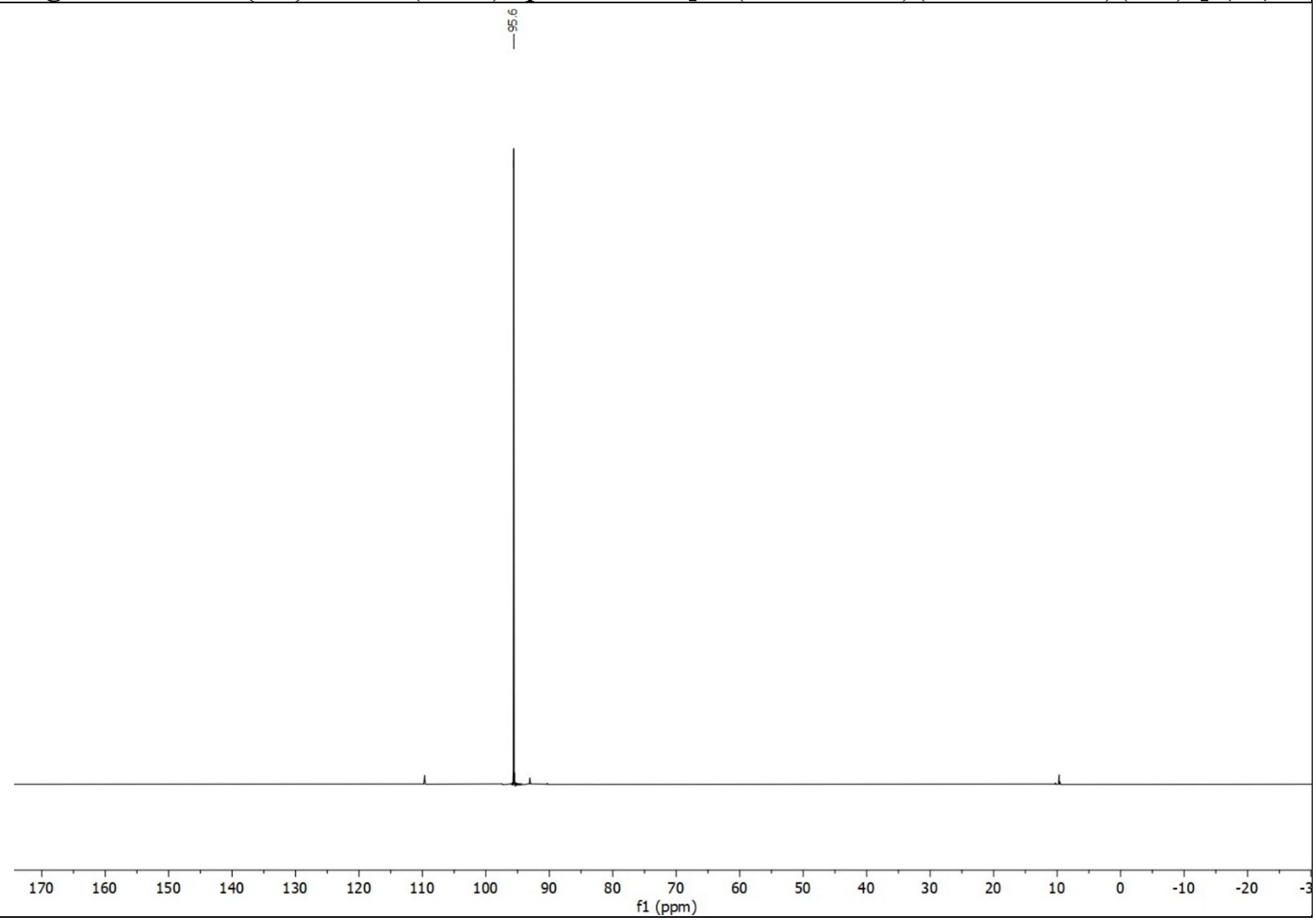

Figure S12. ^1H-NMR (C_6D_6) spectrum of [Fe(PCP^{CH2}-*i*Pr)($CH_2CH_2CH_2CH_3$)$(CO)_2$] **(3d)**

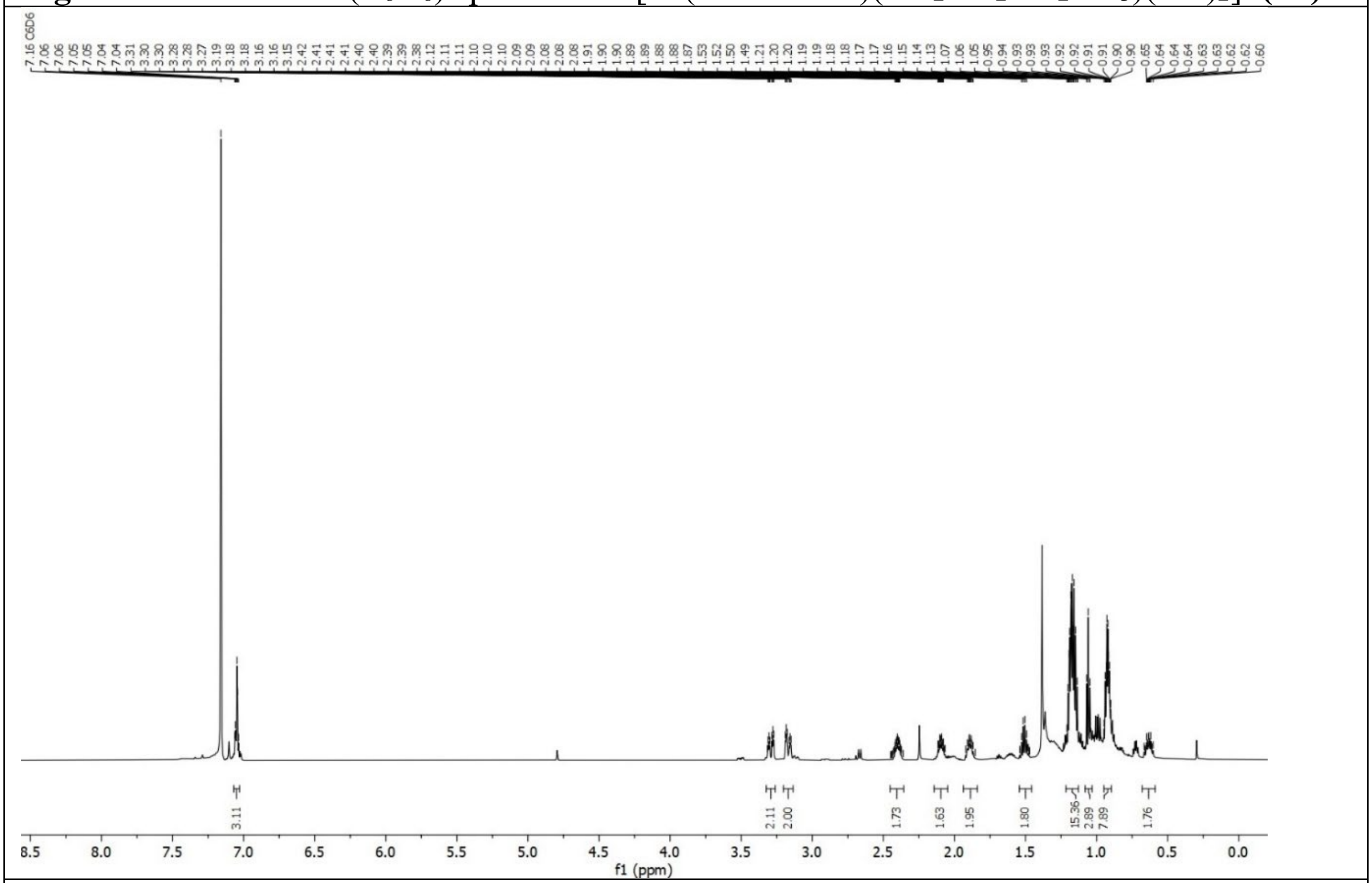

Figure S13. $^{13}C\{^1H\}$-NMR (C_6D_6) spectrum of [Fe(PCP^{CH2}-*i*Pr)($CH_2CH_2CH_2CH_3$)$(CO)_2$] **(3d)**

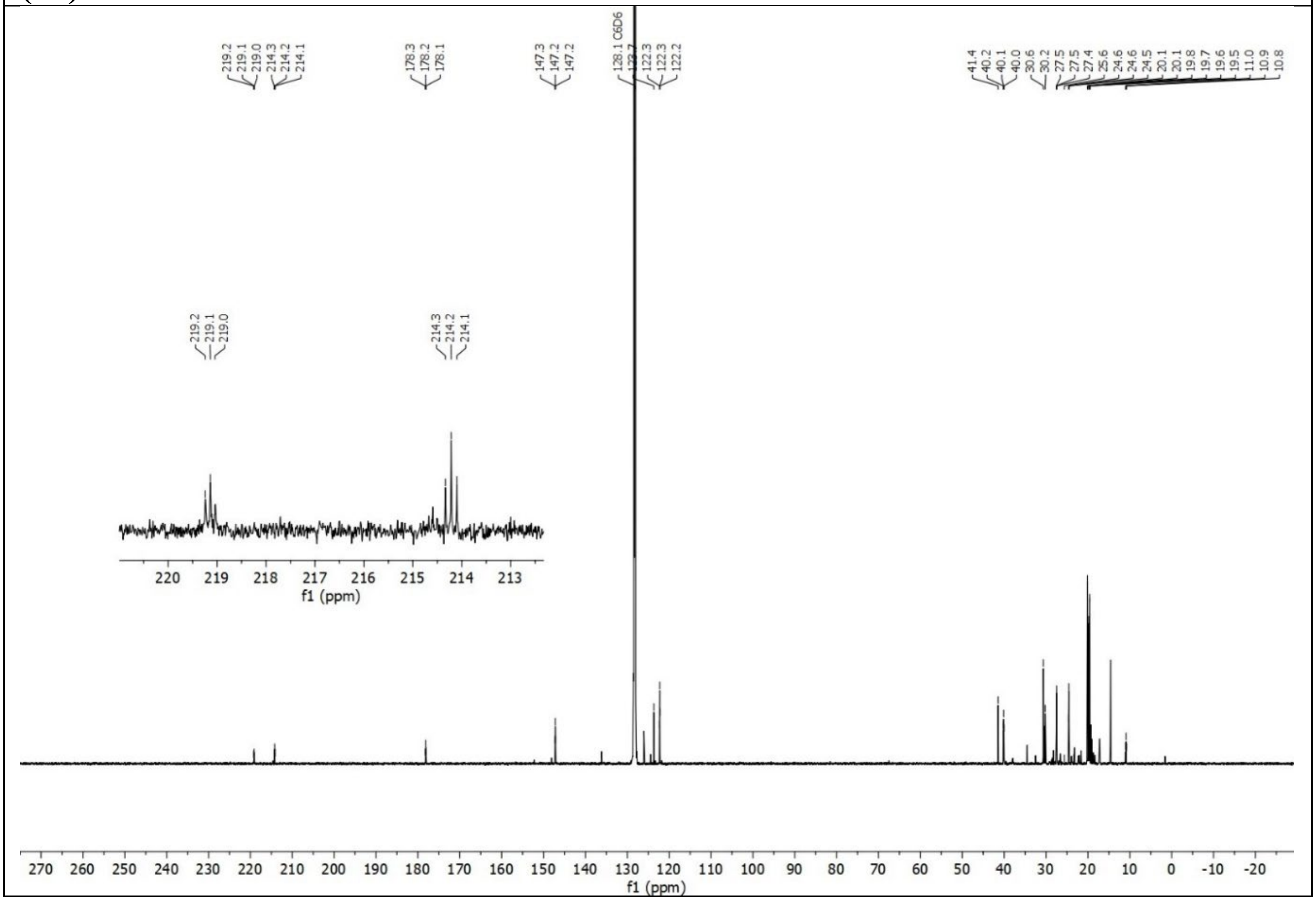

Figure S14. ^{31}P $\{^1H\}$-NMR (C_6D_6) spectrum of [Fe(PCP^{CH2}-*i*Pr)($CH_2CH_2CH_2CH_3$)$(CO)_2$] **(3d)**

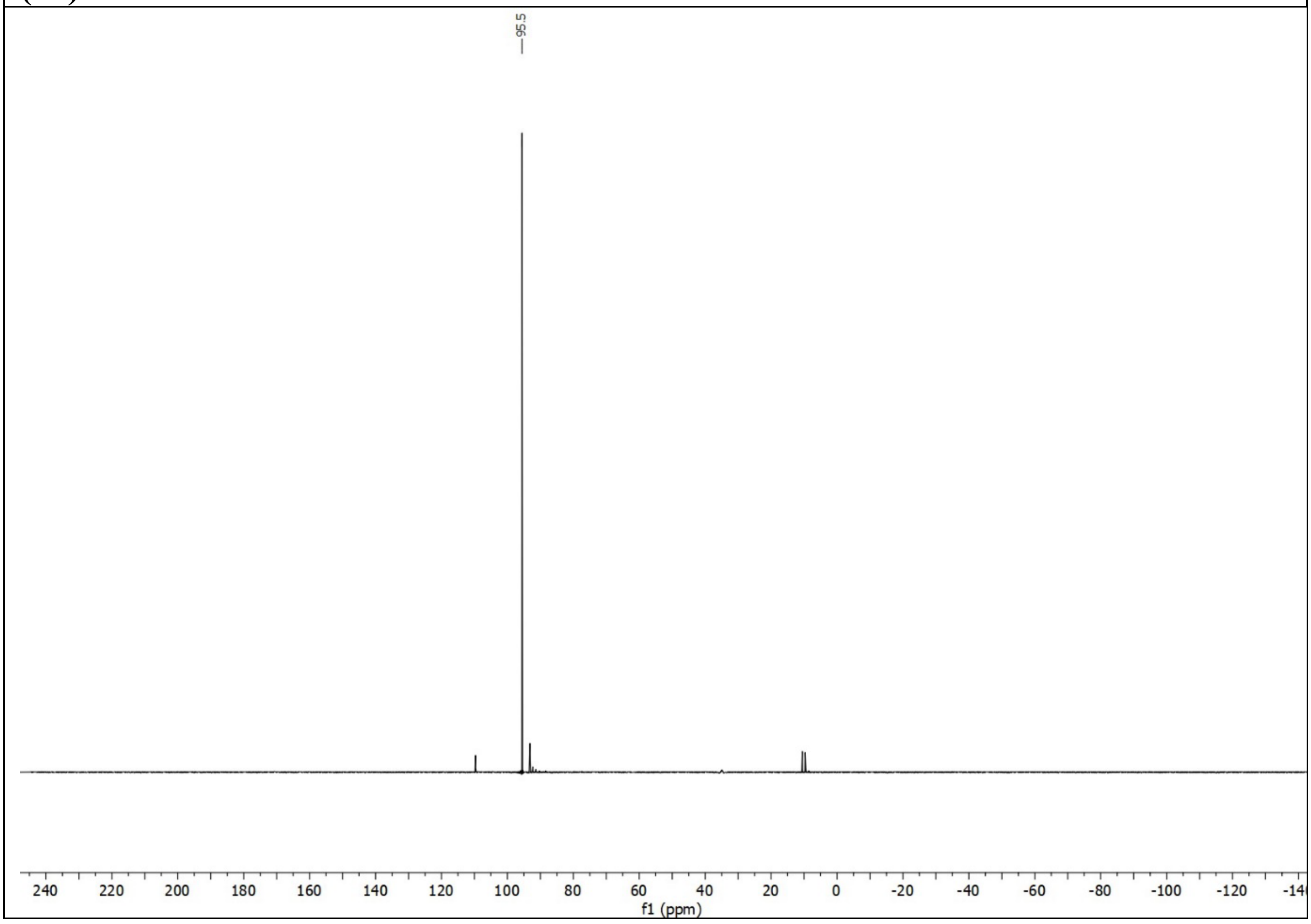

Figure S15. ^{1}H-NMR (C_6D_6) spectrum of [Fe(PCP^{CH2}-*i*Pr)(C(=O)-$CH_2CH_2CH_3$)$(CO)_2$] **(4)**

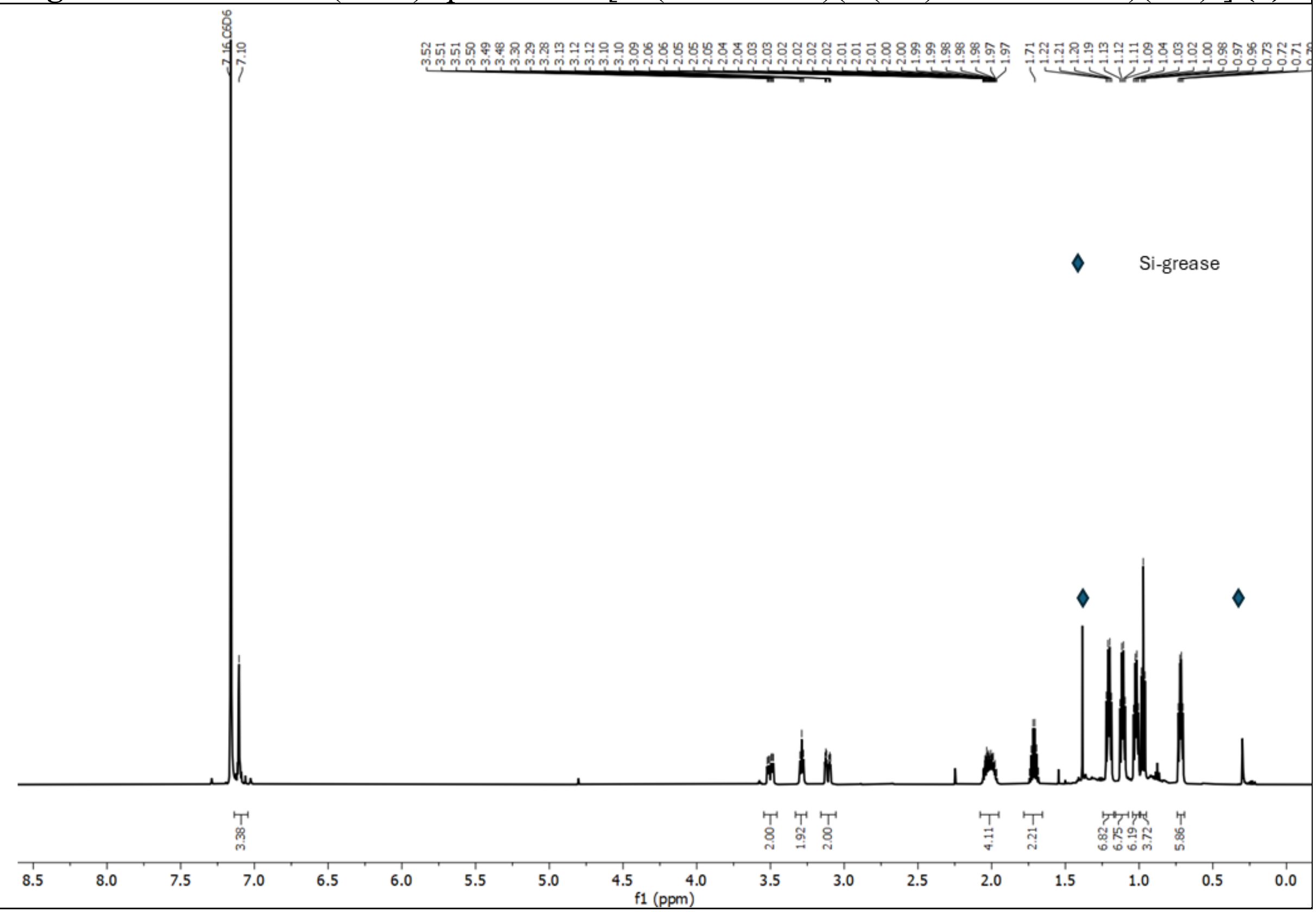

Figure S16. $^{13}C\{^{1}H\}$-NMR (C_6D_6) spectrum of [Fe(PCP^{CH2}-*i*Pr)(C(=O)-$CH_2CH_2CH_3$)$(CO)_2$] **(4)**

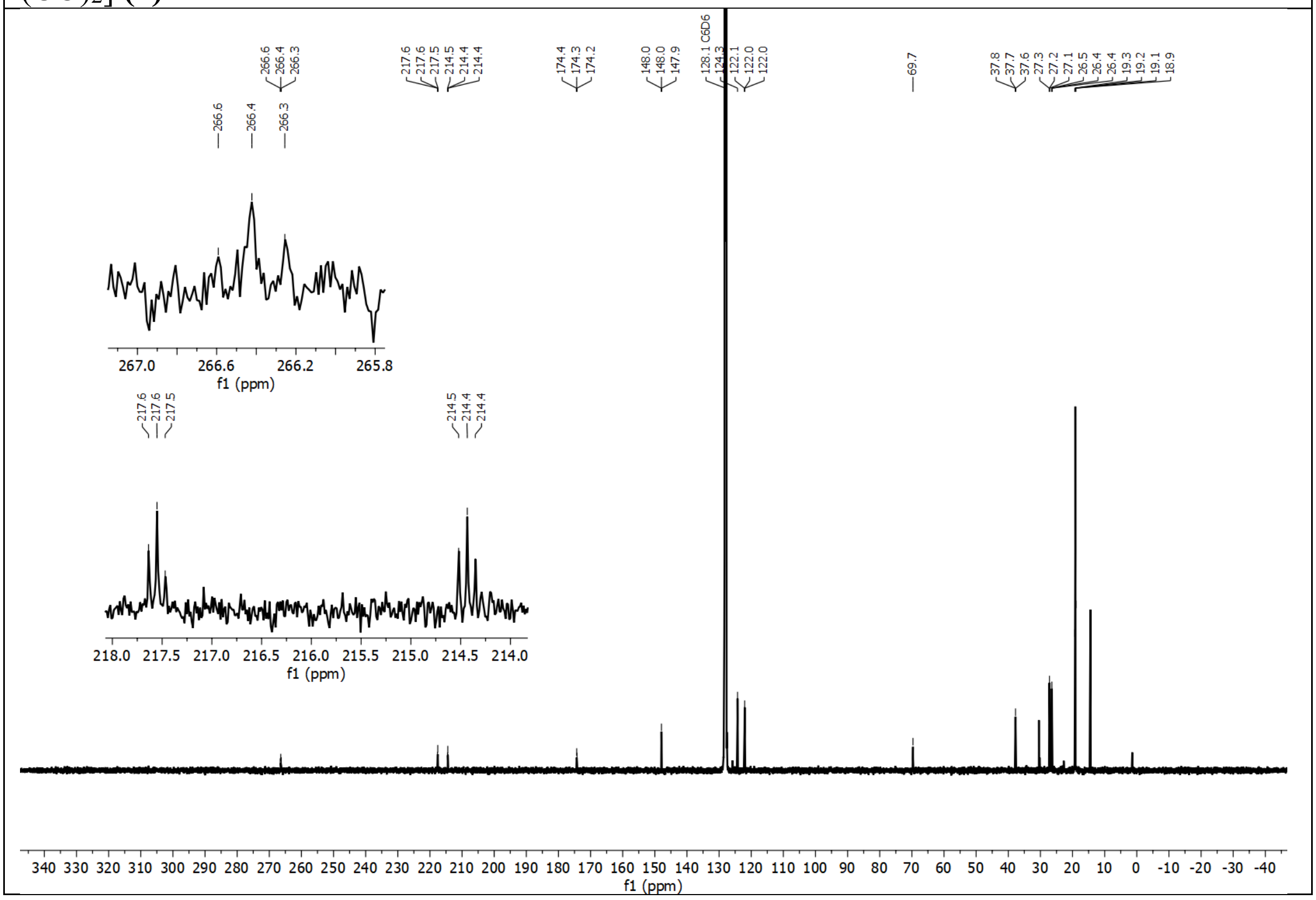

Figure S17. $^{31}P\{^{1}H\}$-NMR (C_6D_6) spectrum of [Fe(PCP^{CH2}-*i*Pr)(C(=O)-$CH_2CH_2CH_3$)($CO)_2$] **(4)**

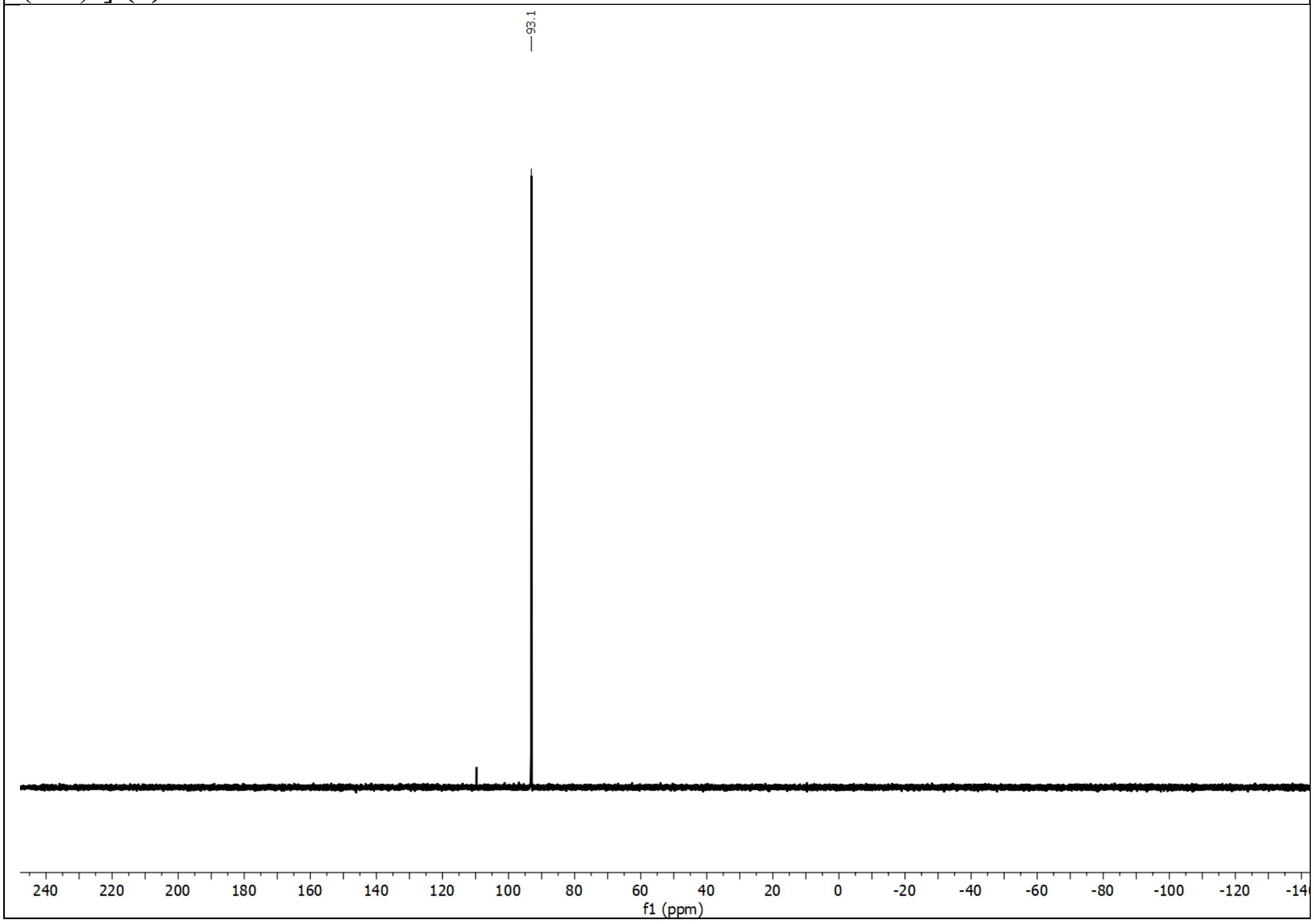

Figure S18. ^{1}H-NMR (C_6D_6)spectrum of [Fe(PCP^{CH2}-*i*Pr) (C(=O)-$CH_2CH_2CH_3$)(CN*t*Bu)(CO)] **(5)**

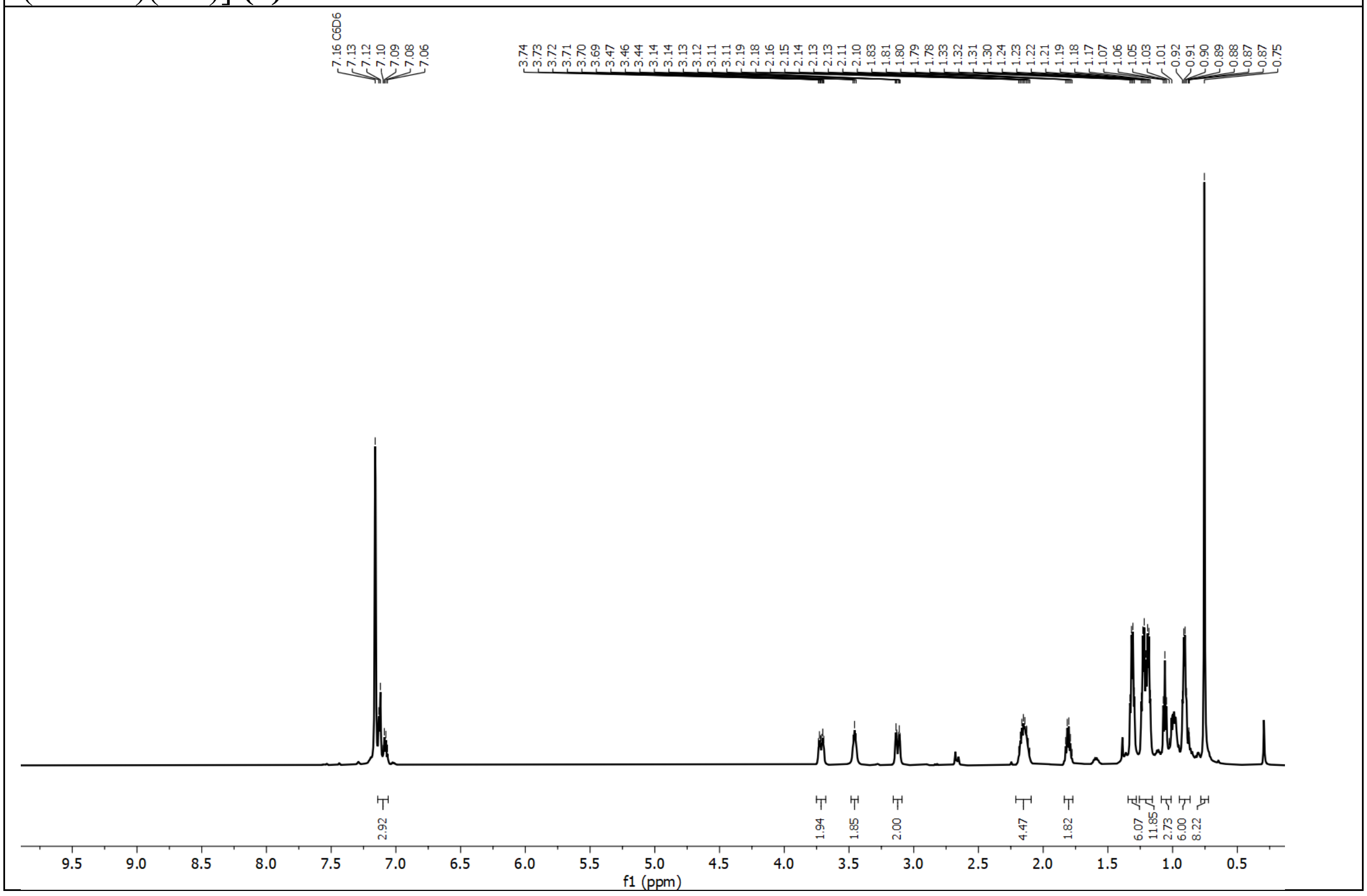

Figure S19. $^{13}C\{^{1}H\}$-NMR (C_6D_6) spectrum of [Fe(PCP^{CH2}-*i*Pr)(C(=O)$CH_2CH_2CH_3$)(CN*t*Bu)(CO)] **(5)**

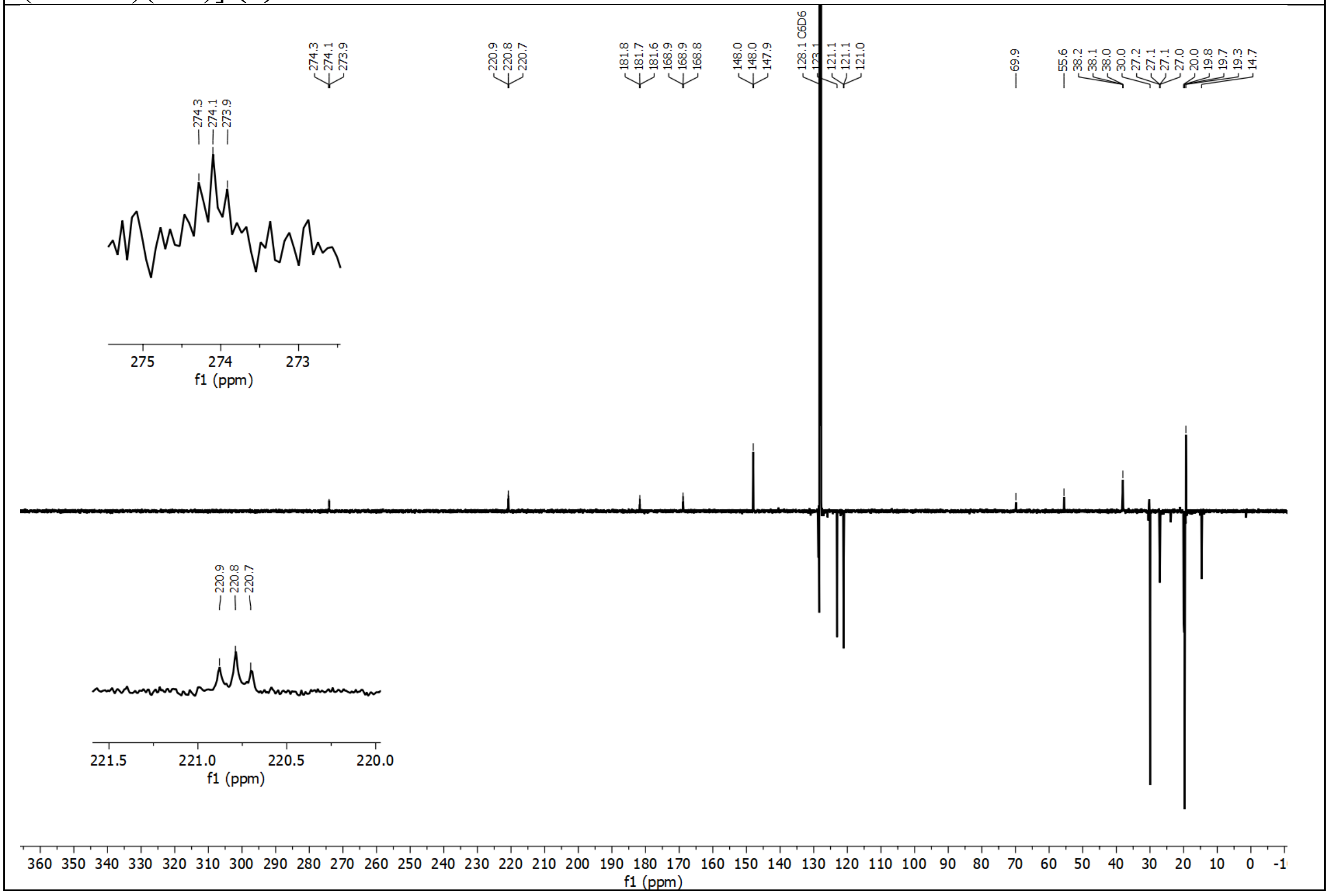

Figure S20. $^{31}P\ \{^{1}H\}$-NMR (C_6D_6) spectrum of [Fe(PCP^{CH2}-*i*Pr) (C(=O)-$CH_2CH_2CH_3$) (CN*t*Bu)(CO)] **(5)**

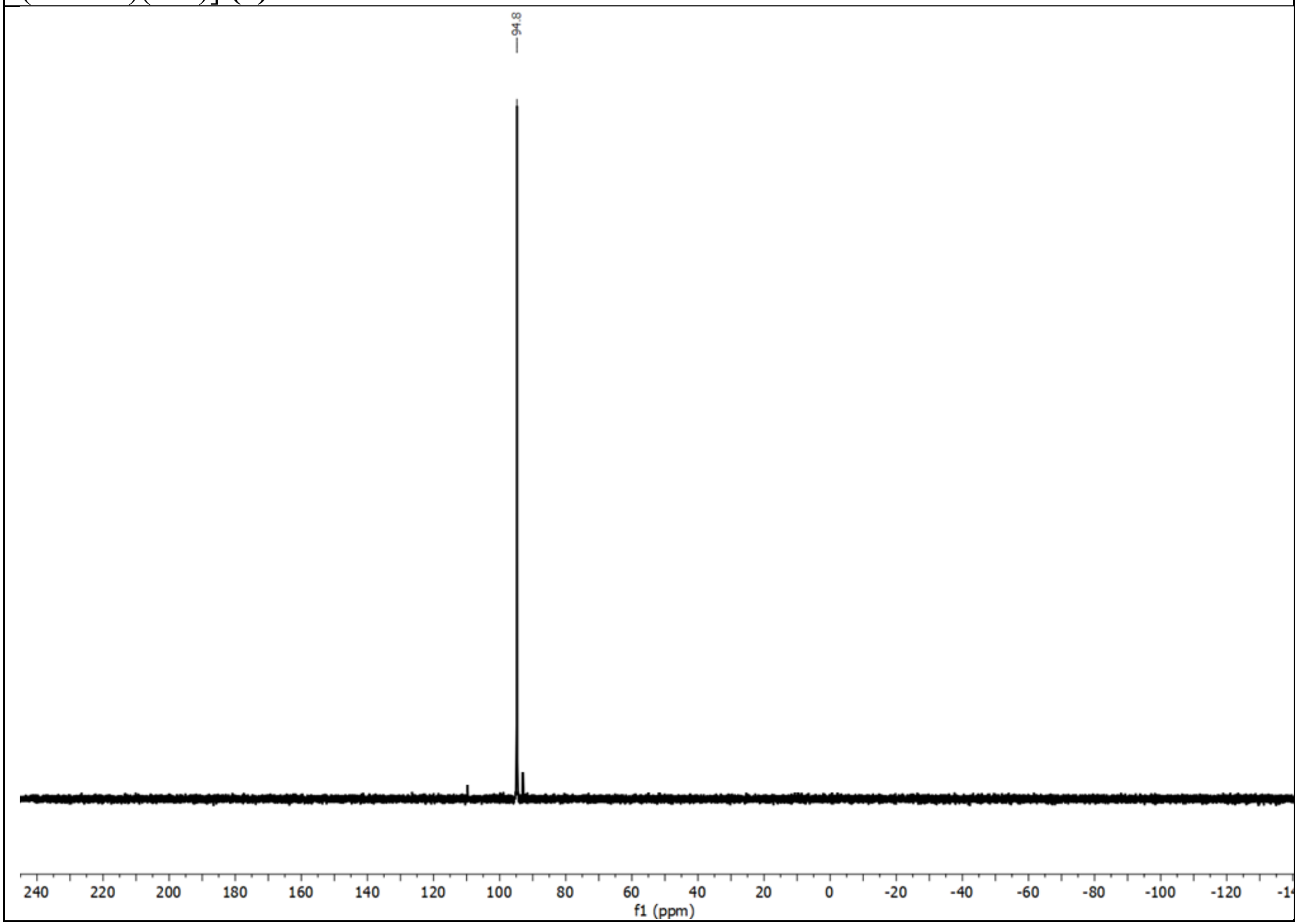

Figure S21. ^{1}H-NMR (C_6D_6) spectrum of [Fe(PCP^{CH2}-*i*Pr)(H)(CO)$_2$] **(6)**

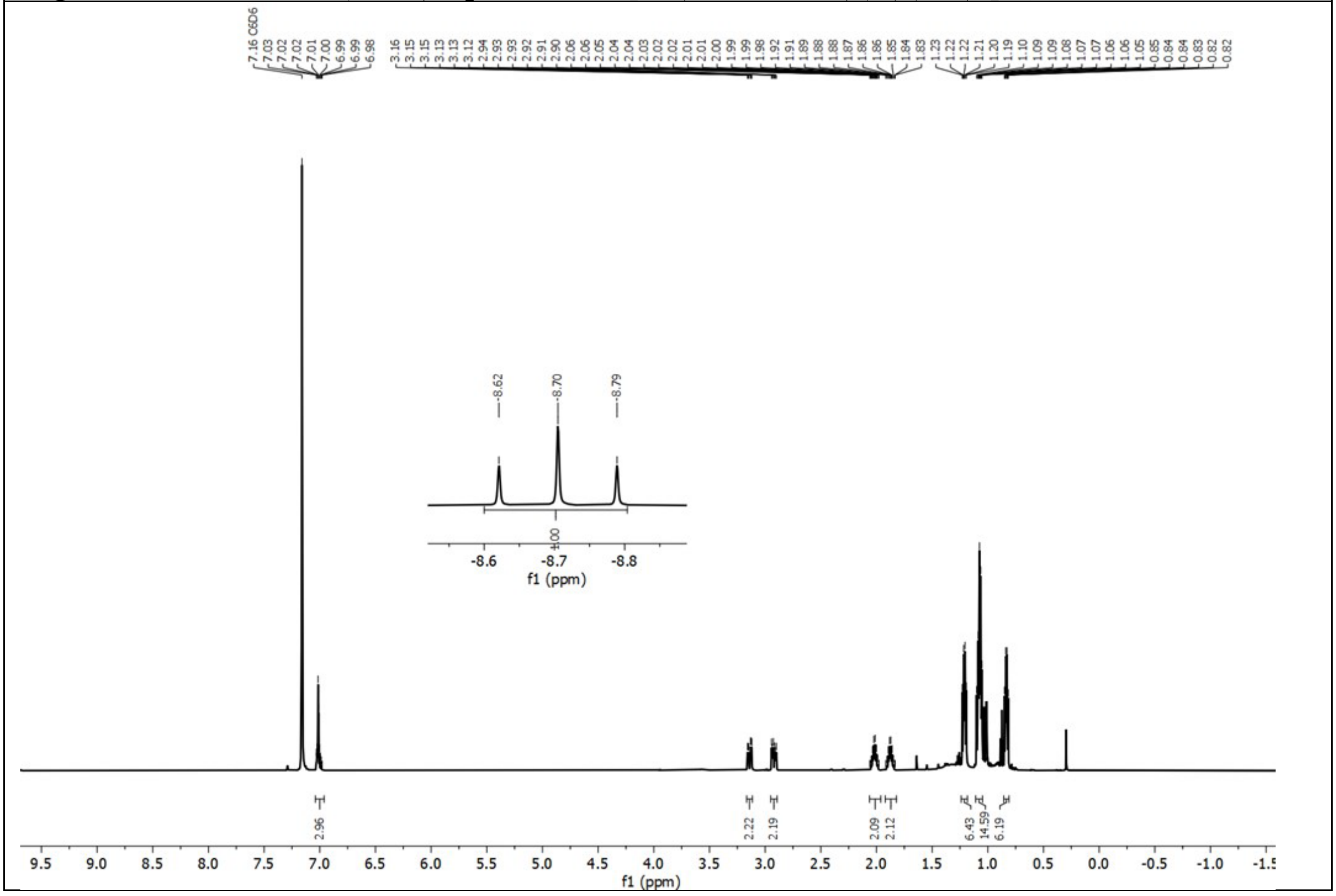

Figure S22. ^{13}C{^{1}H}-APT-NMR (C_6D_6) spectrum of [Fe(PCP^{CH2}-*i*Pr)(H)(CO)$_2$] **(6)**

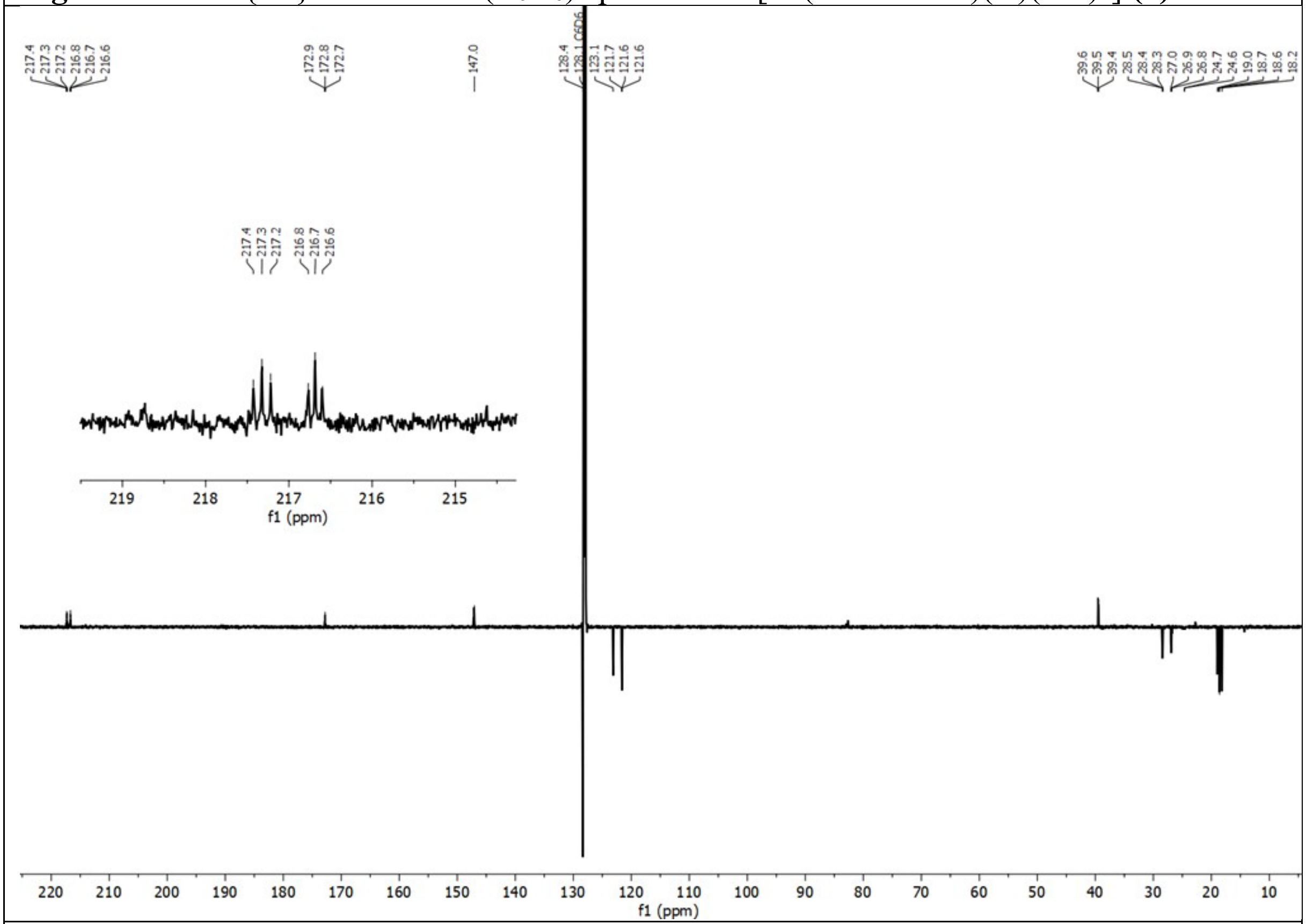

Figure S23. $^{31}P\{^1H\}$-NMR (C_6D_6) spectrum of [Fe(PCP^{CH2}-*i*Pr)(H)(CO)$_2$] **(6)**

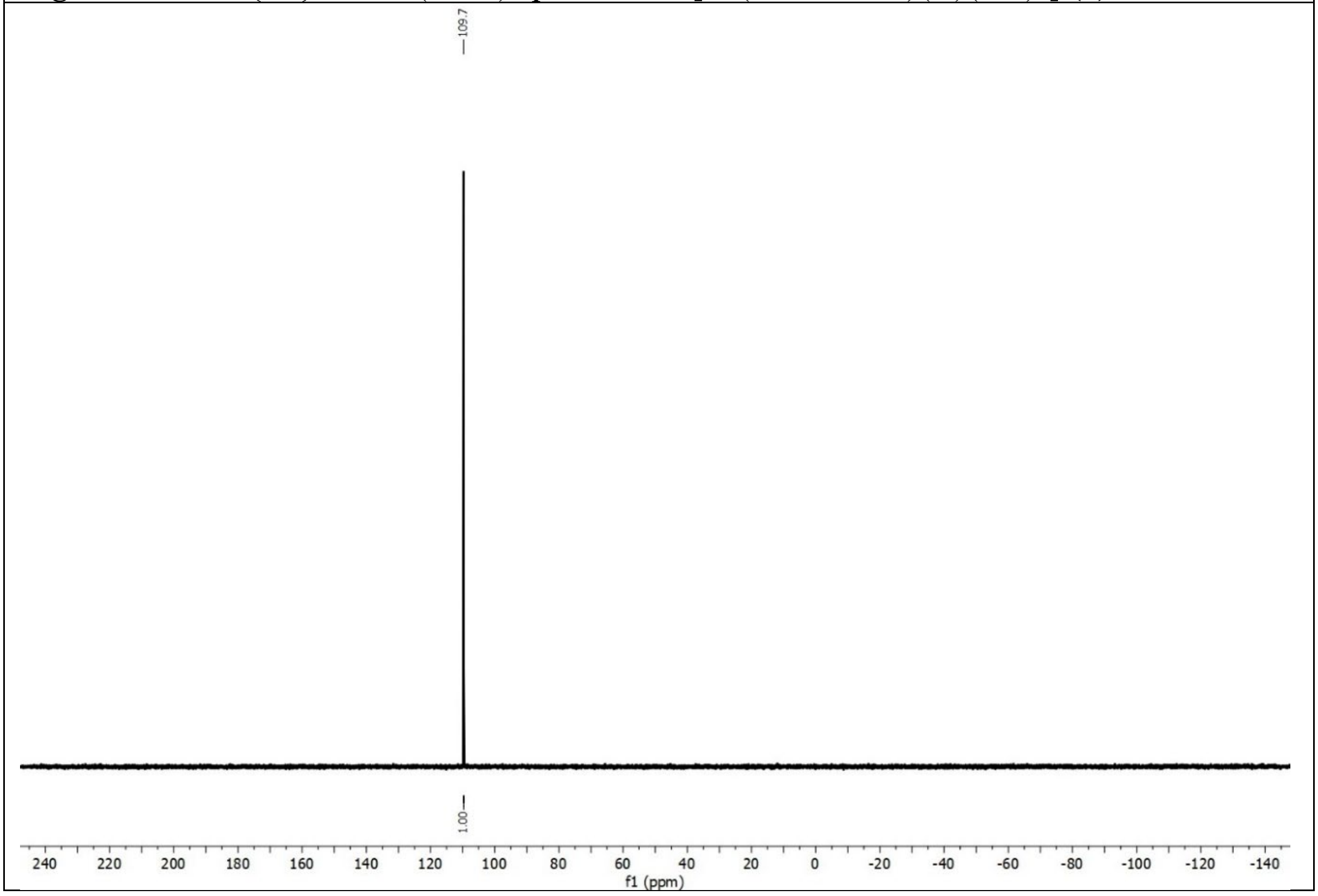

Figure S24. ^1H-NMR (C_6D_6) spectrum of [Fe(PCP^{CH2}-*i*Pr)(κ^2-H_2BPin)(CO)] **(7)**

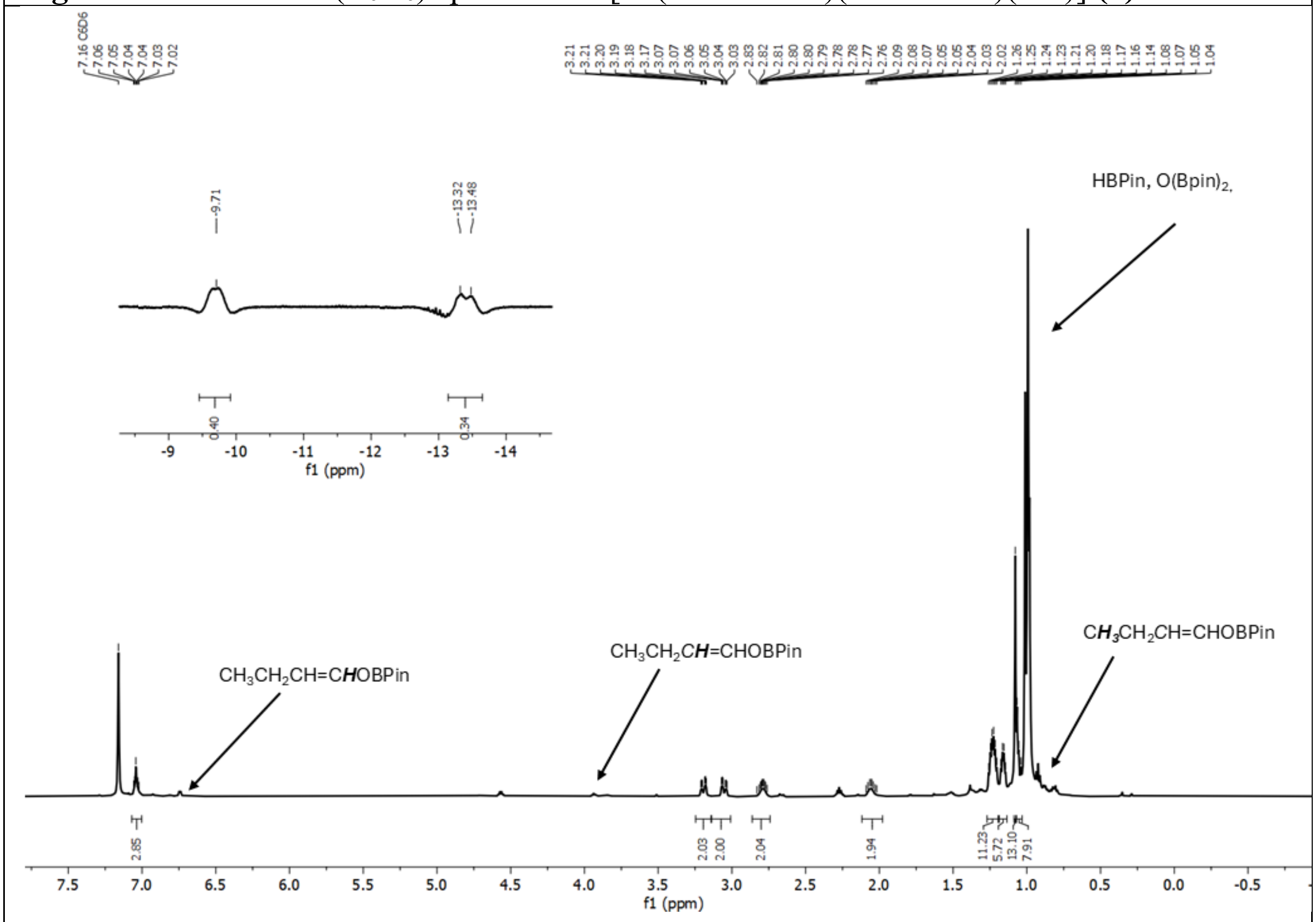

Figure S25. $^{13}C\{^1H\}$-APT-NMR (C_6D_6) spectrum of [Fe(PCP^{CH2}-*i*Pr)(κ^2-H_2BPin)(CO)] **(7)**

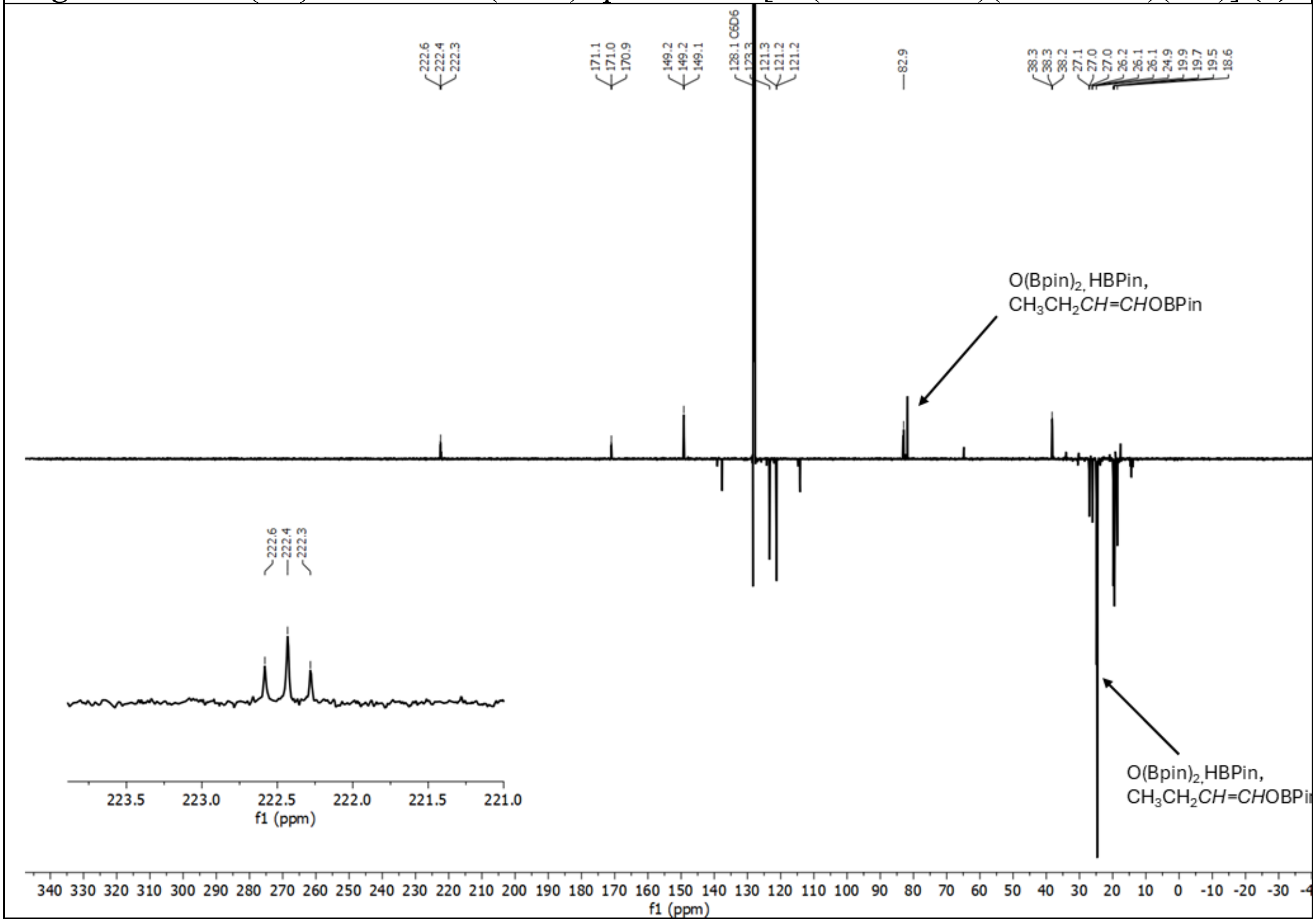

Figure S26. $^{31}P\{^1H\}$-NMR (C_6D_6) spectrum of [Fe(PCP^{CH2}-*i*Pr)(κ^2-H_2BPin)(CO)] **(7)**

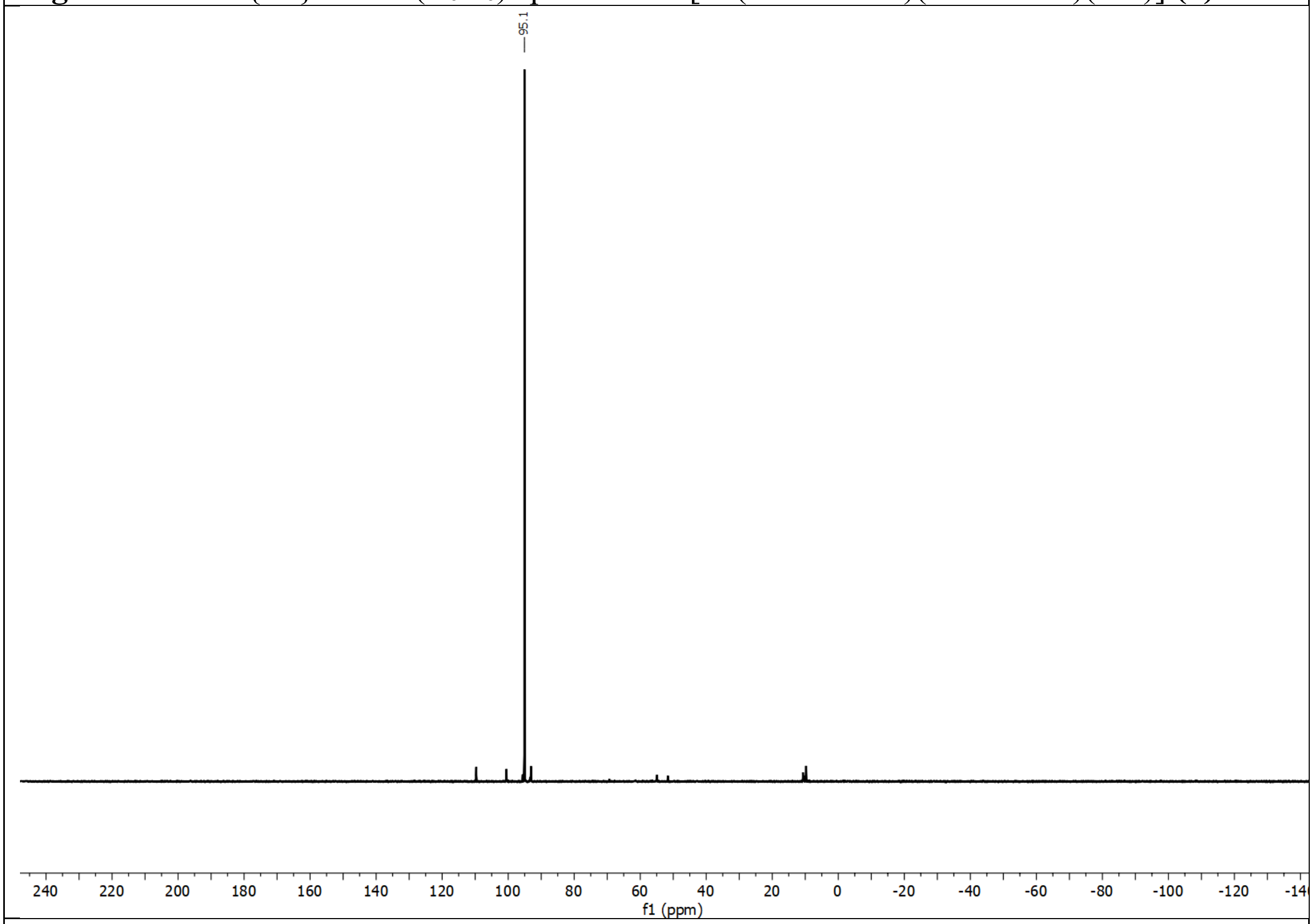

Figure S27. $^{11}B\{^1H\}$-NMR (C_6D_6) spectrum of [Fe(PCP^{CH2}-*i*Pr)(κ^2-H_2BPin)(CO)] **(7)**

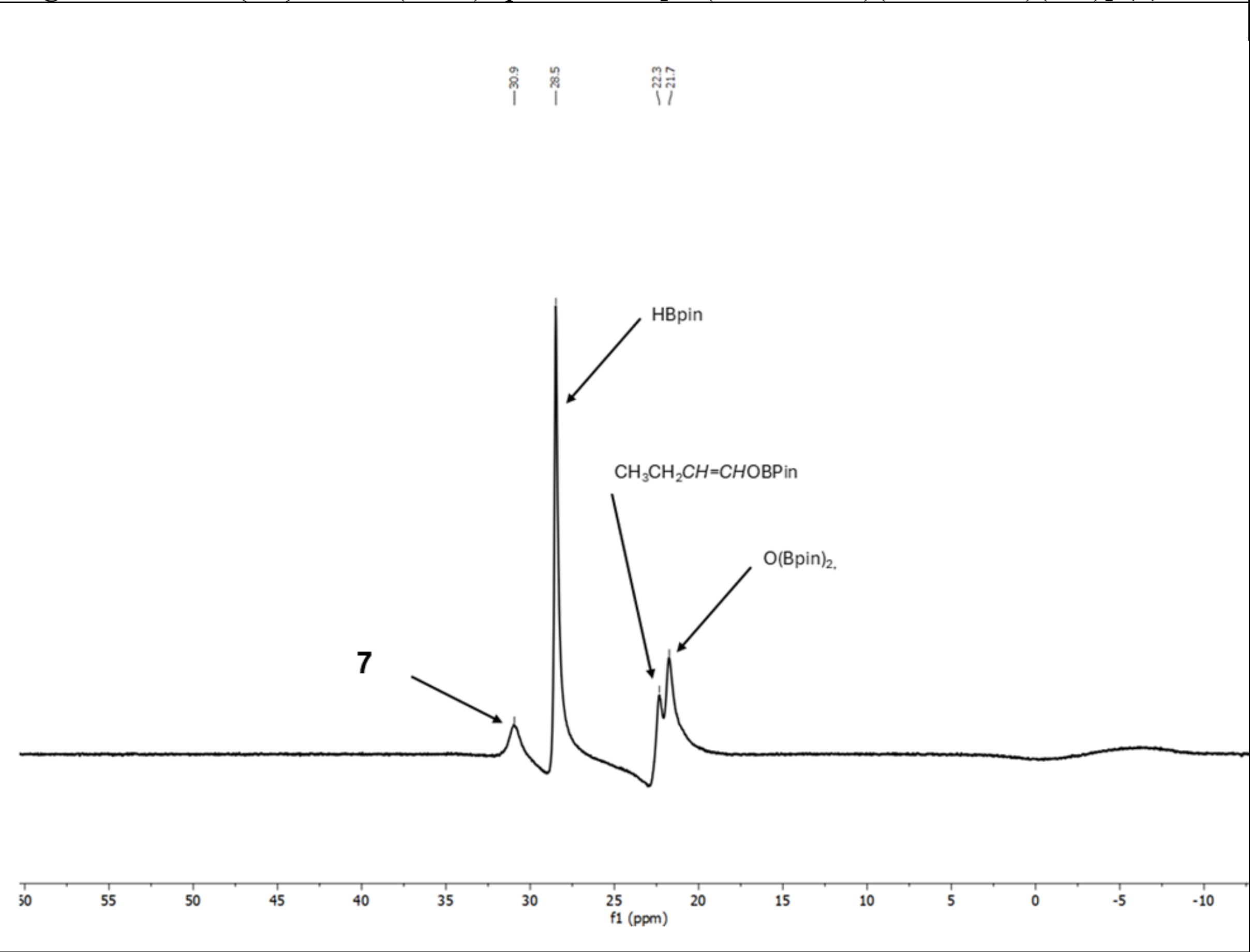

Figure S28: ^1H-NMR (C_6D_6) spectrum of [Fe(PCP^{CH2}-*i*Pr)(H)(P(OMe)$_3$(CO)] (**8**)

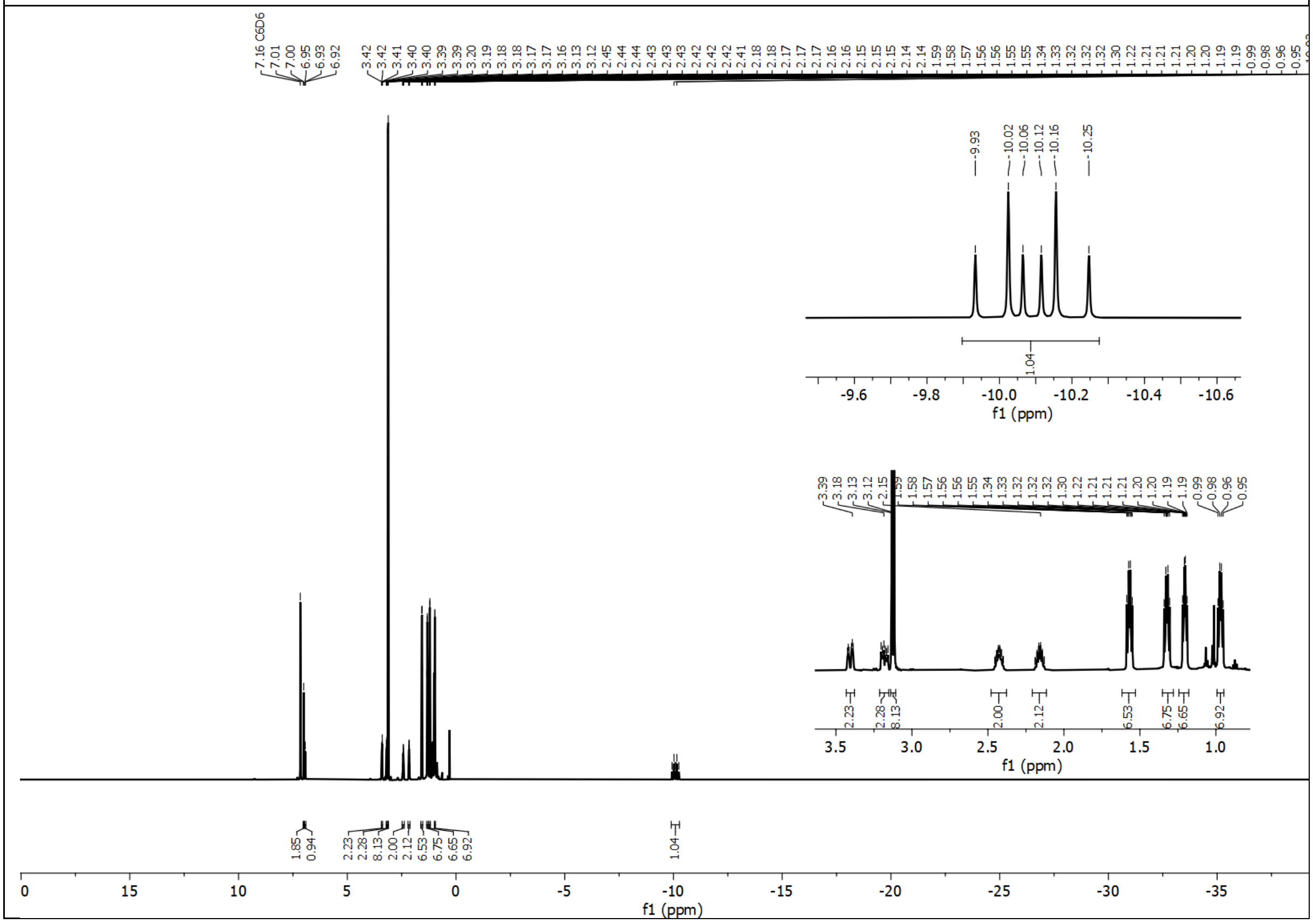

Figure S29: $^{13}C\{^1H\}$- APT-NMR (C_6D_6) spectrum of [Fe(PCP^{CH2}-*i*Pr)(H)(P(OMe)$_3$(CO)] (**8**)

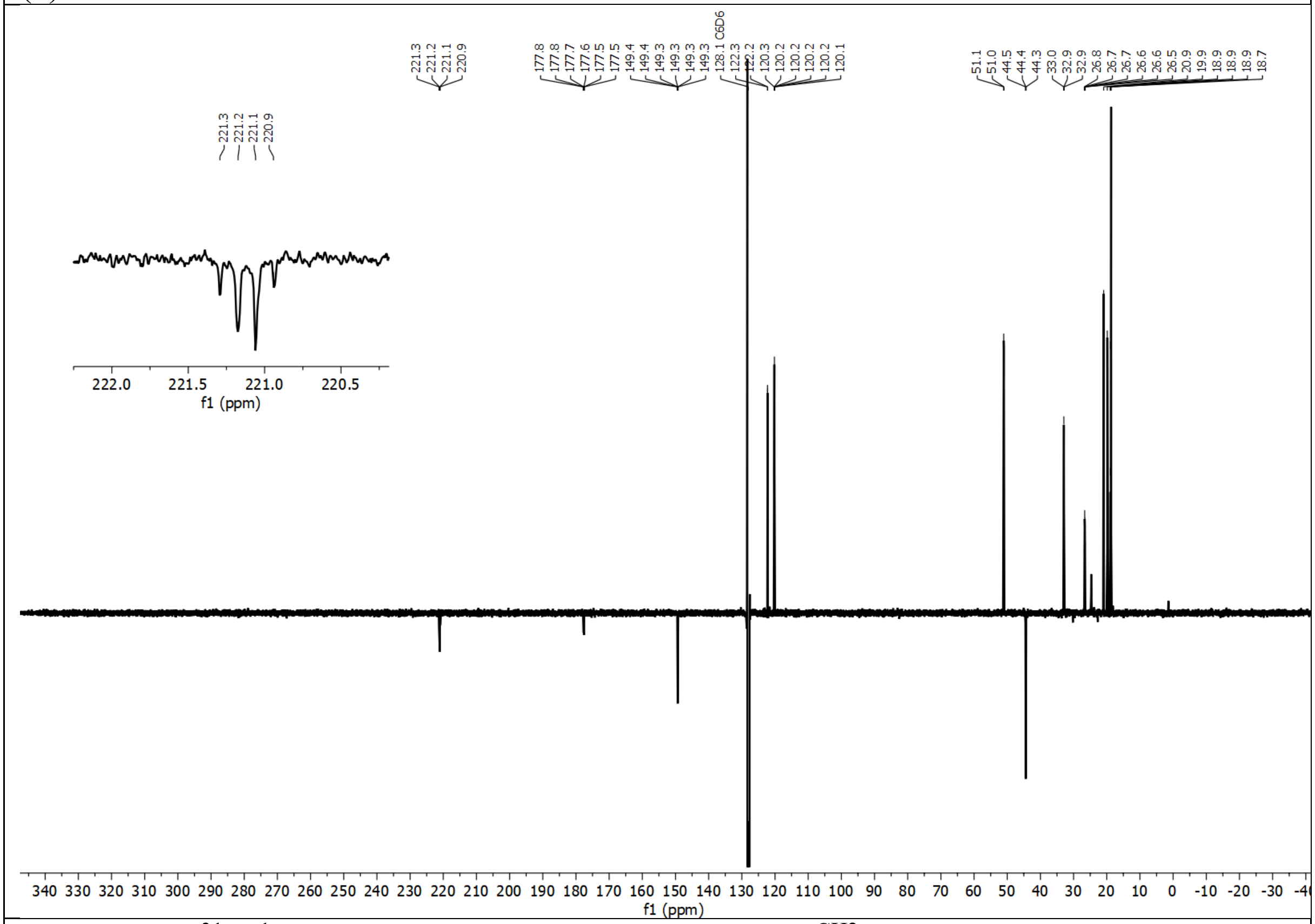

Figure S30: $^{31}P\{^1H\}$ -NMR (C_6D_6) spectrum of [Fe(PCP^{CH2}-*i*Pr)(H)(P(OMe)$_3$(CO)] (**8**)

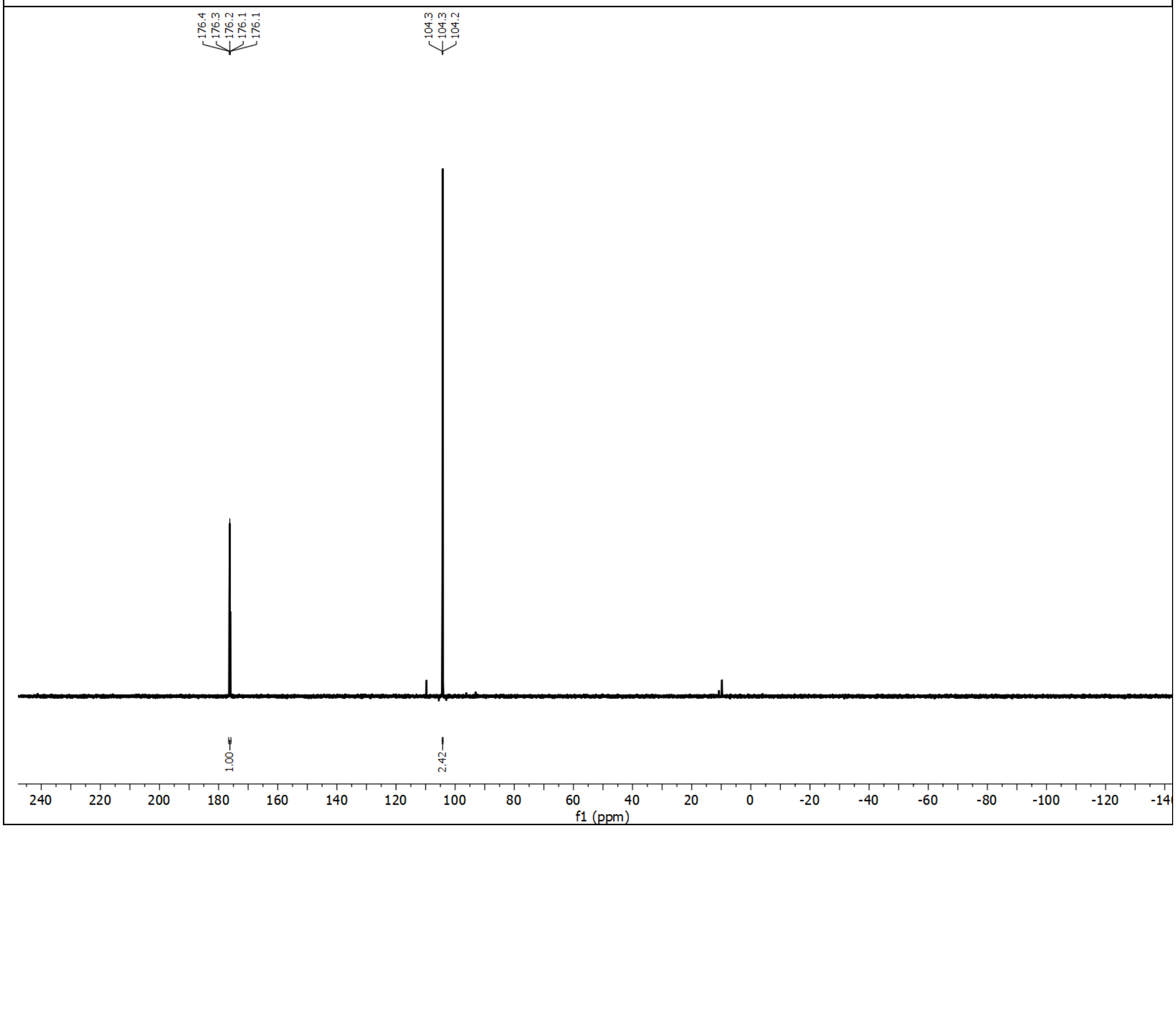

Figure S31. ^{1}H-NMR (C_6D_6) spectrum of [Fe(PCP^{CH2}-*i*Pr)(SiHPh$_2$)(CO)] (**9**)

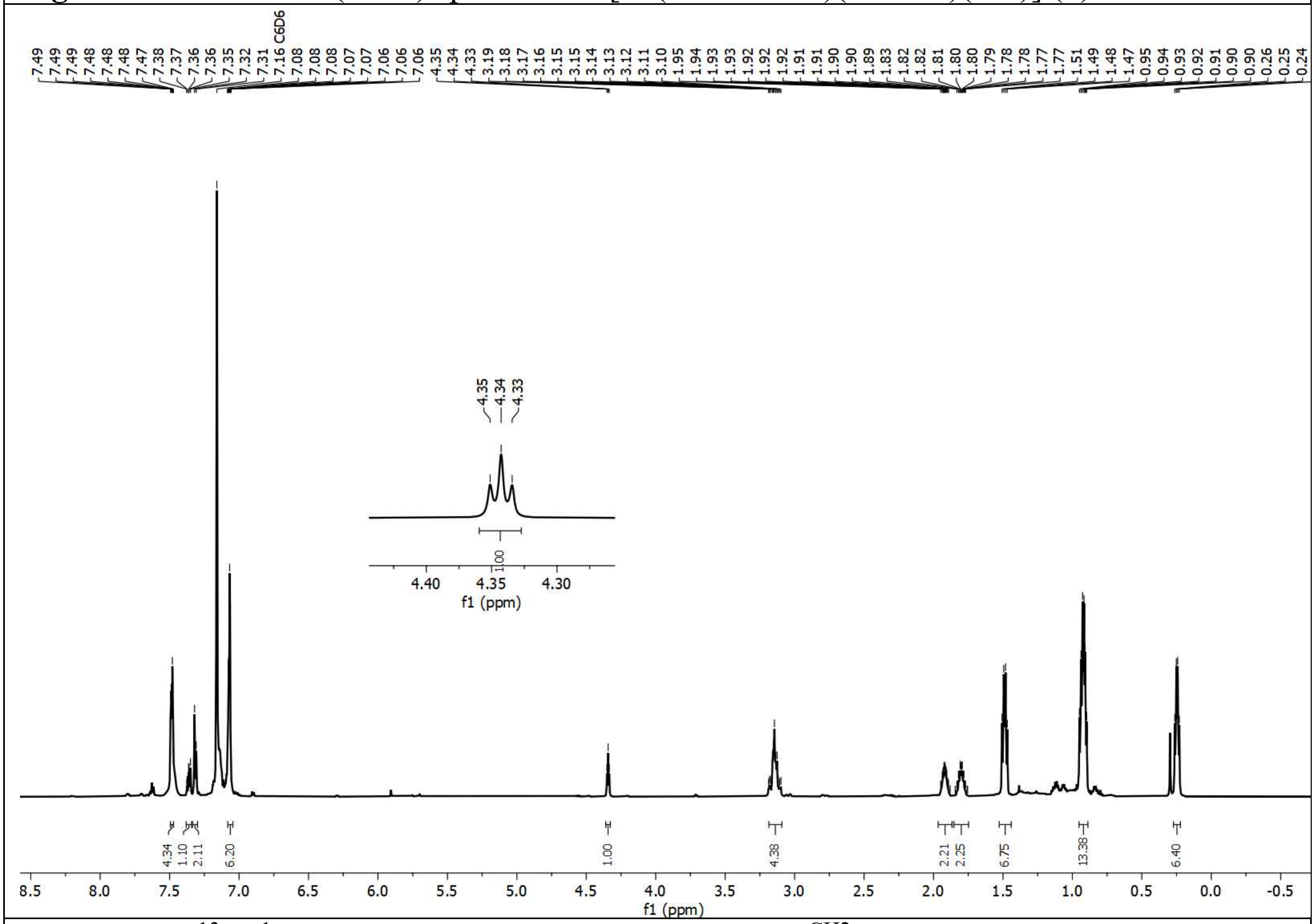

Figure S32. ^{13}C{^{1}H}-NMR (C_6D_6) spectrum of [Fe(PCP^{CH2}-*i*Pr)(SiHPh$_2$)(CO)] (**9**)

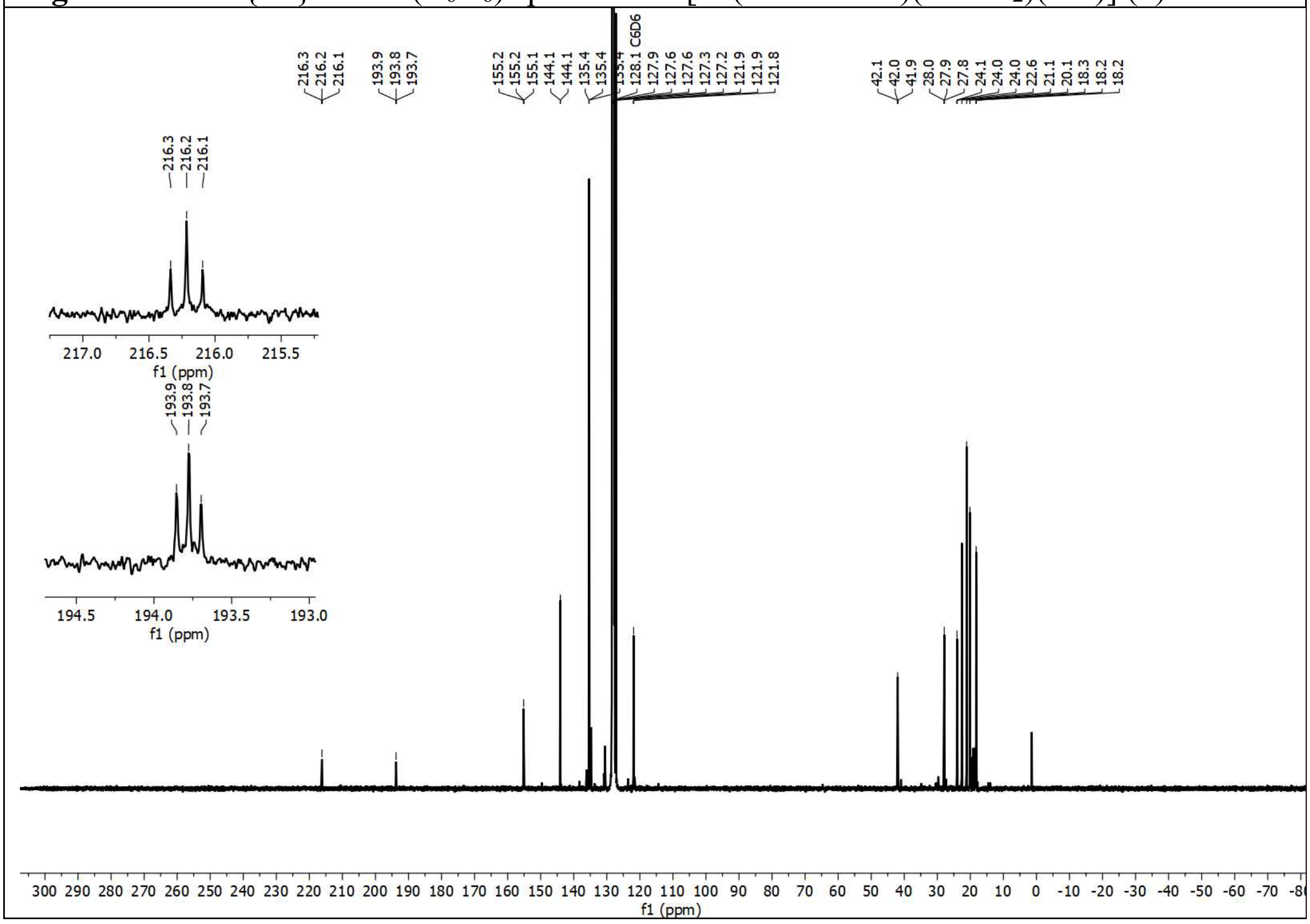

Figure S33. $^{31}P\{^1H\}$-NMR (C_6D_6) spectrum of [Fe(PCP^{CH2}-*i*Pr)(SiHPh$_2$)(CO)] (**9**)

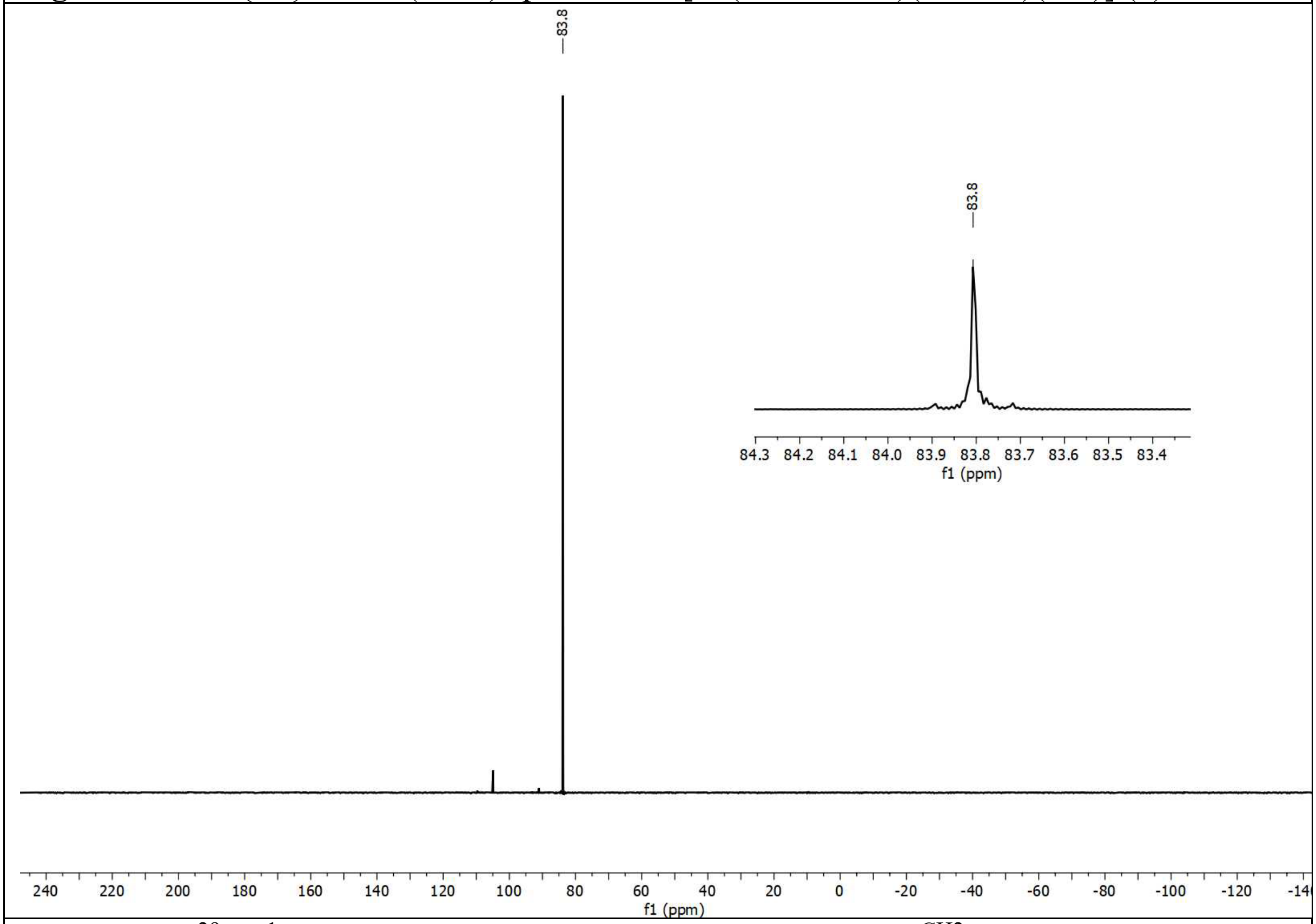

Figure S34. $^{29}Si\{^1H\}$-INEPT-NMR (C_6D_6) spectrum of [Fe(PCP^{CH2}-*i*Pr)(SiHPh$_2$)(CO)] (**9**)

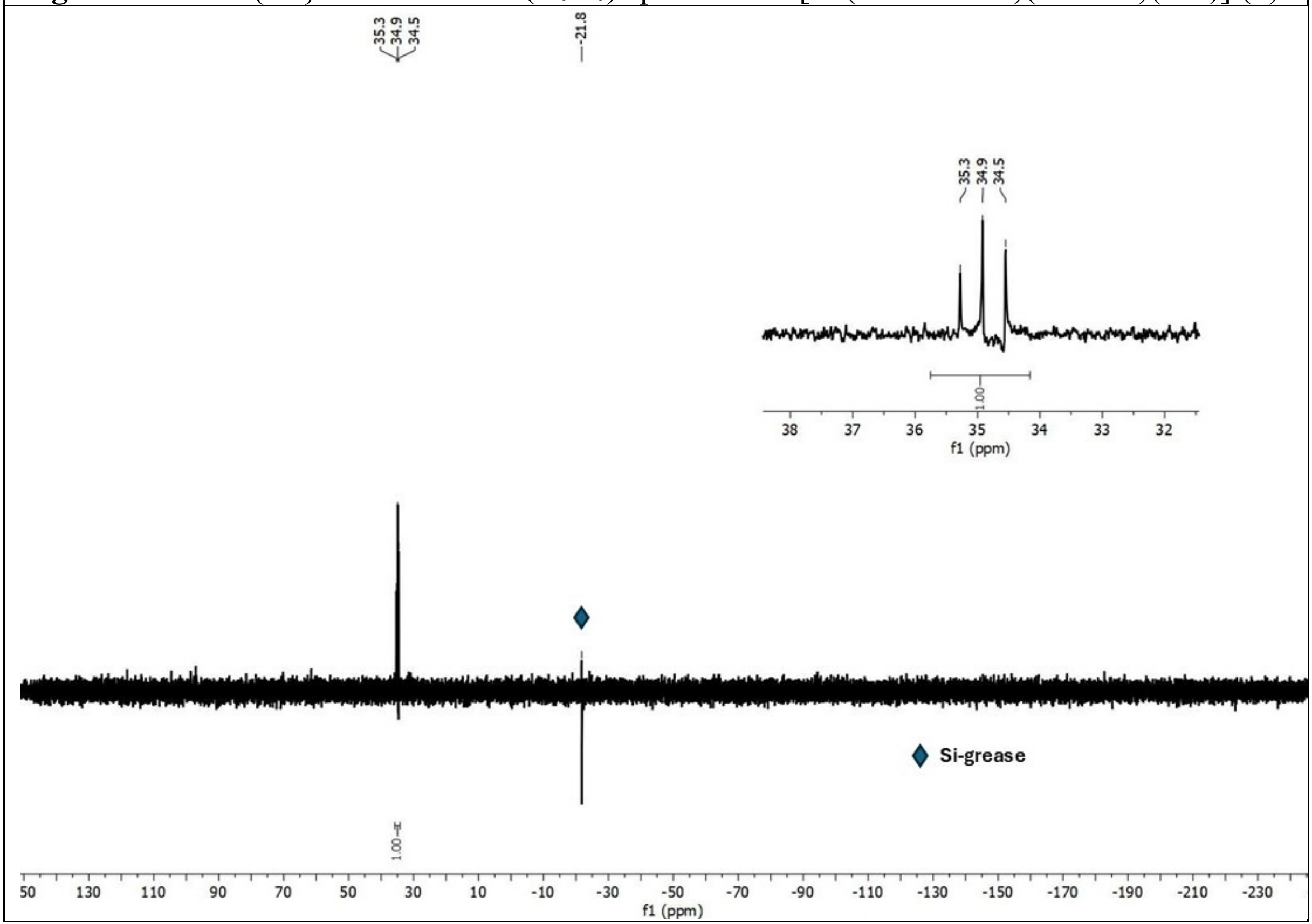

Supplement: Supplementary file 1 [file om6c00256_si_002.pdf]
